# Why do women still give birth at home; perceptions of Pakistani women and decision-makers from marginalized communities

**DOI:** 10.1371/journal.pgph.0002217

**Published:** 2023-10-13

**Authors:** Ayesha Khalid, Kaniz Amna Haider, Hareem Ahmer, Sahir Noorani, Zahra Hoodbhoy

**Affiliations:** 1 Department of Pediatrics and Child Health, The Aga Khan University, Karachi, Pakistan; 2 Data and Digital Department, Vital Pakistan Trust, Karachi, Pakistan; Johns Hopkins University Bloomberg School of Public Health, UNITED STATES

## Abstract

In low- and middle-income countries (LMICs), maternal and newborn mortality is high due to the high prevalence of home births. Understanding the reasons behind this behavior is essential for improving maternal and newborn outcomes. Therefore, a qualitative exploratory study was conducted in a peri-urban community in Karachi, Pakistan to understand the perceptions of pregnant women who delivered at home despite receiving antenatal care and the perceptions of their decision-makers regarding this behavior. In-depth interviews were conducted with 15 randomly sampled women who chose to deliver at home after receiving antenatal care at a health facility, as well as 15 family members who were purposively identified as decision-makers by the women themselves. Thematic analysis was performed to explore the perceptions, myths, and cultural beliefs about homebirths as well as women’s decision-making power related to childbirth. The three main themes identified showed that traditional beliefs and practices, poverty and gender inequality, and poor healthcare systems significantly influence the preference for childbirth. Traditional beliefs and practices, including religious and cultural beliefs, played a role in perceiving childbirth as a natural process best managed at home. The presence of traditional birth attendants who provide personalized care and emotional support further reinforced this preference. Gender inequalities, including limited access to mobile phones and women’s caregiving roles, were identified as barriers to seeking formal healthcare at the time of delivery. Additionally, poor experiences with the formal healthcare system, such as the poor attitude of formal healthcare workers and fear of medical interventions, also contributed to the decision to deliver at home. The study highlighted the complex interplay between traditional/religious beliefs, gender inequalities, and healthcare experiences in shaping the decision to deliver at home despite receiving ANC services in marginalized settings. Addressing these factors is necessary for promoting facility-based delivery and improving maternal and neonatal outcomes in LMICs.

## Introduction

Sustainable Development Goal 3 (SDG-3) emphasizes the importance of maternal, newborn, and child survival, setting a specific target to reduce the global mortality ratio to 70 deaths per 100,000 live births by 2030 [1]. This agenda has driven concerted efforts in low- and middle-income countries (LMICs) like Pakistan to improve maternal, newborn, and child health (MNCH) through recognized strategies such as antenatal care and skilled birth attendance [2]. It is reported that a higher proportion of women in LMICs now attend at least one antenatal care visit during their pregnancy [3].

Despite these developments, however, the prevalence of home births persists at 28%, with an even higher rate of 33% in South Asia [4]. The Pakistan Demographic and Health Survey corroborates these findings, revealing that 34% of Pakistani women deliver at home with the assistance of traditional or unskilled birth attendants [5]. This poses substantial threats to women’s health in LMICs where poverty, unhygienic living conditions, and unsafe delivery practices increase women’s risk of pregnancy-related complications [6]. The absence of skilled attendance for pregnancy and childbirth hinders the timely identification and management of maternal and fetal morbidities, contributing to adverse pregnancy outcomes [6]. Presently, Pakistan ranks among the countries with alarmingly high levels of maternal morbidity and mortality [7], underscoring the urgent need to understand the factors compelling disadvantaged women to choose home births over health facilities with trained healthcare workers and essential medical supplies.

The continued prevalence of home births points to a complex interplay of sociocultural, political, economic, and ideological factors that shape women’s choice of birthing location. Several studies have examined women’s preferences, choices, and decisions in relation to maternal health care in LMICs, highlighting the impact of geographic location, income, and education levels [8]. Women from impoverished, uneducated backgrounds in rural areas typically struggle with financial barriers [9], long distances from health facilities [10], transportation issues [11], and a lack of health education and awareness [12], which increases their likelihood of delivering at home.

The dynamics of household decision-making have also been identified as an important determinant of the utilization of maternal health care services, particularly in societies where women’s involvement in decision-making is undermined by a traditionally patriarchal family structure [13]. Joint family households are the dominant structure in South Asian countries and family members play a significant role in health decision-making [13]. Similarly, research by Mumtaz and Salway in Pakistan revealed that elderly women, such as mothers-in-law, are considered wise and experienced, and are therefore entrusted with the authority to make decisions related to pregnancy [14]. Even where mothers-in-law become less influential as dynamics within the husband-wife relationship evolve, the quality of their relationship with their daughter-in-law remains a significant influence on women’s uptake of antenatal care [14,15].

This study aims to contribute to the existing literature by qualitatively exploring personal experiences, cultural beliefs, and relationships, driving women to deliver at home despite having access to antenatal care. It further expands its focus to explore the role of husbands, mothers-in-law, and other family members as primary decision-makers to better understand the relational dynamics that influence pregnant women’s decision to deliver at home. By addressing these questions, it aims to deepen our understanding of the complex interplay of factors that shape maternal health-seeking behavior, thereby laying the groundwork for healthcare practitioners and policymakers to bridge gaps in care through the design of culturally sensitive, patient-centered maternal health care services.

## Methods and materials

### Study design

In order to explore the factors influencing women’s decisions to deliver at home after receiving antenatal care, a qualitative exploratory study based on the philosophy of phenomenology was used. This approach allowed the study team to delve deeply into the lived experience of women and decision-makers and achieve a profound understanding of their decisions and choices regarding maternal health care within a marginalized setting.

### Study setting

**Fig 1:**
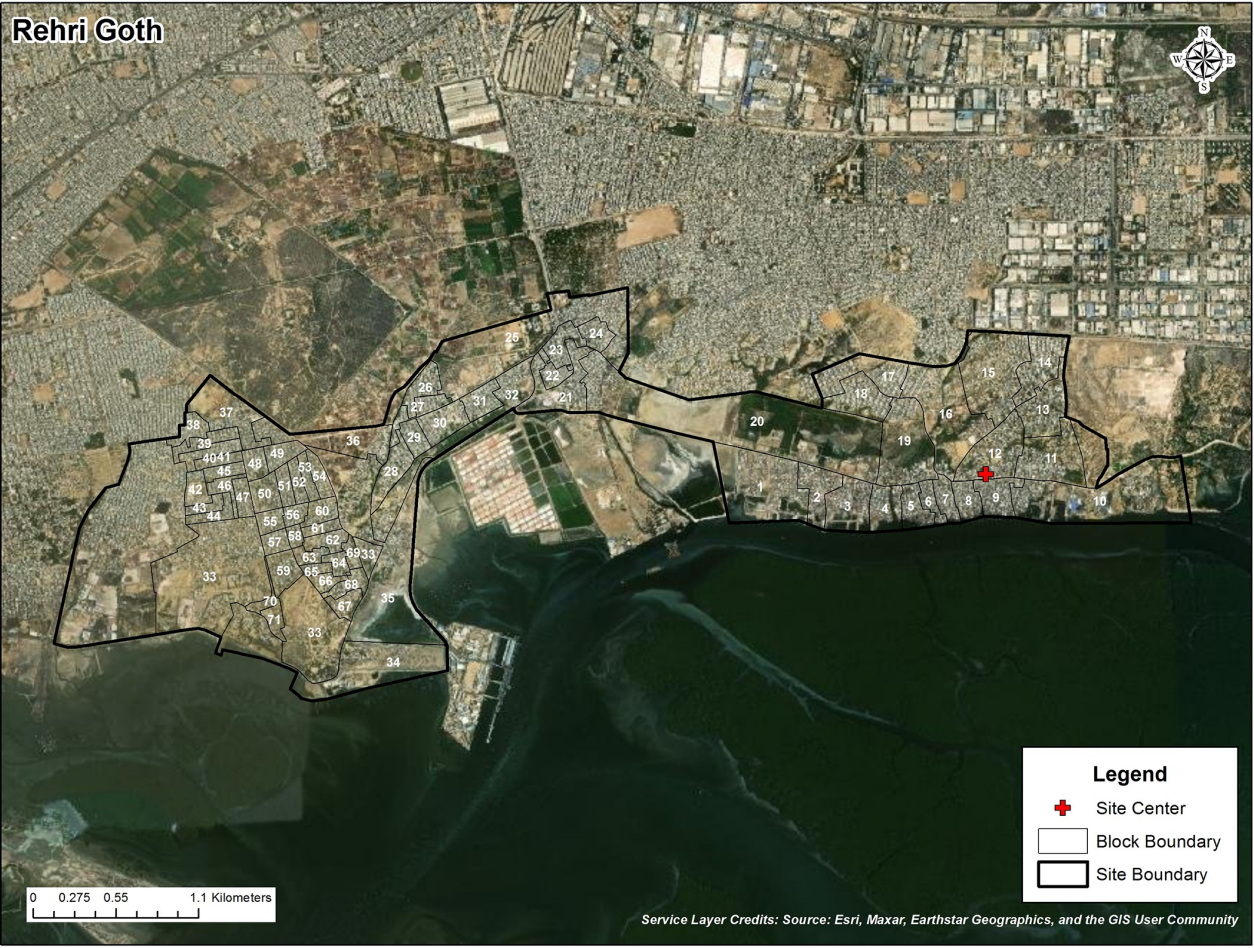
Map depicting block boundaries of Rehri Goth (RG) along with the site clinic and administrative boundary of RG (All layers are the surveyed data sources). The base layer of the map is sourced from the World Imagery map service, which includes imagery from Esri, Maxar, Earthstar Geographics, and contributions from the GIS User Community [16]. The World Imagery map is released under a license compatible with the Creative Commons Attribution 4.0 International (CC-BY 4.0) license [17]. Please refer to the provided links for the terms of use and more information about the base layer.

This study took place between September 2022 and December 2022 in Rehri Goth, a peri-urban fisherman community on the outskirts of Karachi, Pakistan. The community has a population of approximately 112,500 people, with 40% living below the poverty line (refer to Fig 1). The closest public hospital offering labor and delivery services is approximately 25km away. The Department of Pediatrics and Child Health at Aga Khan University (AKU) operates a primary health center (PHC) in this area, where trained community midwives provide antenatal care services. Women are also assisted in referrals to private or public health facilities during childbirth. Routine information about women’s antenatal care, delivery, and postnatal care is captured electronically using an Android-based application and is maintained securely on a cloud database.

### Study participants

In order to identify potential participants, the study team extracted a list of women who had registered for ANC services at the PHC but had delivered at home within the past 6 months from the database. Employing a random sampling strategy to minimize selection bias, the team selected 30 women for the study. For a comprehensive understanding of household decision-making patterns concerning pregnancy and childbirth, the study team purposively selected family members who had been identified by women as primary decision-makers for their health. Notably, in-laws typically assume the role of decision-makers in this community as most men are away for extended periods of time due to their work as fishermen.

Community health workers (CHWs) were given a randomized list of eligible women for conducting initial household visits. They explained the study’s objectives to women and, if women expressed interest, scheduled interviews with the research team. Researchers obtained informed consent from participants before commencing interviews. This approach aimed to ensure that the participant’s convenience was respected throughout the research process. CHWs reached out to all participants a day before their scheduled interviews to confirm their availability. In instances of unavailability or refusal by either the woman or the decision-maker, the next woman on the list was approached until the desired sample size was achieved. Four participants (2 women and 2 decision-makers) withdrew from the study prior to the completion of data collection due to either unavailability or reluctance to answer interview questions.

Originally, the study team aimed to conduct in-depth interviews with 20 households, consisting of both women and their decision-makers. After reviewing field notes, interview transcripts, and audio recordings, the team observed that the information was becoming repetitive, indicating that saturation was reached, and hence concluded interviews after the 15th household.

### Data collection

Data for this study was collected through semi-structured in-depth interviews conducted by KA, HA, and SN in the Urdu language at each participant’s home. Separate interview guides were developed for women and decision-makers after an extensive review of the existing literature on women’s birth place preferences, choices, and decision-making in LMICs. These were pilot tested before finalizing content and structure. During the interviews, women were asked about their preferences for birth location, perceptions about home and facility-based births, birth preparedness, awareness of risk factors and pregnancy complications, and antenatal care experiences. Decision-makers were questioned about their knowledge of maternal health care, birth preparedness, and their opinions on women’s decision-making power in relation to their health.

Each interview lasted between 30 to 45 minutes. To ensure rigor, 2 researchers were present at the time of each interview, with one conducting the interview and the other taking field notes. Female researchers (KA, HA) conducted interviews with women and female decision-makers, while male researchers (SN) engaged male decision-makers. Women and decision-makers were interviewed separately to protect confidentiality and create a safe space for women to share detailed accounts of their perceptions and experiences.

Interviews were conducted by researchers proficient in Urdu, with a translator available in case of language barriers. Participants provided consent for audio recordings, which were later transcribed verbatim in Romanized Urdu to ensure that the transcripts retained their original meaning. Selected quotes were translated into English for inclusion in the manuscript.

### Data analysis

Interview transcripts, along with field notes and observations, were thematically analyzed by AK, KA, HA, and SN using a combination of deductive and inductive coding. All transcripts were coded manually on MS Word. For the initial reduction of data, transcripts were coded using a pre-defined set of codes aligned with the research questions mentioned earlier, including enablers of home births, barriers to facility-based births, and perceptions of pregnancy and childbirth. As the analysis evolved, the team continued to iterate and inductively came up with new codes to review—and re-review—the transcripts against these. After thorough cross-checking, a final set of codes was formulated with relevant data extracts, which were organized into broader themes using MS Excel. Transcripts were reviewed multiple times by the 4 authors (AK, KA, HA and SN) to ensure qualitative rigor. Confusions and disagreements about the codes were resolved collaboratively. Debriefing sessions were held after each round of coding to ensure coding accuracy and enhance the study’s credibility. Senior authors (AK and ZH) reviewed these themes for accuracy.

### Ethical considerations

The study was formally approved by the Ethics Review Committee at Aga Khan University (ERC # 2022-7780-226688). Given the low level of literacy in the community, all participants were verbally informed about the nature and goals of the study, and voluntary participation was emphasized. Participants with basic literacy were required to sign an informed consent form, while those who were unable to read or write were counseled in their first language with the help of community health workers and were required to provide their thumb impressions as a means of consent. Steps were taken throughout the research process to protect participant privacy and confidentiality. All personally identifiable information was pseudonymized and participants were assured that no personal identifiers would be included in any publications to come out of the study.

No monetary compensation was provided to participants; however, the potential benefits of participation at the community level, such as improved cultural competence in health care, were clearly communicated. Participants were also regularly reminded that their answers to questions or refusal to participate at any point in the study would not compromise the services they receive at the PHC in any way.

### Positionality statement

As this was a qualitative exploratory study, the researchers thoroughly considered issues of identity, positionality, and reflexivity. The research team included five researchers with varying levels of experience in maternal and child health research. The interviews were conducted by early-career researchers who had backgrounds in social science and in medicine (KA, HA and SN). Throughout the research process, they received support from AK and ZH, experienced researchers with expertise in public health, to ensure that the study was conducted rigorously and ethically.

Researcher positioning and its implications for the research process are often explained in terms of the researcher being an “insider” or an “outsider” to the population being studied [18]. Although the study population belonged to the city in which the researchers resided, their material privilege, resulting from their class position, placed them at a distance from the experiences of the marginalized and vulnerable population they were writing about. Their knowledge, awareness, and attitudes regarding the subject of interest stemmed primarily from their professional work rather than personal lives. Here, it is important to acknowledge how the research team’s affiliation with Aga Khan University and, by extension, the PHC, affected the research process. On the one hand, researchers were able to negotiate access with relative ease due to the familiarity and trust that come with an established presence in the community. However, due to the inherent differences in social, economic, and occupational status, it was important to contain the researchers’ largely biomedical understanding of these events to create space for participants to freely share their experiences. Throughout the interviews, participants were recognized as experts in their own lives and probed to share their thoughts and feelings about the issue of interest.

## Results

### Participant profile

The demographic information of the 15 women and their decisionmakers included in this study has been provided in Table 1. The women’s ages ranged from 18 to 38 years, 66.67% (n = 10) had no formal education and all of them were unemployed. The majority of the women in this study (74%, n = 11) had four or more ANC visits during their last pregnancy. The decision-makers’ ages ranged from 22 to 76 years, had no formal education (86.7%, n = 13), and were unemployed (66.7%, n = 10). Nearly half of the decision-makers were parents-in-law (46.7%, n = 7) followed by parents (20%, n = 3) and other extended family members including paternal and maternal aunts and sisters-in-law.

**Table 1:** Baseline characteristics of the women and decision-makers enrolled in the study.

Women	Total; n = 15 n (%)
**Age, years; median (range)**	25 (18–38)
**Education**	
No formal education	10 (66.67)
**Occupation**	
Unemployed	15 (100)
**Parity; median (range)**	3 (1–8)
Primiparous	3 (20)
Multiparous	9 (60)
Grand Multiparous	3 (20)
**ANC visits**	
1–3 visits	4 (26.67)
≥4 visits	11 (73.33)
**Decision-makers**	**Total; n = 15** **n (%)**
**Age, years; median (range)**	50 (30–75)
**Gender**	
Male	3 (20)
Female	12 (80)
**Relation to woman**	
Husband	2 (13.33)
Parent	3 (20)
Parent-in-law	7 (46.67)
Other extended family	3 (20)
**Education**	
No formal education	13 (86.67)
**Occupation**	
Unemployed	10 (66.67)

**Fig 2:**
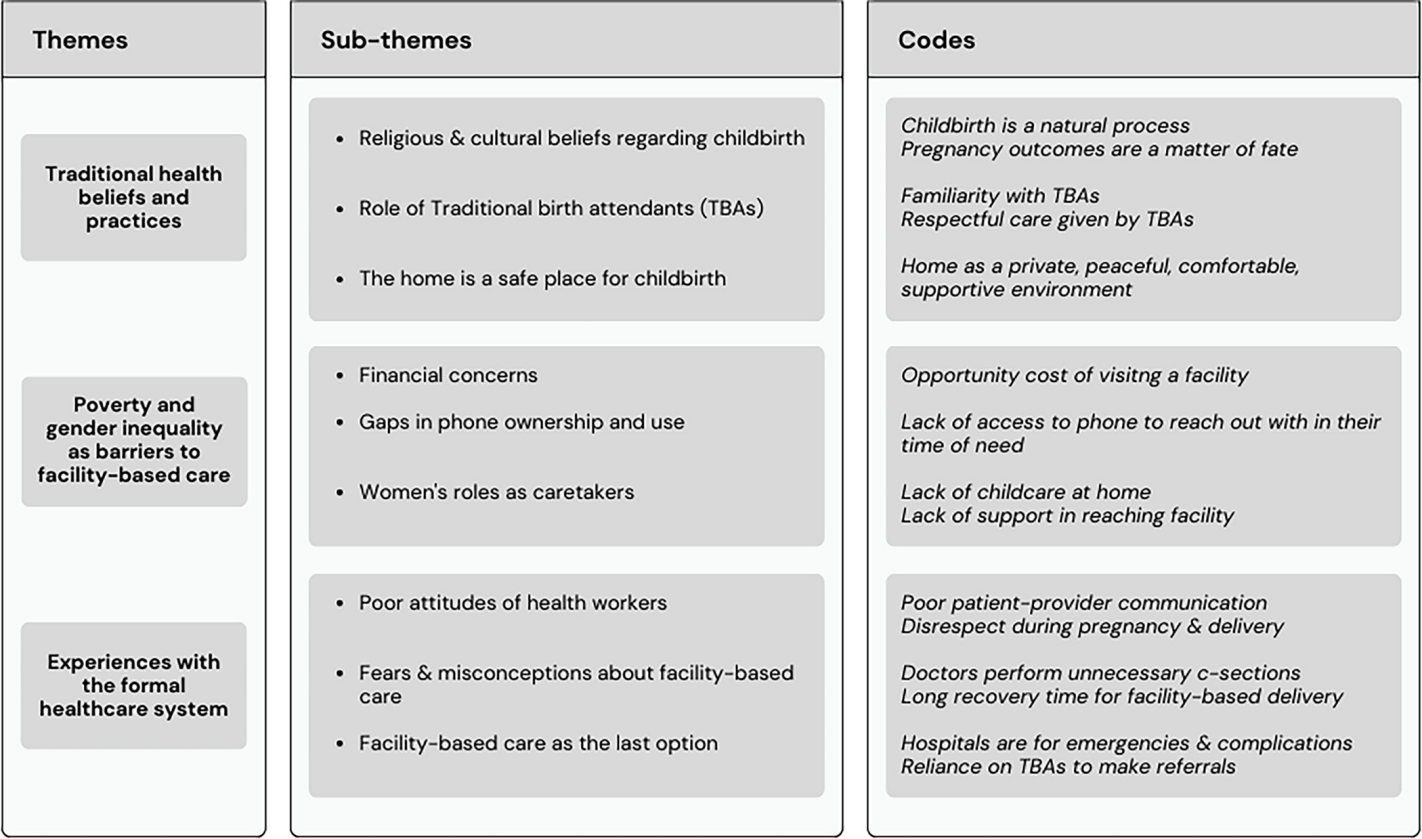
Study themes, their respective subthemes, and codes.

Thematic analysis revealed three major themes, broadly encompassing why women chose to deliver at home despite the availability of free maternity care (refer to Fig 2). These included (i) traditional health beliefs and practices, (ii) poverty and gender inequalities as a barrier to formal healthcare, and (iii) experiences with the formal healthcare system.

Themes were informed by the perspectives of both women and decision-makers. The study team found that the majority of women and decision-makers shared similar opinions regarding the benefits of home births, which were shaped by long-standing health beliefs and practices within the community. Women typically made decisions about the birth place in consultation with family members, especially female elders such as mothers and mothers-in-law. Drawing upon their personal experiences with childbirth and broader social networks, which included their ties with TBAs, female decision-makers assessed different modalities of birth and provided women with recommendations on how to prepare for it. Conversely, male decision-makers typically regarded pregnancy and childbirth as matters exclusively concerning women and deferred the ultimate decision to the pregnant women themselves. The key factors that influenced women’s decision-making are detailed below.

## Traditional health beliefs and practices

### Religious and cultural beliefs regarding childbirth

Several women and decision-makers described pregnancy and childbirth as “normal” life events that could be experienced without the need for medical intervention. A history of successful home births in their families further reinforced the view that pregnancy was a “natural” transition for women that could, in most cases, be safely managed at home. In contrast, visits to health facilities were associated with “abnormal” circumstances such as underlying medical conditions and pregnancy complications.

"We are villagers, and we feel comfortable at home only. Even my sister delivered her children at home, and all of them are healthy, thanks to God. Women from the city go to the hospital, where they are given injections for pain and induced labor. Nevertheless, we are villagers–we believe that labor should occur naturally." (Decision-maker #14, Mother)

*“I decided not to go to the hospital for delivery because I felt perfectly healthy*. *I wasn’t feeling any pain that would be a cause for me to go*.*”* (Woman #13)

Religious and spiritual beliefs also shaped how women made sense of and managed their pregnancy. Religiously derived fatalism, or the belief that health, illness, and death are expressions of God’s will, is common in religious contexts like Pakistan. Individuals typically turn to religious practices, such as prayer, in an effort to invoke divine intervention for improved health outcomes and as a means of coping with illness, pain, and death. Similar beliefs and practices were observed among the women and decision-makers in this study. Half of the participants explicitly stated that, above all, they left the safety of the pregnancy in the hands of a divine power. The belief that health outcomes, whether positive or negative, are largely a matter of fate and therefore unavoidable, strengthened their preference for long-standing traditions such as home births, as these were associated with a positive birthing experience.

“God decides who lives or dies…you and I cannot save those who are meant to die.” (Decision-maker #1, Mother-in-law)

“The midwife told me that my baby is breech but I had people pray for me…and by the grace of God, I did not face any difficulties during delivery.” (Woman #4)

#### Role of traditional birth attendants

Women and decision-makers further highlighted that their ties with traditional birth attendants (TBAs) encouraged home births. As community members, TBAs deeply understand the traditions, cultures, and languages of the women under their care. This allows them to build strong relationships with both women and their families and, as a result, provide individualized care that is not easily replicable in a hospital setting. In several cases, TBAs were not only neighbors but also direct or extended family members of the women giving birth, thereby strengthening their bond, and fostering trust in home-based deliveries.

"My mother-in-law is a TBA. When she is there—I feel at peace knowing that there is an elder with me. She gives me something to help build my strength too." (Woman #8)

Given their availability and degree of familiarity with women, TBAs were often termed and perceived as a mother figure. The local word used for a TBA was *dai-ma (dai* for TBA*)*, where *ma* means mother. Like mothers, TBAs were believed to provide women with guidance and support during pregnancy, labor, and the postnatal period. Their ability to connect with women socially and emotionally during the labor process by virtue of their background helped women feel supported, listened to, and understood.

“A TBA is just like a mother.” (Woman #2)

Women and female decision-makers also believed that giving birth at home with the assistance of a TBA was more efficient. Facility deliveries were associated with delayed care, and long length of stay and slow recovery. Conversely, women revealed that the combination of methods used by TBAs, including various herbal medicines as well as massage, result in shorter labor and, in most cases, faster and easier recovery.

*“Home births are easier and less painful*. *The TBA makes the mother a [herbal] medicine and she is up and about within 3 days*. *On the other hand*, *hospital births require a 3-day stay and often leave the mother feeling unwell even after returning home*.*”* (Decision-maker #1, Mother-in-law)

#### The home is a safe place for childbirth

Women described the labor process as a challenging and unpredictable experience that called for a "safe" (private and peaceful) space. It was believed that creating such an environment at home was easier than at a hospital. Women reported high satisfaction with giving birth in the comfort of their homes, surrounded by supportive family members who helped reduce their anxiety and stress levels. This was in contrast to hospitals, where they expected to experience discomfort, inconvenience, and a lack of privacy.

“I prefer giving birth at home because my mother is here to support me and no one else is here to watch me.” (Woman #10)

“I’ve never given birth at a hospital. But at home, I feel taken care of. I feel at peace because I can move around freely with the help of my mother, since she can stay with me.” (Woman #12)

### Poverty and gender inequality as barriers to formal healthcare

#### Financial concerns

Financial concerns also influenced decision-making for birth location. Despite the availability of no-cost or low-cost labor and delivery services at referral facilities, participants highlighted associated opportunity costs, such as the loss of earnings for the husband (the family breadwinner) and travel expenses, as barriers to facility-based delivery. There was a notable gender difference in the significance assigned to financial resources when choosing a place of birth. Women and female decision-makers tended to prioritize comfort and convenience over the cost of delivery, preferring to pay 3000 to 4000 PKR for a home birth over delivering at an affordable and well-equipped health facility. Conversely, male decision-makers preferred adjusting the cost of the pregnancy within the overall household budget. Some of them actively promoted antenatal care visits and designated limited funds for nutrition and medicines during pregnancy to reduce the risk of complications and ensure a favorable birth outcome at home, which they regarded as the more cost-effective alternative.

“We are poor, so we do not have many finances to quickly take her in a car, keep her at the hospital, and feed her. That is why we want to spend a little [on ANC] so that she can have a normal delivery at home and be safe here. This way, it comforts me that my wife is safe at home, and I can go and focus on my work.” (Decision-maker 4, #Husband)

#### Gaps in mobile phone access and use

Even when women intended to call and arrange for transportation for facility-based delivery, they frequently faced challenges in accessing a mobile phone in time, resulting in a home birth. Limited financial resources often led to the sharing of a basic handset, with priority given to male family members who required them for employment. This adversely affected pregnant women’s ability to access care in urgent circumstances. In situations where women went into labor while male family members were away for work, contacting a TBA for delivery was found to be a more feasible option.

“I had planned for a facility birth, but at the time of delivery, I had no mobile phone. My husband had it, and he was not home.” (Woman #13)

#### Women’s roles as caretakers

Women also faced barriers related to their roles as caretakers they take primary responsibility for unpaid domestic work while male family members need to work long hours for their livelihoods. This is further compounded by prevailing gender norms underpinning differences in household responsibilities. The significant amount of time required to seek healthcare, particularly maternity care, is believed to interfere with their responsibilities as caretakers. Several women emphasized the challenges they face in making alternative arrangements for housework and childcare for long stays away from home. This led to delaying, if not altogether forgoing, facility-based care, ultimately resulting in the choice of home birth.

“It is just us [women] at home. My husband is only with us for two months a year. I do not feel like leaving my children alone at home, so we prefer to call the TBA over. It makes the whole birthing process easier.” (Woman #12)

### Experiences with the formal healthcare system

#### Poor attitude of formal healthcare workers

Several women identified a lack of soft skills such as empathy and compassion among midwives and doctors as a hindrance to facility-based delivery. Pregnancy and childbirth were seen as major physical and psychological changes, and a lack of empathy and kindness, coupled with a disregard for women’s needs, contributed to an unsettling experience for mothers. Women argued that healthcare providers frequently failed to comprehend their pain and made minimal efforts to guide them through the changes occurring in their bodies, particularly during delivery, which was particularly distressing for first-time mothers.

“When I had my first child, I was completely overwhelmed by uncertainty. The pain was so intense, and I just longed to be back in my own home. I was already nervous about being in a hospital, and to make things worse, I received an injection and a drip with no explanation whatsoever. It was such a strange and unfamiliar experience that left me feeling anxious and unsure of what was to come.” (Woman #10)

Women who visited a formal health facility for the delivery of a previous child or other health-related issues reported that healthcare providers were unapproachable, if not altogether hostile. They recounted instances where healthcare providers would rebuke patients loudly, In contrast, the same women noted that TBAs and family members displayed greater kindness and patience, suggesting that home births may be a better option.

*“I gave birth to my first child at a hospital*, *but no one explained anything to me while I was there*. *This time*, *at home*, *my sister-in-law and the TBA guided me through everything*, *they explained what problems I might face*, *and the whole [birthing] process became easier for me*.*”* (Woman #7)

#### Fears and misconceptions regarding formal healthcare

While highlighting their poor experiences with formal healthcare, women and decision-makers shared their perceptions of the hospital as unwelcoming and unsafe due to a general lack of exposure to and unfamiliarity with hospital environments. Negative experiences of family members, friends, and neighbors further reinforced these perceptions and increased fear and anxiety about hospitalization, contributing to the preference for home births.

“I have heard that the [health workers] are always screaming at the hospital. It’s scary. You are already in excruciating pain and, to make matters worse, people are screaming at you or around you.” (Woman #2)

Others reported apprehensions towards surgical procedures, including unnecessary cesarean sections and discomfort or complications after delivery.

“At government hospitals, if a woman experiences the slightest of pain [at the time of delivery], doctors end up performing surgery. This scares women.” (Decision-maker #13, Husband)

“When you deliver at a hospital, you have an operation. This can make it difficult to look after your other children as you have stitches that can open…you can face lots of problems.” (Woman #2)

#### Facility-based care as the last option

Considering their anxiety and preference for home-based care, women and decision-makers believed hospital deliveries are only necessary during complications and emergencies. For this, they would rely on the TBA’s judgment; if the TBAs felt that their pregnancy was high-risk and complications were likely, women would opt for a facility-based delivery.

“I go to a TBA first. She assesses my pregnancy and advises me about the place of birth accordingly…she lets me know if I can deliver at home. If it is not possible, she tells me to go to the hospital.” (Woman #4)

## Discussion

The current study reports that traditional/religious beliefs and practices, gender inequality, and poor healthcare systems have a significant influence on the health care seeking behavior at the time of childbirth for many women and their families living in marginalized settings. Although care is accessible and free of cost, several factors at the community level, including existing traditions and beliefs, as well as inadequate and substandard care at health facilities, often deter women from seeking necessary medical care during childbirth that could potentially save their lives.

Many women and decision-makers’ preference for home births stemmed from pre-existing religious and cultural beliefs. For most, it was customary to deliver at home. They supplemented their interest in continuing this long-standing tradition by stating that the pregnancy outcome depends on a ‘higher power’. This finding resonates with previous literature where women and their family members emphasize the importance of religious and cultural factors for their birthing practices and pregnancy outcomes [19]. In keeping with cultural norms, the women and decision-makers also prioritized using TBAs for home births due to their deep personal ties with them. This is similar to a study from Bangladesh which reported that the TBA is considered a respectful community member due to their cultural consciousness, trustworthiness, and close relationship with the community, thus playing a significant role in the preference for home births [11].

Women in our study stressed the environment of care as a concern when choosing a place of birth. The home was viewed as a safe place for birth where women’s needs and preferences were better understood and supported. Our findings are in concordance with previous literature that suggests that women value a supportive and familiar environment during childbirth. A recent study in Somaliland has shown that home births are believed to make it easier to manage women’s concerns about privacy and psychosocial support during childbirth [20]. Similarly, an interpretive synthesis of literature relating the concepts of "place" and "space" to childbirth suggests that providing women with a familiar and home-like environment can increase feelings of comfort, control, and autonomy during labor and delivery, which plays a significant role in shaping positive experiences with childbirth [21].

While there is little evidence directly linking mobile phone ownership with improvements in maternal and newborn outcomes, there is recognition that mobile phones can make it easier to interact with the formal healthcare system. LeFevre et al. analyzed Demographic Health Survey (DHS) data on phone ownership for 15 countries and found that women who own a mobile phone were more likely (1.25 OR, 95% Cl: 1.1–1.35) to deliver with a skilled birth attendant [22]. Several women in our study reported that they gave birth at home because they could not access a mobile phone to request transport for delivery.

Additionally, gender norms that position women as caretakers were associated with high workloads during pregnancy and after childbirth. Women in our study reported choosing their homes for birth because they were responsible for household chores and caring for children. Yaya et al. found that pregnant women in rural Nigeria were expected to perform daily chores with little to no assistance from their husbands leaving them with insufficient time and energy to seek care [23]. The unequal distribution of unpaid care and domestic work has brought attention to women’s “time poverty” and its impact on their health and well-being [24]. A significant care burden that limits women’s discretionary time ultimately promotes a culture of self-neglect, hindering routine and emergency care utilization, even when pregnant [25].

While the decision-makers in this study expressed support for women’s birthing location choices, it is crucial to consider the socio-cultural norms prevailing in Pakistan, where women often have limited power, particularly in the realms of decision-making [26]. In Pakistan and other South Asian countries, decision-making is predominantly influenced by elders, such as parents, parents-in-law, and husbands, resulting in a diminished role for women in decision-making processes [27]. A study conducted by Yazdani et al. within the same community as our study found that only 2% of women made independent decisions regarding seeking care, while 98% relied on others to make decisions with or for them [28]. Considering this power imbalance, it is plausible that the women in our study were influenced by the traditions and opinions of their husbands and elders while opting for home-based deliveries.

The experiences with formal healthcare play a vital role in the decision of choosing the birthing location. The results showed that lack of trust and confidence in formal healthcare results from several factors, including the poor attitudes of healthcare professionals, inadequate healthcare facilities, communication problems, and a lack of involvement in the birthing process. Lack of dignified care, maltreatment, and unfriendly attitude are reported as significant barriers to skilled deliveries [29]. Additionally, insufficient knowledge and awareness about the advantages of facility base delivery, limited information on complications during labor and delivery, mishandling, and inadequate health care resources exacerbate existing fears among the low-resource communities when it comes to choosing health facilities for childbirth [30].

One notable limitation of our study is that the majority of the participants had no formal education which may have influenced participants’ responses. While we aimed for a diverse sample, the small representation of male decision-makers may not fully capture the perspectives of men in the population. This limitation arises from cultural norms and may reflect the challenge of engaging male family members in discussions related to maternal and child healthcare in certain communities. Additionally, the study findings are from a specific area of Pakistan and thus may not be generalizable to other areas or countries with different socio-cultural contexts.

Further research is needed to support the design of culturally sensitive interventions and improve existing health service delivery models in ways that prioritize the interests of marginalized women. Findings from this study revealed that childbirth is perceived as the woman’s responsibility, rather than a shared responsibility between partners. There is a need to understand the role of men during pregnancy and identify more meaningful ways to engage male partners through group counselling and family involvement interventions. The study also highlights the necessity of not only training formal health workers in respectful maternity care but also recognizing the health system challenges that prevent them from providing it in the first place to improve women’s childbirth experience at facilities.

## Conclusion

In conclusion, a preference for home births is a manifestation of conventional traditional health beliefs and practices, prevailing gender inequalities in the accessibility of health care, as well as dissatisfaction with formal health care services. In order to deal with this complex issue, it is necessary to work on behavioral change strategies for healthcare seeking as well as significantly improving the accessibility and quality of formal healthcare services. Collaboration between healthcare professionals, policymakers, and community members is important to ensure positive health outcomes for women and newborns in these communities.

## Supporting information

S1 Data This contain data from all in-depth interviews. (PDF)

S2 Data This contain data from all in-depth interviews. (PDF)
