# Supplementary material for: Why do women still give birth at home; perceptions of Pakistani women and decision-makers from marginalized communities
Source: PLOS Glob Public Health. 2023 Oct 13;3(10):e0002217. doi: 10.1371/journal.pgph.0002217 (PMC10575520; doi:10.1371/journal.pgph.0002217)
Supplement: S1 Data — (PDF) [file pgph.0002217.s001.pdf]

IDI WOMAN-01

Interviewer: Pehle main aap se poochna chahungi k apki umar kitni hai

Woman-01: 21 saal

Interviewer: Apki shaadi ko kitna time hogya hai

Woman-01: 5 saal

Interviewer: Apke abhi kitne bachey hain

Woman-01: 2

Interviewer: Acha ye apki dusri beti hai

Woman-01: Ji

Interviewer: Apkey pichley jitney hamal the woh ap ne ghar par kiye the ya kisi hospital main ja kar

Woman-01: ghar main hi kiye the dono

Interviewer: Ap ne taleem kitni hasil ki hai

Woman-01: taleem nahi ki hai kuch bhi

Interviewer: aur ap kuch kaam karti hain?

Woman-01: han company mai karti thi ab nahi karti

Interviewer: Acha sab se pehle ap hamein bata sakti hain k bachey ki pedaaish k liye behtareen jagha konsi hoti hai?

Woman-01: Ghar main

Interviewer: ap bata sakti hain k ghar main paida karney k fawaaid kya hote hain?

Woman-01: Ghar main asani hoti hai

Interviewer: asani k elawa or kya acha lagta hai apko ghar par

Woman-01: waise hospital bhi sahi hai lakin ghar main ziada sahi hota hai

Interviewer: ghar par asani kis lehaz se hoti hai?

Woman-01: asani jaise maasi hai na meri mere pass hoti hai (sab pas hote hain isliye) asani hoti hai

Interviewer: kya ap mujhe bata sakti hain jaise ap ne kaha k ghar par bohat asani hoti hai or apko acha lagta hai lekin kya apko lagta hai k kuch nuqsan bhi ho saktey hain ghar par paida karney ke?

Woman-01: nuqsan nahi ho saktey.

Interviewer: acha bachey ki ghar main pedaaish se waabasta kuch khas soch hai apki?

Woman-01: nahi

Interviewer: acha ghar main paida karna apka apna faisla hota hai?

Woman-01: J haan

Interviewer: ap k khayal main aise konse aise halaat hote hain jin main hospital main delivery karwani parti hai?

Woman-01: nahi aisa nahi hota ghar main hojata hai

Interviewer: ye bataein jo hamal abhi apka guzra hai us k darmiyan ap center par aa rahi thin us k baawajud ap ne delivery ghar par kyun ki?

Woman-01: maine gor kiya k wahan mujhey bola unhon ne k apko saatwan mahina chal raha hai jab k mujhe pure mahine the, main ghar par gaen hun toh wahan bacha paida hogaya 2-minute bhi nahi lagey.

Interviewer: kya unhon ne (center main) apko bataya tha k ap hospital bhi ja sakti hain?

Woman-01: nahi us ne kuch nahi kaha, kaha k time abhi kam hai jab k time pura tha phir main dubara nahi gayi center.

Interviewer: joh center main health workers hoti hain ap ko mashwara dene k liye kya wo apko sahi lagti hain?

Woman-01: han woh sahi lagti hain.

Interviewer: aur jis tarhan health workers apko samajhti hain ap se jis tarhan baat karti hain apko kaisa lagta hai?

Woman-01: woh sahi lagta hai

Interviewer: toh kya apko center main sab kuch theek or sahi lag raha tha? or kya cheez sab se ziada achi lag rahi thi?

Woman-01: wahan ki dawaiyaan achi lagti thi

Interviewer: aur wahan ki koi aesi cheez joh apko achi nahi lagi ya koi aesi baat joh ap jaise chah rahi thi waise na huyi ho?

Woman-01: mujhe khoon ki bottles nahi lagaai.

Interviewer: apko is cheez se koi asar para tha k mujhe ghar main hi karana hai or hospital main nahi karna?

Woman-01: goliya dawaiyaan khati thi to mujhe lagta tha k ghar main hojaaye.

Interviewer: acha jaise ap ne bataya k pehle wala bacha bhi apka ghar par hi hua hai to ghar par delivery karne ka apka tajaruba kaisa raha?

Woman-01: sab cheezen asani se hojati hain

Interviewer: acha to is se apki sehat par koi asar ya nuqsan nahi hua is se?

Woman-01: nahi

Interviewer: aur bachey sahi hain ap k sehat mand hain?

Woman-01: haan sehat mand hain.

Interviewer: aur ap abhi ja rahi hain center par?

Woman-01: nahi ja rahi

Interviewer: bachon k liye bhi nahi ja rahi?

Woman-01: nahi un k liye b nahi ja rahi

Interviewer: ap k ghar main ziada tar faisley kon karta hai?

Woman-01: maasi (DM-01).

Interviewer: toh bachon ki delivery k liye jagah se mutaliq un k or ap k khayalaat ek hi hain ya mukhtalif hain?

Woman-01: ek hi hain

Interviewer: kya ap bata sakti hain k ghar par sab se ziada bharosa ap kis par karti hain?

Woman-01: maasi par.

Interviewer: jab ap ghar par hoti hain toh kya yeh apki madad karti hain?

Woman-01: han bohat madad karti hain koi bhi nahi karta lekin yeh karti hain (maasi).

Interviewer: aur jab apki delivery ho rahi thi us k liye kon aya tha?

Woman-01: ye aayi thi (maasi)

Interviewer: aur pehle bachey k waqt bhi aisa hi tha?

Woman-01: haan

Interviewer: jab apka bacha paida hua tha to us waqt center aaen thi kisi bhi hawaley se ya checkup karwane?

Woman-01: nahi aayi thi

Interviewer: kya apko center ki tamaam sahooliyaat asani se mil jati hain? koi jaaney ka masla door ka masla?

Woman-01: nahi koi masla nahi.

Interviewer: kya chal kar jaati hain ap? koi masla hota tha?

Woman-01: Ji chal kar jaati hun koi masla nahi hota.

Interviewer: aur jaati kis k saath hain ap? ya akele jaati hain?

Woman-01: nahi is k sath jaati hun (maasi) ya kabhi dost k sath.

Interviewer: joh ap ilaaj karwa rahi hain us main paise bohat lag rahe hain ap k ya bas munasib?

Woman-01: nahi paise nahi lag rahe bas paanch hazaar lagey the.

Interviewer: aur dawaiyon k liye bhi paise nahi kharch karney parte?

Woman-01: Ji nahi

Interviewer: acha ab main poochna chah rahi thi k or koi aisi cheez hai jis ki waja se apko hospital main bachey karney main rukawat ho rahi hai?

Woman-01: nahi main khud nahi aati

Interviewer: acha koi or cheez hai joh ap batana chahein ghar main pedaaish k mutaaliq?

Woman-01: Ji nahi ……

___________________________X_____________________________

IDI WOMAN-02

Interviewer: aur aap ki shadi kitne arsey se hai

Woman-02: meri shadi ko 2 saal hogaye hain

Interviewer: Aap k kitney bache hain

Woman-02: ek

Interviewer: ek sahi hai aur ap k ghar pe hi huyi hai yeh bache aur ap ne taaleem kitni haasil ki hai

Woman-02: nahi ki

Interviewer: sahi hai ap kahin kaam karti hain ya ghar pe

Woman-02: nahi ghar pe hi hoti hun

Interviewer: ghar pe hi sahi acha sab se pehle ap se mujhe yeh poochna hai k ap ko sab se behtareen jagha konsi achi lagti hai bache paida karne k liye

Woman-02: ghar main sahi hai

Interviewer: ghar mein sahi hai ..ap bata sakti hain k kyun

Woman-02: hispatal mein toh ziadatar operation hi hota hai

Interviewer: hmm

Woman-02: woh sahi nahi hai

Interviewer: hmm hmm hmm

Woman-02: waise normal joh hoti hai na woh sahi hai

Interviewer: ap ki delivery normal thi ya

Woman-02: nahi normal thi

Interviewer: normal thi sahi hai aur koi misaal de sakti hain kyun ghar pe behtar lagta hai ap ko

Woman-02: kyun k hispatal mein jab delivery hoti hai operation se toh woh dusre bache ki baat horahi hai joh taanke lagte hain dusre bache pe toot bhi sakte hain toh bohat saare masle hosakte hain

Interviewer: hmm

Woman-02: isliye normal delivery sahi hai

Interviewer: hmm aur ghar pe aap ko kia acha lagta hai matlab jaise ghar pe ap paida kar rahi hain toh ghar k barey main kia acha lagta hai bacha paida karne k liye

Woman-02: han is k bohat saare faide hote hain

Interviewer: misaal k tor par

Woman-02: kyun k ilaaj matlab acha hota hai

Interviewer: thek hai

Woman-02: hispatal mein toh sirf dawaai wagera yehi hote hain aur yahan pe joh hamein sahoolat mila pata nahi kia kia woh sahi hota hai

Interviewer: jee

Woman-02: khoon bhi sahi tarhan se aata hai

Interviewer: jee jee jee jee

Woman-02: toh aese ghar mein delivery bohat achi hoti hai

Interviewer: aap kisi ko phone kar k bulaate hain bacha paida karne ka jab waqt ajaata hai

Woman-02: nahi hum khud chale jaate hain bache paida karwaate hain aurtein

Interviewer: jee joh TBA hoti hai

Woman-02: han

Interviewer: thek hai sahi toh un k ghar chale jaate hain

Woman-02: han

Interviewer: thek hai

Woman-02: ya toh un ko bula leite hain

Interviewer: thek thek thek acha toh sahi hai ab mein poochna chah rahi hun k ap ko koi ghar pe paida karne se ap ko lagta hai k koi nuqsaan bhi hosakta hai

Woman-02: han aisa lagta hai

Interviewer: kia kia nuqsaan hote hain misaal k tor par

Woman-02: matlab jaise k khoon ziada hojaata hai un ko band karna mushkil hojaata hai bacha ulta bhi hosakta hai tairah bhi hosakta hai aisa bhi dar hota hai

Interviewer: jee bilkul ap kisi ko jaanti hain jin k saath yeh aisa hua hai kabhi

Woman-02: han ek larki ko jaanti hun us ki delivery normal hone waali thi lekin achanak se bacha ulta hogaya usay hispatal jaana para operation hua us ka

Interviewer: sahi hai acha toh us se bhi ap ko kauf ya kuch hota hai k ghar pe karna chaiye hispatal mein karna chaiye is se mutaaliq koi

Woman-02: yeh sawal nahi samjh aaya

Interviewer: k matlab jab ap sunein k dusron k saath kia horaha hai ap ko lagata hai k ghar pe hi sahi hai ya hispatal pe kia hona chaiye

Woman-02: haan jab koi masla hota hai toh aisa lagta hai k ab hispatal hi jana chaiye

Interviewer: hmm acha toh ap bata sakti hain k kaise kaise maslon mein phir jana parta hai hispatal delivery k time pe

Woman-02: matlab khoon bohat aata hai bacha aane se pehle

Interviewer: sahi

Woman-02: aur bacha ulta bhi hojaata hai tairah bhi hojaata hai ya toh who phas bhi jaata hai

Interviewer: sahi sahi thek hai aur ap k aise koi khayalaat hain joh baron se sunti arahi hain k ghar pe paida karna isliye isliye acha hai aise koi pedaaish se waabasta koi khaas soch hai ap k ghar mein ya kahin aur jaise ap k bare baat kar rahe hon k unhon ne bhi ghar pe a k kiya ho ap logon ko phir bata rahe hon k yeh isliye acha hai toh kis tarhan ki baatein hain joh bataatey hain ap ko

Woman-02: matlab aise k bari bari aurtein hain woh bolti hain ghar mein joh normal delivery hai woh sahi hai

Interviewer: hmm

Woman-02: hispatal mein toh sahi nahi hai

Interviewer: hmm acha woh bataatey hain k kyun sahi nahi hai

Woman-02: matlab isliye sahi nahi hai k hispatal k bohat saare masle hote hain jaise k mein ne pehle bhi ap ko bataya operation

Interviewer: hmm hmm hmm

Woman-02: aise yeh batati hain k ghar pe hi delivery sahi hai

Interviewer: jee toh ap ka faisla khud tha k idhar ap ko ghar pe karna hai? Ya jis ne ap ko samjhaaya tha?

Woman-02: mera khud ka faisla tha k ghar pe karon

Interviewer: thek thek thek acha toh acha ab mein poochna chah rahi hun k abhi joh ap ka joh abhi hamal guzra hai toh us mein ap is center pe arahi thin hamare center pe aur us ka ap k sath kis tarhan ka tajarba raha

Woman-02: matlab ilaaj

Interviewer: han ap ko kaisa lag raha tha

Woman-02: sahi tha bilkul

Interviewer: acha toh ap ki counselling wagera sab huyi thi ya ap se woh baat karte thy?

Woman-02: nahi baat cheet toh nahi hoti thi kia ultrasound hai is ka kia hoga dawaai deite thy k nahi

Interviewer: thek hai sahi sahi acha toh us se koi asar para tha Jahan pe ap paida karna chati hain k jis tarhan se woh tha ap k sath sulook kaisa tha , sulook jis tarhan se tha ap k sath , ap k sath sahi tarhan se pesh arahe thy

Woman-02: ap center wale ki baat kar rahi hain?

Interviewer: han center wale ki baat kar rahi hun

Woman-02: wahan sahi thy

Interviewer: aur ap har visit pe jaati thin matlab ap ko jab bhi bulaaya jaata tha tab jaati thin ya kabhi kam bhi jaati thin

Woman-02: jab taareekh aati thi tab jaate thy

Interviewer: jab taareekh aati thi thek hai acha toh yeh mein poochna chah rahi hun k ap ko koi hamare woh center pe koi cheez achi shayad nahi lagi ho aisi koi baat hai udhar ki joh ap chahti hon k is mein behari ho magar kuch cheez sahi nahi thi

Woman-02: sab sahi tha

Interviewer: sab sahi hai jaise koi baat karne ka tareeka sahi se ap ko samjhaate hain daanttey hain aise koi cheez hai hum bas janna chah rahe hain hum joh apni khidmaat de rahe hain us ko behtar karna chahte hain is liye hum poochna chahte hain ta k behtar karein toh koi bhi agar ap ko lag raha tha k udhar koi masla tha koi cheez behtar ho sakti hai

Woman-02: nahi sab kuch sahi tha

Interviewer: acha aur kisi ko jaante hain jin ko tajaruba acha nahi gaya center pe

Woman-02: nahi

Interviewer: nahi .. sahi toh acha ab mein poochna chah rahi hun k hamesha se ap ka irada tha k ghar pe hi karna tha ya pehle ap ka khayal tha k shayad mein hospital mein kar leiti

Woman-02: mera toh aise koi khayal nahi tha

Interviewer: acha jab time aaya toh faisla pehle se tha k ghar pe hi karna hai

Woman-02: han

Interviewer: acha toh kisi ghar k bare me ap ko samjhaya is k baare mein

Woman-02: hispatal k maslon se mein darti thi ghar k maslon se bhi darti thi pehla bacha tha na kaise kaise khayal aate thy

Interviewer: hmmmm hmm .. toh kis cheez se dar rahi thi ap

Woman-02: mein is cheez se dar rahi thi mujhe dar lagta tha k kaise bacha hoga matlab kaise aayega haath daal k nikaaleinge pata nahi kia kia hoga pata toh nahi tha na k kaise delivery hoti hai

Interviewer: toh is dar k lihaaz se muwaazma kar sakti hain k ap ziada kis hispatal se dar rahi thin ya ghar k karne se darti thin

Woman-02: hispatal se karne se dar rahi thi

Interviewer: us ki koi wajha hai

Woman-02: isliye dar laga tha k mujhse aisa lag raha tha k pait kaat k bacha nikaaleinge pata nahi kaise taanke lageinge pata nahi kia kia hoga

Interviewer: jee jee sahi sahi acha toh ab mein poochna chah rahi thi k ap k ghar mein joh faisla karte hain woh kon hai? sab se bari joh hain ap k ghar ki ya yeh ap ki saas hain?

Woman-02: chachi

Interviewer: chachi ... acha toh un ka aur ap ka khayal ek hi tha ghar pe paida karne ka?

Woman-02: hmm

Interviewer: sahi .. thek hai toh ap ghar pe ya hispatal k elawa ap kis ko ziada bharosa karti hain delivery k liye

Woman-02: *Silent*

Interviewer: jaise kuch log TBA ko deite hain kuch health worker ko deite hain kuch apne ghar walon hi ko deite hain toh ap kis ko bharosa karti hain?

Woman-02: TBA ko

Interviewer: kia pehle se jaanti hain un ko?

Woman-02: wo meri parosan hain

Interviewer: parosan hain … thek thek thek acha toh phir un k sath bhi ache taaluq hain toh phir is tarhan se dil tha (10:14 – 10:16) k ghar pe paida kar lein

Woman-02: han

Interviewer: acha toh wohi ap k is delivery k liye wohi thi?

Woman-02: han

Interviewer: acha toh ap ki delivery kesi huyi thi? woh tajurba kaisa tha ap ka TBA k sath?

Woman-02: sahi tha

Interviewer: sahi tha…koi masle kuch bhi

Woman-02: nahi

Interviewer: nahi…sab kuch araam se hogaya tha toh ap ko un k sath ap ko kia faide nazar aate hain jis tarhan woh ap ka bacha jab deliver kar rahi hoti hain toh is k baare mein ap ko kia acha lagta hai joh ap ko shayad hispatal mein na acha lagta ho?

Woman-02: matlab woh aese ache se matlab bata bhi deity hain samjhaati hai achi tarhan se pesh aati hain

Interviewer: hmm hmm hmm

Woman-02: toh acha lagta hai hispatal mein toh chilaate rehte hain toh banda aur dar jata hai

Interviewer: han daante hain…ap ne kabhi suna hai kisi se k un k sath aesa k hispatal gaye thy un pe chila rahe thy ya aesa kuch?

Woman-02: han aese bohat saare sunein hain

Interviewer: suna hai ap ne?

Woman-02: han ek do ghanta ap k dardon mein lag jatay hai upar se ap kisi ki cheenkh chilaana bardasht karo

Interviewer: hmm hmm hmm

Woman-02: aur phir pehle bacha hota hai toh dar bhi hota hai

Interviewer: hmm hmm

Woman-02: k pata nahi kia hoga kia nahi

Interviewer: jee jee

Woman-02: jab bezati kaise bardash kare

Interviewer: bilkul jee jee

Woman-02: jab ghar mein TBA hoti hai na toh woh maa ki tarhan hoti hai

Interviewer: han bilkul bilkul sahi toh acha ab mein poochna chah rahi hun k ap ki joh saari sahuliyaat hain jaise hamare center pe ya hispatal pe woh ap araam se haasil kar sakti hain? koi masle toh nahi hote ap ko jaise center jaane mein mushkil horahi ho?

Woman-02: nahi

Interviewer: kabhi maslan kisi ap ne dawaai like kisi bhi dawaai k liye paise toh kharch nahi kiye?

Woman-02: nahi

Interviewer: sahi toh ap akele jaate ho center ya kisi k sath jaati hain

Woman-02: akele

Interviewer: akele jaati hain thek thek .. acha toh ap ko aane jaane mein koi masla toh nahi hota

Woman-02: nahi

Interviewer: aur ap ko gaari bhejte hain ya aese kuch hota hai

Woman-02: itna dur toh hai nahi

Interviewer: dur nahi ...acha toh paison k lehaaz se sahi lag raha hai kuch matlab bohat ziada kharcha toh nahi horaha hai dawaaiyon pe ya khidmaat mein ya aese kuch

Woman-02: dawaai toh yeh deite hain na

Interviewer: jee jee jee

Woman-02: leini toh nahi parti paison ki

Interviewer: ap ko kabhi paise deine pare thy kisi health worker ko ya kisi dawaai k liye

Woman-02: nahi

Interviewer: thek hai acha toh koi aur wajoohaat hain jis ki wajah se aap hispatal jaane se katraati hain?

Woman-02: samjhi nahi

Interviewer: jaise shayad hispatal dur hai ya bohat deir k liye rehna parta hai ya aisi koi cheez hai hispatal ki koi cheez achi nahi lagti hai ap ko us pe time ziada lagta ho us tarhan ki koi wajoohaat bataayein ek side pe toh ap ko dar bhi lag raha ho ap ne bure qissey sunein ho?

Woman-02: aise qissey toh sunein hain toh mein kabhi hispatal mein gayi nahi hun

Interviewer: hmm

Woman-02: bas yeh center pe

Interviewer: center pe jaati hain hmm… acha aur koi cheez hai joh ap batana chahein shayad ya hum ne poocha nahi aur ap batana chaheingi hamein

Woman-02: nahi

Interviewer: ghar pe paida karne k lehaaz se

Woman-02: nahi aur kuch nahi

Interviewer: thek hai …

___________________________X_____________________________

IDI WOMAN-03

Interviewer: acha Woman-03 sab se pehle mujhe poochna hai k ap ki umar kia hai

Woman-03: 18 saal

Interviewer: 18 saal acha ap k shadi ko kitna time hogaya hai

Woman-03: ek saal hone wala hai

Interviewer: ek saal hone wala hai aur ap k kitne bache hain

Woman-03: ek

Interviewer: thek hai sahi hai yeh ap ka pehla bacha hai

Woman-03: han

Interviewer: thek hai thek hai aur ap ne kitni Taaleem haasil ki hai

Woman-03: 10 tak

Interviewer: 10 tak thek aur ap kahin kaam karti hain ya ghar par rehti hain

Woman-03: ghar pe rehti hun

Interviewer: thek hai thek hai acha sab se pehle mujhe ap se poochna hai k bache paida karne ki joh jagha hoti hai sab se behtareen ap ko kia lagti hai konsi jagha hoti hai

Woman-03: hospital

Interviewer: hospital? Acha toh ap bata sakti hain k kyun

Woman-03: hospital mein koi takleef agar ghar mein takleef hoti hai toh hospital jaana zaroori hota hai

Interviewer: hmm

Woman-03: agar ghar mein takleef hoti hai toh hospital jaana zaroori hota hai

Interviewer: jee jee jee toh aur ap bata sakti hain k lekin ap ne ghar mein bacha paida kiya tha ap bata sakti hain k ghar pe paida karne ki koi cheez aap ko behtar lagti hai ap ko?

Woman-03: ghar mein toh kuch bhi acha nahi lagta bohat takleef hoti hai toh hospital sahi hai

Interviewer: hmm toh phir ap ne waise ghar pe kyun kiya bacha hm ...ghar pe kyun kiya bacha

Woman-03: achanak hogaya na

Interviewer: achanak hogaya ...toh

Woman-03: time poora nahi hua tha achanak hogaya tha

Interviewer: acha toh ek dam se matlab woh ap kehna chah rahi hain k un ko call kiya tha center walon ko

Woman-03: nahi achanak se hogaya na r

Interviewer: achanak se hogaya thek thek thek toh waise toh ap ka irada tha hispatal mein hi karein

Woman-03: han

Interviewer: acha thek hai toh aur mein poochna chah rahi hun k ghar mein agar karna ho toh us ki kia acha nahi lagta hai

Woman-03: ghar mein nahi sahi rehta

Interviewer: hmm kyun sahi nahi hai

Woman-03: ghar mein agar koi takleef hoti hai

Interviewer: hmm hmm...ap bata sakti hain k kin waqton mein hospital jana zaroori hai delivery k time pe

Woman-03: hospital toh raat tak khuli rehti hai

Interviewer: hmm hmm hmm hmm

Woman-03: subha tak khuli hoti hai toh poora hoti hai

Interviewer: han lekin jaise mein kehna chah rahi hun k waise toh phir ap ko hospital hi sahi lagta hai lekin ghar mein is liye karte hain kyun k ek dam se agar hojaaye bacha…ap yeh kehna chah rahi hain

Woman-03: han

Interviewer: thek hai toh ap k ghar mein sab bare joh hain sab kya kehte hain? kehte hain k hospital mein karna chaiye ya ghar pe karna chaiye?

Woman-03: hospital sahi hai

Interviewer: acha acha toh acha ab mein poochna chah rahi hun k ap hamare center pe arahi thi visit k liye woh joh khidmatein deita hai acha toh woh ap ko kaise lage thy

Woman-03: ache thy

Interviewer: ache lage thy toh ap kitni dafa aati thin

Woman-03: mein maheeney mein ek

Interviewer: ek maheeney mein jab bhi woh bulaate thy ap aati thin

Woman-03: han

Interviewer: acha toh hamare center pe kia cheez achi lag rahi thi

Woman-03: us ka ilaaj hai Xray karte hain

Interviewer: hmm hmm

Woman-03: dawaai bhi deite hain

Interviewer: jee jee aur koi cheez ap ko udhar ki achi shayad nahi lagi ho toh woh bhi bata sakti hain

Woman-03: bohat takleef hoti thi deir hoti thi toh ghar pe kaam bhi hote thy na

Interviewer: time lag jaata tha udhar jaane mein acha ab faasla ap ko kaisa lagta hai idhar se

Woman-03: bohat dur hai

Interviewer: dur hai acha toh aane jaane ka masla hota tha

Woman-03: han

Interviewer: acha toh yeh bhi kia masla tha pedaaish k time? ap ko laga tha k faasley ka masla hai?

Woman-03: han

Interviewer: toh waise ghar mein phone won hai jis se call kar sakein center ko?

Woman-03: mere khud k paas nahi shohar k paas hai

Interviewer: shohar k paas hai thek toh aese toh un ko delivery k time pe call hi nahi kiya tha

Woman-03: nahi kiya tha

Interviewer: acha toh yeh pata hai joh beithti hai na midwife center pe toh unhon ne ap ko samjhaya tha k jab delivery k time pe kia masle hosakte hain ap ko yaad hain k woh kia keh rahin thi

Woman-03: nahi yaad nahi

Interviewer: yaad nahi acha unhon ne kaha tha k delivery k time pe ap ko kia karna chaiye kia kaha tha unhon ne

Woman-03: *silent*

Interviewer: koi baat nahi joh bhi kuch yaad hai bata dein

Woman-03: bhul gayi

Interviewer: bhul gayi acha toh unhon ne samjhaya tha k ap ko call karna hoga sab ko?

Woman-03: han

Interviewer: ok agar unhon ne nahi bataya toh un ko kaheinge k ap se baat vaat karein toh aur koi chez hai joh udhar ki achi nahi lagi sahi se baat karte hain woh

Woman-03: han

Interviewer: acha aur ap ne koi buri cheez kabhi suni ho center k baare mein

Woman-03: nahi

Interviewer: aur hispatal k baare mein koi buri cheez suni ho joh ap k elaake mein joh dusri aurtein hain woh ghar pe hi karti hain ya hispatal mein mein karti hain

Woman-03: hospital mein

Interviewer: hospital mein karti hain aur un ka tajurba udhar kaisa gaya

Woman-03: doctor phir bhi ache thy

Interviewer: ache thy…woh kia keh rahin thi us k baare mein?

Woman-03: woh keh rahi thi k yeh idhar ghar mein hota hai toh ilaaj nahi hota hai toh hospital mein ilaaj zaroori hota hai

Interviewer: jee toh kis tarhan ka ilaaj hota hai

Woman-03: dawaai hoti hai

Interviewer: hmm hmm aur

Woman-03: teeke bhi lagte hain

Interviewer: jee acha toh ap ko pata hai k delivery k time par konse masle bhi hosakte hain ap ko yaad hai koi kaise ulta bacha shayad hota hai

Woman-03: han

Interviewer: aesi aesi cheezein yaad hai ap ko? aur kuch yaad hai us k baare mein?

Woman-03: paani bhi neeche hota hai na

Interviewer: paani bhi neeche hota hai…

Woman-03: khoon bhi hota hai

Interviewer: jee jee

Woman-03: mujhe toh paani hogaya tha

Interviewer: hmm hmm acha ap ko delivery k time pe kia masle huye thy

Woman-03: mujhe paani agaya tha

Interviewer: paani agaya tha

Woman-03: 6 din se pehle

Interviewer: acha toh phir bhi ap hispatal nahi gayin kyun

Woman-03: mein ne kaha yeh thora hai na

Interviewer: thora kia

Woman-03: mein ne kaha thora hai dard hai na toh mein ne us ko chor diya

Interviewer: hmm aur acha toh kya aap ko yaad tha k un (center) ko call kar sakti hain?

Woman-03: han

Interviewer: acha toh dard ap keh rahi thi k ziada nahi tha k ap call karein

Woman-03: han

Interviewer: acha toh ap keh rahi thi k 6 din k liye 6 din pehle hogaya tha

Woman-03: han

Interviewer: toh aur delivery k time aap ki normal delivery huyi thi

Woman-03: han

Interviewer: acha aur araam se bacha nikal gaya tha aur koi ghar pe aaya tha help karne k liye

Woman-03: nahi

Interviewer: madad karne k liye

Woman-03: nahi

Interviewer: TBA?

Woman-03: han aayi thi

Interviewer: TBA ayi thi? acha toh waise ap TBA ko jaanti hain

Woman-03: han

Interviewer: kis rishtey se ap jaanti hain un ko?

Woman-03: woh TBA hai na bohat achi hai

Interviewer: hmm

Woman-03: khayal bhi rakhti hai

Interviewer: hmm hmm hmm

Woman-03: woh apne upar zimedaari bhi leti hain

Interviewer: jee jee jee toh ghar mein joh dusre log hain unhon ne kisi aur k bache paida karwaye hain is ghar mein?

Woman-03: han

Interviewer: toh unhon ne kahan pe kiya hai?

Woman-03: unhon ne bhi ...(silent) (8:13-8:15)

Interviewer: koi baat nahi ap joh bhi sach hai wohi bataayein

Woman-03: kisi ko pata toh nahi chale (8:20- 8:23) pata nahi jagha bhul gayi hun

Interviewer: unhon nei (family member) ghar mein kiya hai ya hispatal?

Woman-03: pehle ghar mein tha woh bhi mere saath thi ek hi din mein thi

Interviewer: jee jee jee jee

Woman-03: toh us ka ghar mein nahi hua toh hospital chali gayi

Interviewer: hospital chali gayi thi ...toh ap ko bhi le jaate hospital

Woman-03: mera hogaya tha us se aage

Interviewer: us k masle horahe thy?

Woman-03: han

Interviewer: kia masle horahe thy?

Woman-03: us k bache neeche nahi arahe thy

Interviewer: us k bache kia sorry....

Woman-03: us k bache neeche nahi thy

Interviewer: neeche nahi thy jee acha toh us ko hispatal mein kaisa laga?

Woman-03: us ka phir operation hua

Interviewer: operation hua aur us k baad

Woman-03: us ka bacha mar gaya na

Interviewer: Allah… acha

Woman-03: us ki bhi beti thi

Interviewer: hmm hmm hmm

Woman-03: woh bhi ek hi din ki

Interviewer: acha ap ki beti hai kia naam hai us ka

Woman-03: Child-03

Interviewer: Child-03 acha mashallah bohat pyari hai acha toh woh kuch keh rahi thi shayad mujhe yeh karna chaiye tha yeh nahi karna chaiye tha un k kia khayalaat hain?

Woman-03: us ko bulaayein?

Interviewer: nahi nahi woh ap k saath baat toh ki hogi unhon ne ap se toh woh kia keh rahi thi

Woman-03: us ko dono taraf se bimaari thi na

Interviewer: hmm hmm hmm

Woman-03: toh us ka nahi hua

Interviewer: acha bimaari kaisi kis cheez ki?

Woman-03: pata nahi woh konsi bimaari thi joh us ka samjh mein nahi aaya

Interviewer: acha toh masla ap ko lag raha hai k un ki taraf sei tha ya hispatal ki taraf sei tha?

Woman-03: center k toh thy woh un k number nahi uthaaye

Interviewer: hmm hmm

Woman-03: unhon ne bhi phone ki thi

Interviewer: acha center pe phone kiya tha

Woman-03: han

Interviewer: aur phone nahi uthaya

Woman-03: nahi uthaya

Interviewer: toh yeh kis time pe hua tha takreeban subha?

Woman-03: mere saath 3:30 bajhe gayi thi

Interviewer: sahi sahi

Woman-03: lekin mera 6 bajhe hua tha takreeban

Interviewer: acha acha acha

Woman-03: toh us k subha se 6 bajhe mere saath gayi thi na toh phir woh us k bhi sham k 6 bajhe hua tha

Interviewer: hmm jee jee jee acha toh phir ap keh rahi thi k center pe phone kiya tha lekin phone nahi uthaya us ko phir bhi hospital le k chaele gaye ap

Woman-03: han

Interviewer: kis tarhan se ap k paas gaari hai?

Woman-03: *Silent*

Interviewer: acha center pe phone kiya tha

Woman-03: apni gaari se

Interviewer: apni gaari se acha

Woman-03: unhon ne toh gaari nahi di thi

Interviewer: sahi acha aur sahi time pe pohanch gaye thy woh udhar

Woman-03: nahi pohanche tabhi toh bacha fout hogaya

Interviewer: jee jee sahi hmm afsos ki baat hai bohat han toh…toh hospital waale sahi se baat karte hain kyun k kabhi kabhi mein sunti huyi arahi hun k sahi se baat nahi karte?

Woman-03: sahi se nahi karte

Interviewer: acha bas time hogaya tha aur kuch unhon ne galat kiya tha

Woman-03: nahi hum log yahan se jaate thy na toh yahan se bhi paidal jaate thy wahan se bhi paidal aate thy hamein gaari nahi di thi

Interviewer: jee jee acha sahi hai acha ab mujhe poochna hai k aur un ka pehla bacha tha

Woman-03: han

Interviewer: acha sahi sahi sahi toh ap k ghar mein joh deakhbhaal aur sab faisle waisle kon karta hai

Woman-03: is ki bhaabi (11:56-11:59)

Interviewer: yeh beithy hain ap k shohar (in the other room)? Acha toh ap ki joh marzi thi aur un ki marzi ek saath thy khayal k hispatal mein hi karna hai

Woman-03: han

Interviewer: ok acha ab mein poochna chah rahi hun k TBA bulana ap ko sahi lag raha tha matlab woh achi hain unhon ne ache se deliveries ki hain pehle bhi acha toh woh kis tarhan se ap ki deakhbhaal kar rahin thi aur kis tarhan se ap ka khayal rakha delivery k time pe?

Woman-03: sahi sahi

Interviewer: sahi se Rakha? Kis tarhan?

Woman-03: hmm

Interviewer: un ki kia sab se achi cheez kia lagi ap ko

Woman-03: woh us waqt mujhe bohat takleef thi na toh woh araam se kara rahi thi

Interviewer: araam se kara rahi thi?

Woman-03: ahista ahista

Interviewer: sahi hai sahi hai thek hai aur us k baad bhi jab bacha nikal k aata hai toh phir us k baad joh bhi ilaaj ya sab kuch karwana tha woh kaise kiya... unhi se karwaya?

Woman-03: han

Interviewer: acha center nahi gayin?

Woman-03: nahi

Interviewer: ok ok toh unse karwana ap ko sahi kyun laga tha un ki kia achi cheez thi?

Woman-03: unhon ne jab mera bacha paida kiya na

Interviewer: hmm hmm

Woman-03: toh mujhe us ka sahi laga

Interviewer: center pe ap ko bharosa hai?

Woman-03: han

Interviewer: acha wohi mein poochna chah rahi hun kyun k agar koi aesa masla ho na toh woh hum log thek karna chahte hain toh center ki koi bhi cheez ilaaj vilaaj agar koi bhi cheez nahi pasand ho to aap hum ko bata sakti hain ta k hum behtari laa paye

Woman-03: woh hum log yahan se jaate thy na

Interviewer: hmm hmm

Woman-03: toh hum beith beith k thak jaate thy

Interviewer: time lag jaata hai?

Woman-03: han toh number pe bulaate thy toh deir hojaati thi

Interviewer: jee jee sahi toh acha ab mujhe poochna hai k aur ap ko yeh center pe jaana sab kuch ap ko matlab fasla ka ap ko lagta hai k masla hai?

Woman-03: han

Interviewer: faasle ka masla hai aur ap keh rahi thin k transport matlab udhar jaane ka joh system hai woh bhi masla hai

Woman-03: han

Interviewer: thek hai aur mujhe poochna hai jab ap jaati hain toh kis k sath jaati hain

Woman-03: hum 3, 4, 5, 6 sab (gesturing family members)

Interviewer: sab jaate hain?

Woman-03: han

Interviewer: thek hai thek hai aur udhar kia ap ko paison ka masla toh nahi hua hai k paise kabhi deine pare ilaaj k liye

Woman-03: nahi

Interviewer: nahi…

Woman-03: han, center pet oh sab muft mein hai na

Interviewer: aur TBA k liye aap k aap nei paisay diye?

Woman-03: TBA k liye nahi

Interviewer: nahi deina parta?

Woman-03: han

Interviewer: ok ok acha...toh ab mujhe poochna hai k toh ab mein woh mawaazma kei tor par pooch rahi thi k ek mawaazna kar sakte hain k TBA aur hispatal k beech mein kia ap ko behtar lagta hai? un ki kia kharabiyaan ya khubiyaan hain kuch bhi?

Woman-03: *silent*

Interviewer: k shayad TBA kisi cheez mein achi na ho hispatal ache nahi ho ya hispatal ki koi cheez achi nahi ho

Woman-03: agar TBA se nahi hota hai toh hospital mein sahi hai

Interviewer: hmm toh phir is ka matlab k ap TBA se karwaana chah rahi thi ...toh pehle TBA se phir agar woh sahi nahi hota phir hispatal se

Woman-03: han

Interviewer: ok toh acha thek hai toh ap ki joh ap ki behan hai jis ne dusra bacha diya?

Woman-03: nahi nahi meri khala ki beti

Interviewer: khala ki beti toh…kya woh bhi pehle koshish kar rahi thi k TBA se hojaaye?

Woman-03: han

Interviewer: toh phir us mein kitna time laga tha

Woman-03: us ko poora din lag gaya tha

Interviewer: Allah… toh phir poora din k baad

Woman-03: 6 bajhe se samjho us ki beti paida hogayi poora din meri subha 6 bajhe us ki sham 6 bajhe

Interviewer: acha jee jee jee ap ne bataya tha toh poore din woh TBA k saath thi?

Woman-03: nahi hua toh phir hispatal chali gai

Interviewer: acha

Woman-03: toh tabhi toh us ki beti mar gayi

Interviewer: Oh...acha acha sahi toh TBA k bare mein ap kia sunte huye arahi hain kyun k acha hota hai ilaaj ya kuch logon k jaise ap ki khala ki beti ka hua tha ap yeh ziada sunte huye arahi hain ap ne un k bare mein kia kia suna hai dusron se?

Woman-03: dusre bhi kehte hain sahi hai TBA se karwana

Interviewer: hmm hmm hmm hmm aese kis ne mashwara diya? Kya who TBA ko bulaate hain?

Woman-03: sab log kehte hain

Interviewer: sab log kehte hain ...ghar mein kon kehta hai

Woman-03: meri khala yeh beithi hai

Interviewer: acha khala sahi hai aur koi cheez hai joh ap batana chahein shayad mein ne poocha nahi ho koi bhi cheez aesi ho

Woman-03: nahi

Interviewer: aur center k bare mein aur kuch kehna chaheingi k hum kia behtar kar sakte hain ilaaj k tor par ya jis tarhan se doctor baat karte hain

Woman-03: hum logon se khoon nikaalte thy lekin sahi ilaaj nahi hota tha

Interviewer: acha toh khoon nikaalte thy

Woman-03: test karte thy

Interviewer: hmm hmm

Interviewer: waise dawaaiyan deite thy ap ko?

Woman-03: han

Interviewer: toh kis tarhan se ap ko lagta tha k sahi nahi horaha kis tarhan se lagta tha sahi nahi hora hai ilaaj

Woman-03: sahi se dawaai bhi nahi deite thy

Interviewer: acha toh kis lehaaz se matlab kam deite thy?

Woman-03: han

Interviewer: kam deite thy shayad woh chahte thy ap thek se aayein…

Woman-03: *laughs*

Interviewer: acha toh waise woh kia kehte thy kya khaali goliyaan deite thy kia samjhate thy ap ko kuch…kis tarhan leina hai aur kyun…

Woman-03: khaali goliyaan pakra detei thy

Interviewer: hmm aur unhon ne samjhaya ap ko k yeh dawaaiyan kyun de rahe hain

Woman-03: nahi

Interviewer: nahi samjhaya ok sahi hai toh ap ki joh khala ki beti hai woh bhi arahin thi center un ka sahi tha udhar tajurba woh sahi tha?

Woman-03: wo to aap un sei pata karlein…

Interviewer: acha toh aur kuch hispatal k bare mein center k bare mein joh hum behtar kar sakte hain

Woman-03: nahi

Interviewer: nahi acha thek hai bohat bohat shukriya ap ka...

______________________________X______________________________

IDI WOMAN-04

Interviewer: sab se pehle ap mujhe baata sakti hain apki umar kitni hai?

Woman-04: 30 saal

Interviewer: apki shadi ko kitna time hogya?

Woman-04: 8 saal

Interviewer: or ap ke bachey kitney hain?

Woman-04: 5

Interviewer: ap ke saarey bachey kahan kahan kiye hain ap ne?

Woman-04: 2 larkey ghar par 3 larkiyan hospital par, ak larki to gaari mein jatey huwe gaari mein hi hogai

Interviewer: to ap keh rahi hain 3 ghar par or 2 hospital mein huwe?

Woman-04: jee, naam likhwa deti hun lkn agar ghar par ho raha hota hai to ghar par warna Jahan saholat lagey

Interviewer: apko behtreen jaghan konsi lagti hai bachey paida karne ke liye?

Woman-04: hospital par

Interviewer: ap baata sakti hain kyun behtar lagti hai?

Woman-04: door nahi hai

Interviewer: is ke ilawa koi waja?

Interviewer: dekh bhaal bhi ache se hoti hai, ilaaj bhi acha hota hai

Interviewer: apko ghar mein paida karne se kya acha laga?

Woman-04: nahi ghar mein acha nahi hai, ghar se acha hospital hai ghar mein ilaaj nahi hota

Interviewer: ghar mein jo apka tajruba raha us mein koi asa bura waqiya raha?

Woman-04: hamari ghar ki TBA hai meri daadi hain to asa koi masla ho ke ghar par karna parey to wo karwa deti hain

Interviewer: ghar par karne se koi nuqsanat kabhi dekhney parey?

Woman-04: nahi bas ak larki bohat kamzor huwi thi

Interviewer: ghar par ksi barey se sunti aa rahi hain ke ghar ziada behtar hai hospital se?

Woman-04: nahi, wo bhi yehi khete hain hospital ziada behtar hain

Interviewer: jese apki dadi hain jo TBA hain wo bhi yehi smjhti hain k ghar ziada behtar hai?

Woman-04: wo TBA hain to pehle un ke paas hi jate hain to wo phir case ke hisaaab se baata deti hain ke hospital behtar hai ya ghar par ho jaega warna wo bhi hospital bhej deti hain

Interviewer: apo kya lagta hai ase kon se masael hain delivery ke doraan jinki waja se hospital jana par jata hai?

Woman-04: hamal ke doraan se pehle koi masla agar aajay to mein us se pehle hi dikhane chali jati thi kyun ke unho ne kaha tha k dekivery se pehle koi bhi masla ho to aajaya karen to mein chali jati hun

Interviewer: ap hmarey center mein apney hamal ke doraan kitni dafa aati thin?

Woman-04: 8 dafa gai thi jab wo bulatey thy

Interviewer: acha or unka ilaaj apko sahi lagta tha center mein apko kya kya cheezen achi lagi thin?

Woman-04: pure hamal ke doraan meinne teesrey (3) month mein apni file banwai wahan jati rahi wo mujhey folic acid or paracetamol dete rahe 4 months tak, phr us ke baad abhi jo cheezen mamta mein mil rahi thin jo ab band kardi or har follow up par jati thi is k elawa bhi agar beech mein meri tabyat kharab hoti jese pet ka drd bhi hota tha to uske liye bhi ilaaj kar ke goliya de dete the or mujhey hamal ke doraan qabz bhi rheti thi to uska alag se ilaaj karte or agar khoon ki kami end tak aajati thi to drip wgera bhi lagi thin pure hamal or delivery tak koi masla nahi huwa

Interviewer: or jab wo apka ilaaj kartey hain yaa dawai dete hain to sahi se samjhatey hain apko?

Woman-04: jee han, meri akhri bachi ke doraan ye bhi btaya tha ultrasound mein ke bacha kazmor hai to uski phir dawaiyan bhi di thin

Interviewer: jab ap hamal ke doran center ja rahin thin to delivery wagera ki koi tareekh li thi ap ne?

Woman-04: han lakin waqt se pehlehogya tha

Interviewer: to waqt se pehlehone ki waja se ap ne ghar par kiya tha bacha?

Woman-04: han achanak hogya tha islye

Interviewer: ap ke sarey bachon ke lye ap center gaen thin?

Woman-04: han teekey bhi lagway the

Interviewer: achi baat hai

Woman-04: or asa koi masla raha center jane mein ke faasla ho ya koi or baat?

Woman-04: nahi itna door nahi hai

Interviewer: or agar gaari bulwa len to wo aajati hai?

Woman-04: han wo gaari bhi dete hain

Interviewer: acha mujhey ye baataen jab apki delivery ka time aa raha tha to unhon ne apko ye sab smjhaya tha ke kya masley ho saktey jese bacha ulta bhi ho sakta hai ya kuch or?

Woman-04: han mujhey baataya tha k apka bacha ulta hail lakin humney dam wagera karwaya to mushkil nahi huwi Allah ke karam se

Interviewer: acha is ke ilawa or kuch baataya tha jese khoon bohat nikal jata hai wagera?

Woman-04: han khoon ki kami ki waja se kaali wali goliyan bhi di thi

Interviewer: acha or wo baatate the ke kyun de rhe goliya waja wagera?

Woman-04: jee han bohat acha raha ilaaj pura bohat acha raha bas time se pehleki waja se jaldi hogay the ghar mein

Interviewer: acha jo ghar mein huwe bachey wo time se jaldi hone ki waja se ghar par huwe?

Woman-04: han bas shuru ke jo bachey hain jo ghar mein huwe unki dafa mein center ka kuch pata hi nahi tha us ke baad jab pata chala to phir har dafa mein center mein naam likhwaya

Interviewer: kon se bachey ke waqt center jana shuru kya?

Woman-04: 3srey number wali par center jana shuru kya, 4 bhi ceter mein huwa baaqi ye 5 number wali achanak hogai

Interviewer: acha jee ab mujhey puchna tha ke unhon ne sahi se smjhay tha ke delivery ke waqt ap gaari bhi mangwa sakti hain call karni hogi ye sab?

Woman-04: jee han sab smjhaya tha

Interviewer: acha ghar ke tamam faisley kon karta hai?

Woman-04: susar

Interviewer: unka kya khayal hota hai ke ghar mein bacha karna chaiye ya hospital mein?

Woman-04: nahi wo bhi khete hain hospital mein

Interviewer: jesa aap ne kaha ke TBA jo hain jo apki daadi bhi hain unki baat par un ke mashwarey par bhi aap yakeen rakhti hain bharosa karti hain to kabhi unhon ne koi aesi baat kahi ya samjhai ho jiski waja se apko dekivery mein masla huwa ho?

Woman-04: nahi achanak agar koi masla hojay to wo bhi center janey ka hi kheti hain

Interviewer: apko wese to ap ne kaha center jane mein faasley ka masla nahi hota or wo gari bhi bhijwa dete hain, ye btaen aap center jati kis ke saath hain?

Woman-04: meri daadi ke saath

Interviewer: acha apko kabhi dawaiyon ya ilaaj par kharcha karna para?

Woman-04: nahi sab center se huwa

Interviewer: matlab apko faasley ya kharchey ka masla nahi hota ap har lihaz se mutmaen hain un se? raazdaari rakhtey hain wo log?

Woman-04: nhai koi masla nahi hota, jee raazdari bhi rakhtey hain

Interviewer: acha ye baataen apka hospital mein tajruba kesa raha

Woman-04: Ma Sha Allah acha raha koi masla nahi raha

Interviewer: or ap se sahi baat kartey hain the doctors wagera daant'tey to naho the apko?

Woman-04: nahi asa kuch nahi

Interviewer: or is k ilawa koi aesi baat wahan ki jo apko achi na lagi ho or ap baatana chati ho kyun ke hum usey behtar karna chate hain

Woman-04: sab acha tha koi aesi buri baat nahi rahi bas delivery ke doraan thora daant rahe the or takleef bhi di thi bas

Interviewer: takleef kis tarhan de rahe the?

Woman-04: delivery ke baad jo aurat ki safai wagera hoti hai us waqt bhi mene kaha ke mujhey sunn kar do taa ke takleef kam ho to us par bhi jese mujhey kaha daant kar ke theek hai phir uzma api ko bulatey hain phir wohi karengi injection laga kar to phir mein chup hogai

Interviewer: oh nahi asa nahi karna chaiye tha unhain

Interviewer: or koi bhi baat ya cheez jo hospital jane mein rukawat bantey ho delivery k liye jane ke liye?

Woman-04: nahi koi masla nahi hota center door nahi hai ziada

Interviewer: to agley hamal ke liye bhi ap hospital jana chahengi ke hospital mein hojay?

Woman-04: jee hospital mein hi sahi hai, ghar par to nahi hai ilaaj dawai wagera, baaqi hospital na jane parey sarey kaam center mein hi hojaen to ziada behtar hai, center mein ziada behtar lgata hai

Interviewer: or koi aesi baat ya cheez jo aap baatana chahein jo hum pooch na saken ho?

Woman-04: ghar par bas ye khof hota hai ke khoon choot jay bachey ko kuch hojay wagera to ye cheezen ghar par nahi hoti e sab ache se center mein hi hota hai, doctor wagera bhi sab ache hote hain, khoon ki kami ka bhi baatatey hain phir dawai bhi dete hain, ultrasound mein bhi sab baatatey hain ke bacha kamzor hai ya nahi to sab behtar hai center mein

Interviewer: sahi sahi to mujhey lagta hai saarey sawal hogaye, shukriya

___________________________X_____________________________

IDI WOMAN-05

Interviewer: apki umar kitni hai?

Woman-05: 28 saal

Interviewer: apki shadi ko kitna time huwa hai?

Woman-05: 11 saal

Interviewer: ap ke kitney bachey hain?

Woman-05: 4

Interviewer: taaleem kya hai apki?

Woman-05: taaleem hasil nahi ki

Interviewer: apko bacha paida karney ke liye konsi jagha sahi lagti hai?

Woman-05: ghar par hi

Woman-05: waja?

Woman-05: ghabrahat hoti hai mujhey hospital mein

Interviewer: kabhi kisi se kuch suna hai koi bura tajruba raha hai jiski waja se apko ghabrahat hoti hai?

Woman-05: nahi, wese to mere bachey family clinic mein paida hote hain lakin ye akhri wali bachi ghar mein paida huwi hai

Interviewer: phele jo bachey clinic mein huwe un mein koi bura tajruba raha clinic mein jiski waja se ghabrahat hoti hai?

Woman-05: nahi

Interviewer: acha hospital mein ghabrahat hoti hai to ghar mein asi kya cheez hai jo ap ko pasand hai or jiski waja se yahan ghabrahat nahi hoti?

Woman-05: hospital jana acha nahi lagta dar lagta hai phele kabhi gai bhi nahi or ghar mein jo saholat milti hai wo hospital mein achi nahi lagti

Interviewer: koi or janne wala jo hospital jaa chuka ho?

Woman-05: meri choti behan us ke bachey hospital mein hi hote hain

Interviewer: or sahi huwe hain un ke bachey?

Woman-05: jee

Interviewer: kitney bachey huwe hain un ke wahan?

Woman-05: 3 bachey

Interviewer: ap tafseel se bata sakti hain ke ghabrahat kis cheez ki hoti hai apko? kya bura lagta hai?

Woman-05: buri koi cheez nahi lagti bas acha nahi lagta ase hi

Interviewer: kabhi kisi ka koi asa bura tajruba suna hai?

Woman-05: nahi wese hi mujhey acha nahi lagta

Interviewer: ap ke sarey bachey ghar par huwe hain?

Woman-05: nahi 3 bachey clinic mein huwe hain ye ak ghar par huwi hai

Interviewer: dusrey bachey family clinic mein kyu huwe thy?

Woman-05: wo time par ay thy islye

Interviewer: family clinic kahan par hai?

Woman-05: yahin qareeb mein hai aage gali mai

Interviewer: idher koi doctor hote hain ya TBA?

Woman-05: jee aik Dr. F hain

Interviewer: kabhi hamare center kei saath bacha huwa hai?

Woman-05: nahi

Interviewer: ap center mein aati hain?

Woman-05: jee, is akhri beti ki dafa mein bhi aai thi mera naam likha huwa hai

Interviewer: kitni dafa aati thin ap hamal ke doran center?

Woman-05: mein phele ak dafa aai naam likhwaya phir 2 3 dafa checkup huwa

Interviewer: to apko center kaisa laga tha?

Woman-05: mujhey sab cheezen sahi lagti hain bas hospital jana acha nahi lagta

Interviewer: center mein ap se thyek se paish aate hain health workers?

Woman-05: theek thy…

Interviewer: center ki kya cheez apko sab se achi lagti hai?

Woman-05: checkup sahi hota hai

Interviewer: apko pata hai ke kin waqton mein hospital jana zaroori hota hai?

Woman-05: jee

Interviewer: apko lagta hai ke hospital sahi hai ya ghar?

Woman-05: phele shuru mein lagta tha ke hospital sahi hai lakin phir family clinic mein hone lagay to phir ab hospital acha nahi lagta

Interviewer: apko family clinic or hospital mein kya farq lagta hai?

Woman-05: ak to fasla or phir wese bhi mujhey family clinic sahi laga

Interviewer: apko family clinic ke barey mein kya cheez ziada sahi lagi achi lagi?

Woman-05: dekh bhaal ache se hoti hai

Interviewer: to hospital mein apko lagta hai ke time ziada lagta hai ya door lagta hai?

Woman-05: hospital mein gai nahi hun islye mein apko bata nahi sakti

Interviewer: jese ap ne kaha hospital se apko ghabrahat hoti hai lakin family clinic main sahi lagta hai, asa kyu?

Woman-05: mein gai hi nahi to mein bata nahi sakti

Interviewer: or kya wujoohat hain family clinic mein karwane ki? kisi se suna hai ke clinic acha hai?

Woman-05: nahi bas mein khud karwati hi wahin se hun sab checkup wagera bhi

Interviewer: is ke ilawa ap ke ghar walay ka kya khayal ke pidaesh ke liye konsi jagha bhetar hai?

Woman-05: nahi hamarey meke mein bhi sab ke bachey ghar mein huwe meri ammi ke lakin ab bhabiyan wagera jo hain wo hospital hi jati hain lakin mere khayal ammi jese hain

Interviewer: hamare center mein kuch asa jiski waja se ap ne kaha ho ke mein hospital mai nahi karungi?

Woman-05: jee nahi ap ke center se koi taaluq nahi hai iska

Interviewer: apko CE center NTER par aane ke bad bhi asa kyu laga ke ghar mein ya family clinic mein krna chaiye hospital mein kyu nahi?

Interviewer: bas ase hi or hospital mein kabhi jana bhi ho to phone nahi lagta or lag bhi jay to larki nakhrey ziada karti hai jab lene aajati hain to nakhrey karti hain ke jaldi karo ye karo wo karo hum bata dete hain phele hi lakin wo us din nahi aati dusrey din aati hain

Interviewer: acha asa kis ke saath huwa hai?

Woman-05: ye phele mere bachon ke saath hi huwa hai naam likhwane ke baad bhi wo checkup karney nahi aay center wale

Interviewer: apki delivery ke time ap ne center par call ki thi?

Woman-05: meine 7 mahine intezar kya tha phir khud ja kar center mein naam likhwaya tha ke koi bat hojay koi masla hojay to hospital jaa to saktey hain na

Interviewer: ap ke khayal mein konse masley hote hain jinki waja se hospital jana zaroori hojata hai?

Woman-05: koi bhi masley ho saktey hain jese calcium ki kami hojati hai blood pressure low ya high ho jana

Interviewer: apki delivery mein asa koi masley ap ke saath huwe thy?

Woman-05: jee blood pressure low ziada rehta hai mera

Interviewer: or apki sari normal deliveries thin?

Woman-05: jee

Interviewer: acha ye btaen ghar ke tamam faisley wagera kon karta hai?

Woman-05: meri saas karti hain ya phir susar

Interviewer: un ke khayal mein bhetar kya lagta hai unehn apki delivery ke liye?

Woman-05: unhain to hospital thyek lagta hai kyu ke mere bachey ultey hote hain na to unhain hospital thyek lagta hai lakin mujhey hospital acha nahi lagta

Interviewer: ap family clinic mein ja rahi hoti hain to Dr. F ke pas bhi jati hain? or center par bhi jati hain?

Woman-05: jee tabyat kharab wagera hoti hai to donu taraf chali jati thi

Interviewer: apko donu mai kya sahi lagta hai?

Woman-05: jo qareeb lagta hai wahin chalu jati hun

Interviewer: to center mein faasley ka masla hota hai bs apko?

Woman-05: jee phele wo chorney or lene donu aate thy to mein chali jati thi lakin ab sirf chorney aatey hain to islye main ahi jaa paati kyu ke ghar mein le janey wala koi nahi hota koi nahi le kar jata sab apne kamo par jate hain

Interviewer: or Dr. F ke yahan ap kese jati hain phir?

Woman-05: wo to qareeb hai na to mein pedal bhi chali jati hun

Interviewer: saath kon jata hai ap ke?

Woman-05: meri saas jati hain

Interviewer: koi or rukawaten jinki waja se ap center nahi ja sakti?

Woman-05: bas yehi ke gari chorney aati hai lene nahi

Interviewer: to ap ne bat ki hai?

Interviewer: jee ki hai wo kehte hain ke hum lene aate hain to aksar bohat intezar karna parta hai logon ke ghar ke bahar or aksar phir log saath aane se mana bhi kar dete hain islye hum sirf chorney aate hain

Interviewer: apko kabhi paise kharch karney parey thy? Dr. F ke liye ap kya karti hain?

Woman-05: wo to fee leti hain na

Interviewer: to wo ap de pati hain?

Interviewer: jee

Interviewer: or center mein apke paise nahi lagtey ye pata hai apko? or ye cheez kesi lagti hai apko?

Woman-05: jee achi lagti hai

Interviewer: Dr. F jo ap ki hain wo delivery ke kya paise leti hain?

Woman-05: 5 hazar

Interviewer to ap de pate hain asani se?

Woman-05: jee jee

Interviewer: is k ilawa or kya kya facilities deti hain apko?

Woman-05: dawai wagera deti hain teekey wagera lagati hain zaroorat parti hai to drip wagera lagati hain

Woman-05: acha or ap se wo ap ke hamal ke doran baten karti hain apko guide karti hain ke kab kese kya karna chaiye apko?

Woman-05: jee batati hain sab

Interviewer: wo apko kya dawai deti hain?

Interviewer: ak rat ka ak subah ka capsule taaqat ka mene kaha tha unhain ke mujhey bp low rehta hai

Interviewer: bacha ka ilaaj ap kahan se karwati hain?

Woman-05: kahin se nahi

Interviewer: in ke bhi jo teekey wagera hote hain phir wo kahan se lagwati hain?

Woman-05: wo pedaaish teekey to yahan pas mein camp laga tha wahin se lgwa lye thy

Interviewer: kya apko pata hai ye saholat center mein bhi mujood hai?

Woman-05: jee pata hai mene apne 3 bachon ke teekey wahin se lagway hain

Interviewer: mein ap se phir poochna chaongi ke koi aas pass ap ne kisi se suna ho ke unhain hospital acha nahi laga ya unka hospital ka tajruba acha nahi raha?

Woman-05: nahi

Interviewer: apki behan ki dafa mein ap ne bataya un ke sath hospital walon ka rawaiya acha nahi tha to us barey mein ap ka kya khayal hai wo apko kesa lagta hai?

Woman-05: ye to mujhey nahi pata main nahi gai thi un ke saath meri saas gaen thin

Interviewer: uski waja se to apko ghabrahat nahi hoti hospital se?

Woman-05: nahi

Interviewer: phir kya cheez hai jis se ghbrahat hoti hai hospital jane mein?

Woman-05: bas ase hi jo cheez normal ho rahi hai us ke liye hospital kyu jaen

Interviewer: to apko ye dar lagta hai ke wo hospital mein operation wagera hojaega ya is tarhan ka koi dar?

Woman-05: nahi asa kuch nahi hai mein jab checkup karwati hun to wo kehte hain normal hai

Interviewer: acha mein ap se ye puchna cha rahi hunk e hospital ki asi kya cheez hai jo apko nahi pasand actually phir hum us par amal karna chate hain na us cheez ko sahi karney ke liye

Woman-05: jee nahi sab kuch sahih ai bas mujhey acha nahi lagta ke mein bahar apni delivery karwaon

Interviewer: apko ghar par thyek to lagta hail akin ap delivery to family clinic mein hi karwa rahi hain na ya wo ghar aati hain ap ke pass?

Woman-05: jee nahi mein clinic jati hun jab bhi mujhey zaroorat parti hai

Interviewer: ap ke liye delivery ke time sab se aeham cheez kya hoti hai kis cheez ki zaroorat hoti hai us time?

Woman-05: apne apko sambhalna or bachey ko sambhalna

Interviewer: or us waqt ap kya chati hain ke ap ke pass kon ho?

Woman-05: meri saas ya phir Dr. F jo hoti hain wo bhi bharose mand hain

Interviewer: or apko TBAs kesi lagti hain?

Woman-05: TBAs bhi yahin hain or doctor bhi mein Dr. F ke pass hi jati hun family clinic

Interviewer: acha TBAs ap ki behan ya ap ke khandan mein kisi ke lye nahi aati yahan asa kyun? apko TBA sahi nahi lagti?

Woman-05: family doctor ziada sahi lagti hai

Interviewer: acha family clinic mein asa kya hai jo hospital mein apko lagta hai nahi hai?

Woman-05: bas wo thyek se checkup karti hain thyek se bat karti hain jo cheez samajh nahi aati wo dubara puch lete hain.

Interviewer: to sab se bara hospital jane ka masla faasla hai ap ke liye?

Woman-05: jee door hai

Interviewer: lakin wo gari bhijwate hain hospital ke liye to ap chahein to unhain call kar ke gaari mangwa sakti hain

Woman-05: jee number hai hamare pas

Interviewer: or us ke ilawa ap keh rahi thin ke ap ghabrati hain hospital se

Woman-05: jee

Interviewer: or Dr. F jo ap bta rahin hain unehn ap kese janti hain?

Woman-05: meri saas ke bachey bhi wahin huwe thy

Interviewer: oh acha to wo purani hain?

Woman-05: jee

Interviewer: or koi cheez asi jo ap batana chahein ya hum pooch na sake ho?

Woman-05: jee nahi bas

Interviewer: okay jee bohat shukriya

___________________________X_____________________________

IDI WOMAN-06

Interviewer: ap kitney saal ki hain?

Woman-06: 26

Interviewer: ap ki shadi ko kitna time huwa hai?

Woman-06: 5 saal

Interviewer: ap ki taleem kya hai?

Woman-06: 3 class

Interviewer: ap ke kitney bachey hain?

Woman-06: 2

Interviewer: acha or ye akhiri wala kitne saal ka hai abhi?

Woman-06: 40 din ka

Interviewer: ye bataein ap ghar par kam karti hain ya kuch or kaam karti hain?

Woman-06: ghar par rehti hun

Interviewer: apki nazar mein bacha paida karney ke liye konsi jagha sahi hai?

Woman-06: pehle to hospital sahi tha meri pheli jo beti huwi wo bilkul sahi se huwi abhi is dafa mein theek se nahi kya paani choot gaya mujhey raat mein 2:30 par to mein nei center ko phone kiya unho nei kaha abhi sahih hai subah aa kar opd mein dekha dena phir subah mein 5 ghantey ke lye hospital mein daakhla rakha phr ak doctor ne aa kar kaha abhi chutti karo lakin dekha to paani phir bhi ja raha tha lakin 2, 3 ghantey or rakh kar kaha ke ye goli lelena abhi hamarey bas main nahi hai bacha abhi chota hai ase keh kar mujhey janey ke liye bol dya to mein ghar aagai phir mujhey ghar aa kar subah tabyat kharab hogai mjhey washroom janey ke saath hi nikal aagaya (bacha) mujh se to meri nani TBA hain unhain bulaya phir unhon ne sab kya , us ke bad mein phir hospital gai mene center pei health worker ko phone kya to unhon ne kaha gaari bhej rahe hain maa bachey donu ko bhejo phr wahan gai direct chekup wagera kya phr 12 baje ak doctor aata hai usey bachey ko dkhaya unhon ne ak powder wala sharbat dya or kaha abhi le kar jao bacha abhi sahi hai haftey wale din lazmi dekhana , phir dikhane ke liye gay hafte ko to kaha idher to jaghan khaali nahi hai or bacha bhi bilkul theek nahi tha neela or peela par raha tha in logon ke aasrey mein hum ne bachey ko rakh dya kahin or le kar bhi nahi gay phir doctor ne kaha isko piliya hai wo sir mein char kar pagal bhi ho sakta hai lakin abhi jaghan khali nahi hai abhi jao asa bol kar bhaga dya phir mene ghar par ak or din sabar kya unhon ne kaha tha gaari bhejenge lakin nahi bhej bacha pura neela peela ho raha tha to mein aik IHN chali gai udhar walon ne chote IHN mein bheja or 5 din rakha phir unhon ne JPMC bhej dya kyu ke inhain ulti thek nahi ho rahi thi JPMC mein inhain 8 din rakha phir aj opd mein jana hai isey le kar

Interviewer: jee ye achi bat nahi hai ap ke saath asa huwa

Woman-06: jee pheli dafa mein sab bilkul theek tha lakin is dafa pata nahi kya huwa un logon ko bachey ko bhi nahi rakha na mujhey theek se dekha jab ke hamare yahan 3 bahu ka wahin par huwa KGH mein

meri pheli beti bhi 7 mahine mein hogai thi KGH walon ne hi sab dekh bhaal ki or is bachey ko to intezar hi intezar mein neela peela kardya

Interviewer: acha pheli beti ki dafa mein apka tajruba theek tha?

Woman-06: jee bohat acha tha tab ache se dekh bhaal ki thi or gaari bhi aati thi pehle lene ab ke khud jana para lakin phir bhi gai thi

Interviewer: to apko hospital hi pasand hai lakin is dafa apka doctors kei saath tajruba theek nahi raha asa hai?

Woman-06: jee asa hi hai

Interviewer: acha phir next time apka bacha hoga to ap kahan karna chaengi?

Woman-06: mera to yahin ka khayal hai phir bhi kyu ke 7 mahine mein mujhey paani choot jata hai mujhey laga tha is baar nahi hoga asa lakin asa hi huwa tha

Woman-06: wo apko time dete (EDD) thy ke kab hoga bacha?

Woman-06: nahi time nahi dete thy din btaya tha lakin us se pehle hi bacha agya tha

Interviewer: or apki beti ki dafa mein asa huwa tha ya wo time par aen thin?

Woman-06: nahi wo bhi ase jaldi hi aaen thin

Interviewer: acha phir bhi apko abhi lagta hai ke hospital hi sahi hai?

Woman-06: jee hospital hi sahi lagta hai mujhey

Interviewer: kabhi apko kisi waja se laga ke apko ghar par hi bacha karna chaiye?

Woman-06: nahi asa nahi laga

Interviewer: is dafa majboori ki waja se karlya tha?

Woman-06: jee kyu ke 4 din se paani nikal raha tha

Interviewer: jab bacha nikal raha tha to hospital walon ko call ki thi?

Woman-06: jee ki thi phir gaari bheji thi unhon ne lakin bacha nikal gaya tha phir usey nehlay bager le gay thy phir bhi nahi rakha un logon ne

Woman-06: ap ne apni TBA jo nani hain apki unhain kab bulaya tha?

Woman-06: usi waqt

Interviewer: acha ghar mein kabhi kisi ke bachey TBA se huwe? kesi lagti hain ap ko TBA?

Woman-06: nahi kisi ne kabhi TBA se nahi karway KGH hi gay hain sab

Interviewer: sarey bachon ke waqt hospital mein acha tajruba raha hai? bas isi bachi ke waqt masla huwa?

Woman-06: jee bohat acha raha sab bachon mein bas isi mein masla huwa

Interviewer: ap bata sakti hain ke ghar par bacha karney ke kya nuqsanat hote hain?

Woman-06: nuqsanat to hain jese sahi se safai nahi ho jab ke hospital mein sahi se usi waqt safai kar dte hain

Interviewer: or koi nuqsanat ghar par delivery wagera ke waqt?

Woman-06: ghar par to nuqsanat hote hi hain abhi 4 din pehle bhi mujhey itna bara sa khachra nikla

Interviewer: apke ghar ke jo barey hain ya thy un ki kya soch hai ke ghar par thek hote hain ya hospital mein?

Woman-06: meri saas hi hain wo kheti hain ke bas hospital mein karna chaiye

Interviewer: apki raay mein delivery ke waqt kya masael aa saktey hain jin ki waja se hospital jana parta hai?

Woman-06: jee pet mein dard hota hai ghabrahat wagera

Interviewer: ap hamare center mein kitni dafa aaen hamal ke doran?

Woman-06: mahine mein bulate thy

Interviewer: apko center ki kya cheez sab se bhetar lagti hai?

Woman-06: sab kuch theek lagta hai mujhey ultrasound dawai wagera or sahi se pesh bhi aate hain

Interviewer: jab ap ne hospital mein bachey kye to apka tajaruba theek raha sab ache se pesh aaye?

Woman-06: jee jee sab theek tha ache se pesh aaye thy

Interviewer: hamal ke doran doctor ne koi mashwara dya tha apko ke kese apko apna khayal rakhna hai ya kuch samjhaya ke ase call kar ke gari bulwa saktey hain ap or sab

Woman-06: jee samjhaya tha lakin pheli dafa mein is dafa main nahi samjhaya kuch mein khud gai thi file bhi banwane

Woman-06: unhon ne kuch nuqsanat btay thy ghr mein karney ke jese khoon ziada nikal jata hai ya or kuch?

Woman-06: jee jee btaya tha khoon chute ya kuch bhi ho pet mein dard ho to aajana

Interviewer: apki sab normal delivery thin?

Woman-06: jee

Interviewer: koi cheez center ki apko achi na lagi ho?

Woman-06: nahi sab theek tha bas door bohat lagta hai kyu ke ab gari bhi nahi bhejtey lene baaqi sab theek tha

Interviewer: khane peeney ke baare mein batati thi wo ke ye khana wo khana?

Woman-06: jee

Interviewer: center ke ilawa ap kis jaghan ya kis insan par bahrosa rakhti hain?

Woman-06: family doctor

Interviewer: or koi hospital ya center jane mein rukawat?

Woman-06: bs fasley ka

Inrerviewer : or ap kis ke sath ati hain center?

Woman-06: akeli

Interviewer: or ap ke kabhi paise kharch huwe hain?

Woman-06: nahi bas is dafa kharcha hogya thora

Interviewer: acha to or koi cheezein asi jiski waja se ap center nahi ja paati ho koi asi rukawaten?

Woman-06: nahi

Interviewer: or ap ko center hi acha lagta hai or ap chaheingi ke apka next bacha bhi wahin ho?

Woman-06: jee center mein hi

Interviewer: or apki saas or ap ki raaye milti julti hi hai?

Woman-06: jee ak hi hai center mein hi

Interviewer: jee acha or koi asi baat jo ap batana chahein ya jo hum pooch na sakey ho

Woman-06: jee bas nahi mein chaati hun ke mere bachey ko ab jaongi to dekhainge ya nahi opd mein bulaya tha

Interviewer: jee jee hum unhain zaroor kahainge ke bachey ko bhi dekhain or gaari bhi bhijwani chaiye unhain. Bohat shukrya

____________________________X______________________________

IDI WOMAN-07

Interviewer: apki umar kya hai?

Woman-07: 20 saal

Interviewer: apki shadi ko kitna arsa hogya hai?

Woman-07: 3 saal

Interviewer: ap ke kitney bachey hain?

Woman-07: ye ak hi hai

Interviewer: ye ap ne ghar par kya hai?

Woman-07: jee

Interviewer: apki kitni taleem hai?

Woman-07: taleem to nahi hai

Interviewer: kuch kaam karti hain ap ya ghar par hoti hain?

Woman-07: ghar par hi hoti hun

Interviewer: apki raay mein bacha paida karney ke lye achi jaghan kon si hai or kyu?

Woman-07: sahi to hospital lagta hai lakin mujhey delivery ke dard raat mein huwe to phir mera bacha ghar par hi hogya tha TBA ko bula lya tha 10-15 minute mein bilkul sahi delivery hogai thi hospital jane mein bas ye masla aata ke hum ghar mein nand bhawaj hain bas to peechey ghar mein koi nahi hota agar donu chale jate to warna hospital mein bhi koi masla nahi tha lakin ghar mein hogya tha irada lakin hospital ka hi tha unhon ne 8 tareekh di thi lakin 3 din phele hi hogya phr do din bad center wale ay thy bachey or maa ko lene

Interviewer: apki raay mein ghar mein bacha paida karney ki kya saholiyaat hoti hain?

Woman-07: hospital mein mene pehlabacha kya tha lakin wahan mujhey kisi ne btaya nahi tha kuch lakin yahan ghar mein meri nand nei sab btaya tha kya kya masley hote hain to mujhey asani huwi

Interviewer: TBA ke sath ap ka tajruba kesa raha ?

Woman-07: TBA ne mujhey injection lagaya phele mujhey dard nahi tha phir mujhey dard huwe or bacha hogya aram se pyar se baat ki or sab samjhaya mujhey unho ne ke ye ye hoga sab

Interviewer: TBA ke sath apka tajruba theek raha ?

Woman-07: jee , hospital walon ne pheli dafa mein mujhey kuch nahi samjhaya tha ke dard aenge mujhey pata bhi nahi tha pehlabacha tha mera or taankey bhi ay mujhey pheli dafa mein center mein lakin is dafa TBA ne mujhey sab samjhaya or taankey bhi nahi islye mujhey hospital se bhetar ghar laga

Interviewer: ap ke 2 bachey hain?

Woman-07: meri pheli shaadi se woh bacha tha jo hospital mein kya tha us shohar ne mujhey talaq dedi thi or is shohar se mujhey ye pehlabacha hai jo ghar mein huwa hai

Interviewer: ghar mein karney se apko koi nuqsanat lagey?

Woman-07: jee nahi mujhey to faida hi huwa hai ghar mein

Interviewer: apka pehla bacha normal tha?

Woman-07: jeet ha to normal lakin bachey ka sir bara tha to machine se nikala tha islye mujhey tankey ay thy

Interviewer: bacha bilkul sahi tha?

Woman-07: nahi bachey mein paani chala gya tha usey 2 din machine mein bhi rakha tha phir sahi hogya tha

Interviewer: ye dusra bacha apka normal hai?

Woman-07: jee ye normal hai

Interviewer: ghar par apko ziada aram or dekh bhaal lag rahi thi TBA ke sath?

Woman-07: jee sab aram se hogya tha

Interviewer: ap ke baron se kabhi kuch asa suna ke bacha paida karney ke lye kya jaghan bhetar hai apko kya samjhaya unho ne?

Woman-07: sab khate hain ghar mein acha hai baaqi ye hota hai ke center mein 2 se ziada ladies nahi reh sakti ab hum hamla aurat ke pass honge usey aram se samjhaenge lakin woh rukney nahi dete ke hum doctor hain ya ap to ye baten achi nahi lagti

Interviewer: apko ghar mein nacha karney ke koi nuqsanat lagtey hain?

Woman-07: nahi

Interviewer: hospital mein paida karney se kya nuqsan lagte hain?

Woman-07: ak to tankey baaqi bachey ki fikar nahi hoti wahan bhi Allah sab jaghan hai normal wahan bhi hojata hai yahan bhi operation wahan bhi hojata hai yahan bhi lakin wahan bas ye cheez pareshan karti hai k 1 se 2 aurton ki zaroorat par jay to woh humey ghusney nahi deti andar , mene bahar se karwaya tha ultrasound to mujhey pure din btaya thy 11 mahiney batay thy or mein center mein btati thi to woh kehte thy tum jhoot bolti ho abhi time hai abhi jao or tareekh par tareekh dete rhete thy woh

Interviewer: ye btaen rukhsana jab ap center aati thin to hamal ke doran kitni dafa gaen ?

Woman-07: jab jab tareekh dete the

Interviewer: apko center ki cheezen wahan ka sab kuch kesa lagta hai ?

Woman-07: jab mene ultrasound karwaya center mein to bachey ki theli nahi arh thi us par lakin mujhey unhon ne kuch nahi btaya jab ke unhain batana chaiye tha ke ye ye masla aa raha hai bachey ke sath ye mujhey TBA ne btaya delivery ke time

Interviewer: apka center ke sath tajruba kesa raha wahan ki kya saholat sab se achi lagi?

Woman-07: ziada kuch nahi bs mujhey karwana to wahin tha lakin bas Allah ki taraf se gahr mein hogya

Interviewer: to center wale apko bilkul kuch guide nahi kartey thy?

Woman-07: nahi

Interviewer: apko kis ne sahi btaya center walon ne ya jin doctor ne ultrasound mein 11 mahine btay thy unhon ne?

Woman-07: ultrasound walon ne center walon ne to mujhey kaha ke abhi bhi apko time hai abhi ap chali jao

Interviewer: ap ke khayal mein kis waqt or kis masael ki waja se hospital jana parta hai?

Woman-07: phele mein kehtithi hospital sahih ai TBA nahi lakin sab barey kehte thy ke TBA sahih ai lakin jab mere phele bachey mein mujhey tajruba huwa to ab mein kehtihunk e TBA theek hai hospital sahi nahi lagta mujhey ab

Interviewer: center walon ne apko kabhi kuch nahi smjhay ke ap ke liye kya sahi kya galat hai hamal ke doran ya bachey ki kya condition hai ?

Woman-07: nahi puchney par bhi nahi btaya

Interviewer: ap TBA ko kese janti hain?

Woman-07: paros mein hain

Interviewer: to ap ke taaluq un se phele se hain?

Woman-07: jee

Interviewer: or TBA koi fees leti hain?

Woman-07: jee 4000 Rs larkey ke or larki paida ho to 3000 Rs

Interviewer: center mai to free mein hota hai phir bhi ap TBA se sahi kehtihain?

Woman-07: jee , kyu ke mera tajruba acha nahi raha hospital mein

Interviewer: or koi rukawaten ya wujoohat thin ghar mein karney se?

Woman-07: nahi or kuch nahi gari walon ki bhi itni minatten karti thi lakin gari nahi aati thi mere sath meri nand paidal jatey thy hum

Interviewer: aap ka kharcha huwa apka hamal ke doran

Woman-07: center wale mujhey bahar ki hi dawai dete thy toh woh khud leti thi mein kabhi hoti thi un ke pas to de dete thy warna kehte thy bahar se lo taqat wagera ki dawai hoti thi woh

Woman-07: or koi dusri rukawat hospital mein na karney ki?

Woman-07: jee bas pehlat tajaruba bhi sahi tha or unhon ne sahi se samjhaya bhi nahi mujhey

Interviewer: abhi ap jati hain center? bachey ke lye?

Woman-07: nahi abhi nahi jati bachey ke lye bhi nahi jati agar bacha bemar hota hai to kahin or se dawai le aati hun center se mera dil kharab hogya hai ab

Interviewer: us mein kitna kharcha lagta hai?

Woman-07: ziada nahi bs 500 tak center se koi aata bhi hai to hum mana kar dete hain unhain

Interviewer: kabhi kisi ko center ki complain ki hai btaya hai ke mujhey yahan se ye masael hai?

Woman-07: nahi kisi se nahi

Interviewer: phele bachey ke doran center mein apko kya kehte thy?

Woman-07: phele bachey ko center mein kehte thy isey koi bari bemari hai isey sirf maa ka doodh do or kuch nahi lakin maa ka doodh hi nahi de sakti thi to kya deti usey attia hospital bhi bheja tha lakin woh to bilkul theek tha kuch nahi tha usey

Interviewer: attiya mein sahi huwa tha ilaaj unka?

Woman-07: bas attiya mein doctor ne dawai di or woh sahi hogya kahin or nahi le jana para bad mein jahtkey parey tw hum ne center call ki lakin unhon ne nahi uthai phir hum apne kharchey par JPMC le gay thy usey

Interviewer: phele bachey mein apka center mein tajruba kesa raha

Woman-07: jab bhi unhon ne kuch btaya hi nahi tha

Interviewer: us ke baad bhi ap hr mahine center mein kyu jati thi ap kis waja se?

Woman-07: phele staff kuch or tha mene socha ab change hogya hoga staff to acha hoga lakin abhi bhi koi acha nahi hai koi kuch nahi samjhata na ache se bat karta hai

Interviewer: ap ne kabhi kisi or se suna center ke bare mein kuch acha ya bura?

Woman-07: meri parosan or sab wahan jate to hain naam bhi likhwate hain lakin delivery ghar hi kartey hain pata nahi kyu kabhi kisi ne btaya nahi bas woh log center dawaai wagera ki waja se jate hain ke wahan se free mein milti hai baqi yahan ak TBA hain sab ki delivery who hi karti hain

Interviewer: TBA ka naam kya hai?

Woman-07: TBA naam se mashoor hain asal naam nahi pata or ziada masley par woh bhi kehti hain ke doctor ke le jao

Interviewer: ap kisi ase ko janti hain jinhon ne hospital mein kya ho bacha?

Woman-07: nahi, pathan or Baloch jo hain woh kehte hain yahan ke humey TBA par bharosa nahi hum hospital hi jate hain baaqi hamare goth mein sab ghar mein karwatey hain islye mujhey nahi pata

Interviewer: theek hai jee bohat shukriya

IDI WOMAN-08

Interviewer: ap kitne saal ki hain?

Woman-08: 40 saal

Interviewer: apki shadi ko kitna time hogya?

Woman-08: 14 saal

Woman-08: ap ke kitne bachey hain?

Woman-08: 4 thy ak pehli beti fout hogayi thi

Interviewer: ap ke mujooda hamal ki delivery kahan huyi?

Woman-08: waise to naam likhwaya tha center main gai bhi thi lekin barish bohat thi to ghar wapis aagaye thy phir dard bohat thy to raat ko 2 bajey ghar mein hogya tha

Interviewer: ap ki taleem kya hai?

Woman-08: nahi ki hai taleem

Interviewer: ap kuch kaam karti hain?

Woman-08: nahi ghar hi hote hain

Interviewer: ap ke khayal main bachey paida karney ke liye bhetar jaghan konsi hai?

Woman-08: mere khayal se to KGH hai lakin mere bachey ghar main hi huye hain

Interviewer: apko hospital kyun behtar lagta hai?

Woman-08: ghar main gandagi hoti hai hospital main saaf suthra kar dete hain

Interviewer: us ke bawajood ap ke 3 bachey ghar par hi kyun huye hain?

Woman-08: bas inki daadi hi TBA thi to un se hi huye hain

Interviewer: kya waja thi koi rukawat thi hospital ke liye ya ap shuru se hi chah rahi thin ke ghar par hi ho?

Woman-08: jee mein chahti thi ghar par hi ho

Interviewer: ghar par bacha karney ka kya faida hai?

Woman-08: bas parda rehta hai ghar par kisi ko pata bhi nahi chalta warna hospital aane jane main sabko pata lag jata hai

Interviewer: acha or koi nuqsanat nazar aatey hain apko ghar main karney ke?

Woman-08: jee nahi

Interviewer: ap ke bachey normal huye thy koi mushkil nahi aai thi?

Woman-08: jee normal huye thy koi mushkil nahi aai thi

Interviewer: kabhi apne ghar ke baron se baat ki hai is hawaley se ke un ke tajarube kaise rahe hain bachey paida karney main?

Woman-08: jee meri Bhabhi or behan dono ne hospital mein hi kiye hain bachey sirf mere ghar par huye hain

Interviewer: unhon ne kuch apna tajaruba bataya?

Woman-08: jee un se pata laga ke hospital bhi zaroori hai

Interviewer: to ap ghar ko tarjeeh nahi deti hain us ke bawajud ghar main bacha paida karney ki waja? aesa kya acha lagta hai apko ghar main?

Woman-08: hospital main paison ka hota hai mere shohar ka kaam kabhi hota hai kabhi nahi hota islye ghar main hi behtar lagta hai

Interviewer: ap ke khayal main sehat ke hawaley se wo konse halaat hain jin main hospital jana zaroori hota hai?

Woman-08: BP high ya low hojana or tabiat k hawaley se masaail ho saktey hain

Interviewer: ap ke sath huwa kabhi kuch aesa?

Woman-08: nahi

Interviewer: ap hamarey center jaati thin hamal ke doran?

Woman-08: jee file bhi pari huyi hai

Interviewer: wahan doctor ya midwife wagera ap se bachey ke hawaley se hamal ya delivery ke hawaley se baat karti thin?

Woman-08: jee

Interviewer: ap mujhey bata sakti hain ke kya baat hoti thi?

Woman-08: bachey ke hawaley se khayal rakhna or BP wagera ka khayal rakhna baaqi emergency ho to call karna number dya tha

Interviewer: kabhi delivery ke hawaley se baat ki thi k kahan hona chaiye bacha?

Woman-08: nahi bas tareekh di thi main gai thi to barish thi islye main wapis aagai thi

Interviewer: kitni dafa jati thin center hamal ke doran?

Woman-08: jo tareekh wo dete thy un main jese 18 ho 19 ho ya 20 wagera to un days main

Interviewer: hamarey center main apko sab se achi cheez kya lagi thi?

Woman-08: gaari ki saholat ke ghar se aa kar le kar jati thi

Interviewer: baaqi kya apko dawai milti thi or ultrasound hote thy?

Woman-08: jee sab hota tha

Interviewer: is ke bawajud ap ne ghar main kye bachey to center main kuch aesi kami jo apko mehsoos huyi ho?

Woman-08: nahi bas barish ki waja se din main bhi doctors chale gaye thy to maine socha raat main bhi na ho shayad lekin unhon ne kaha ke emergency ke liye gaari or doctor mujood hote hain lekin raat main maine khud hi phone nahi kya tha

Interviewer: to ap chah rahin thin ke hospital main hi ho?

Woman-08: jee main chati thi hospital main hi ho

Interviewer: jo bachey ap ke pehle ghar par huye unka tajaruba kaisa tha?

Woman-08: wo bhi hogaye thy normal maine kisi ko kabhi bataya bhi nahi ke bemaar hun ya kuch bhi or un dafa main center bhi nahi gai thi main bas isi dafa main gai thi

Interviewer: is dafa main kis waja se gai thin?

Woman-08: bas mein ghar par akeli thi to socha nahi ho paayega ghar par islye lekin ghar par hi hogya phir barish ki waja se

Interviewer: apki saas TBA hain?

Woman-08: jee mere bachey unhi ke pas huye hain

Interviewer: pehle bachon main ap nahi gayin center lekin is dafa gayin to sab se achi kya cheez lagi apko wahan?

Woman-08: gaari or ultrasound dawaai wagera

Interviewer: baaqi test wagera jo wo karwatey thy wo theek lagtey thy apko?

Woman-08: jee theek lagtey thy

Interviewer: or jis tarhan se wo ap se baat kartey thy apko sab sahi se samajh aata tha?

Woman-08: jee bilkul sab sahi se samajh aata tha

Interviewer: kabhi danttey thy ya har sawal ka jawab wo deite thy?

Woman-08: jee deite thy or daanttey bhi nahi thy

Interviewer: ap ke ghar main faisley karney wala kon hai?

Woman-08: mere saas susar

Interviewer: kya apko lagta hai ke un ke khayalat bachey ki paidaaish sei mutaliq ap ke khayal se mukhtalif hain?

Woman-08: nahi ak jese hain wo bhi kehte hain ke takleef ho to hospital jao warna ghar bhi theek hai

Interviewer: center walon ne apko bataya tha ke hospital jana kyu zaroori hota hai?

Woman-08: jee bataya tha ke jab bemaar ho to phone kar ke gaari bulwao hospital ke lye

Interviewer: ap hospital ke ilawa apni delivery ke liye kis par ziada bharosa karti hain?

Woman-08: meri saas jo TBA bhi hain

Interviewer: ap ghar ko ziada pasand karti hain ya hospital

Woman-08: ghar ko

Interviewer: ap hospital gayin hain kabhi?

Woman-08: apney liye nahi gayi kisi ke sath gayi

Interviewer: acha jo ap ne wahan dekha wahan ka tajaruba kaisa raha?

Woman-08: sahi raha

Interviewer: ap ne kisi ka suna hai jis ne hospital main bacha paida kiya ho, unka tajaruba kaisa raha?

Woman-08: jee sahi raha

Interviewer: ap ne phir ghar main kin saholiyaat ki waja se bacha kiya?

Woman-08: idher to ghar main kuch bhi nahi warna hospital hi sahi hai

Interviewer: center walon ne ye bataya tha ke ap phone kar sktey hain hospital

Woman-08: jee sab bataya tha ke ap call kar ke bula saktey hain

Interviewer: lekin ap phir bhi chahti hain ke ghar par ho

Woman-08: jee ghar par ho

Interviewer: jese ke ap ke phele bachey bhi ghar main huye or abhi bhi or us waqt ap ki saas ap ke pas hoti hain to wo kya karti hain aesa ya kya cheezen apko us waqt achi lagti hain ghar par?

Woman-08: sath hoti hain to dil bhi sukoon main rheta hai ke bari hain mere saath or taaqat ke liye bhi kuch de deti hain

Interviewer: center janey main to koi masla nahi hota apko?

Woman-08: nahi

Interviewer: ap ke saath kon jata tha?

Woman-08: yahan se akele jati thi

Interviewer: ap ne kabhi hamal ke doran koi kharcha huwa hai?

Woman-08: kabhi doodh fruit seib wagera khane peene par

Interviewer: is ke ilawa koi dawaai wagera par koi kharcha?

Woman-08: jee nahi

Woman-08: ghar par ap ko kya cheez eham lagti hai?

Woman-08: ammi ki waja se kyu ke wohi TBA hoti hain

Interviewer: aesi koi or waja jis ki waja se ap hospital jana pasand nahi karti?

Woman-08: nahi

Interviewer: koi aesi cheez jiski waja se apko lagta hai ke hospital jana hai?

Woman-08: BP wagera

Interviewer: aap ke sath raha hai koi masla aesa?

Woman-08: jee nahi

Interviewer: hamare center main sab baat kesay kartey hain ap se?

Woman-08: jee jee...theek sei

Interviewer: koi aesi cheez jo hum center main change karna chahein ya behtar kar sakein?

Woman-08: or to kuch nahi sab behtar hi hai baaqi to

Interviewer: jab apka phela bacha huwa tha tab bhi ap ghar par karna chahti thi?

Woman-08: jee TBA se ghar par hi huyi thi pehli beti

Interviewer: or koi sawal ya aesi baat jo ap batana chahein or hum na pooch sakein ho?

Woman-08: jee nahi bas

Interviewer: okay bohat bohat shukrya apka…………………………..

_____________________________X_______________________________

IDI Woman-09

Did not consent to an audio recording.

Abbreviations Used Below

- PHC: *Primary Health Center*
- ANC: *Antenatal Care*
- TBA: *Traditional Birth Attendant*

SD Questions

- 21 years old.
- Married for 4 years.
- 2 children. Her first child was born at a secondary care facility while the second child was born at home.
- No formal education.
- Homemaker.

Questionnaire

- **Where does she prefer to give birth?**
  - Believes that the hospital is a "safer place" for birth as emergencies and complications are difficult to resolve at home.
  - Registered herself for antenatal care at the PHC during her second pregnancy. She was attending her ANC visits as scheduled.
  - She went into labor during the month of Ramadan. Her family members were fasting, and she thought they could go take her to the hospital after they broke their fast. However, her labor progressed quickly and there was not enough time to go to the hospital for delivery.
- **Did the midwife at the PHC tell her about her EDD?**
  - Yes, her EDD was written on her antenatal file, but her baby came 2-3 days earlier.
- **Did she call the PHC to request transport for a facility delivery?**
  - She did not call the PHC to request transport for delivery when her water broke. Although they had counseled her on birth preparedness, she mentioned that she did not think to call while she was in labor (given its pain intensity).
- **Did she call anyone to assist with the birth?**
  - Her mother-in-law's mother-in-law (a former TBA) helped deliver the baby at home. They did not feel the need to call anyone else—however, she also mentioned that her father-in-law did not allow them to call anyone else to their home to assist with delivery.
  - She had a normal delivery at home.
- **What is her mother-in-law's view about the choice of birthing location?**
  - Her mother-in-law believes that women should give birth at home. However, she laments that times are changing and women these days are "weaker" and "prone to more illnesses."
- **Under what circumstances does she feel it is necessary to go to a hospital?**
  - When you feel sick (*"pata chal jata hai"*).

    - After probing her about her antenatal counselling, she remembered the midwife at the PHC telling her about danger signs such as bleeding or breech position.
- **What has her experience with ANC (at the PHC) been like?**
    - She mentioned that she liked receiving free check-ups, tests, and medicines.
    - Although the waiting time at the PHC was often long (typically an hour long), the health workers were approachable and respectful in her experience.
- **What was her first childbirth experience like (at a secondary care facility)?**
    - It was a normal delivery.
    - She mentioned that she received stitches (following an episiotomy).
    - She fainted from blood loss.
    - Her experience did not scare her or shake her faith in facility-based care – she made peace with it, believing that the blood loss (and resultant fainting spell) was a matter of fate ("*bemaari meri kismat mein thi*").
    - Mixed experiences with the staff at the secondary care facility. She agreed that she received timely care but felt that there was a lack of patient involvement. She mentioned that there was a tendency to minimize her pain, along with a lack of debriefing of what was happening/going to happen to her as she progressed in her labor.
- **Who else does she trust when it comes to her pregnancy and intrapartum care?**
    - Above all, God is responsible for a safe pregnancy and delivery – "*Allah paida karta hai…lekin apnay ap ko mehfooz rakhna zaroori hai"*
    - She trusts trained midwives and doctors (facility-based care) – "*We are poor…who can pay for an ultrasound twice a month? If we are getting free services at a facility, we would be foolish not to avail it."'*
- **Are there any barriers that prevent her from seeking formal healthcare?**
    - No. Distance is not a problem.
    - Some women prefer the home because it allows them to be surrounded by their loved ones, creating a safe environment for birth. However, she mentions that she would prefer not to have her family with her during delivery – "*Family dard nahi kam karsakti"*

____________________________X______________________________

IDI WOMAN-10

Interviewer: apki shadi ko kitna time hogya?

Woman-10: 5 saal

Interviewer: or ap ke bachey kitney hain?

Woman-10: 2

Interviewer: or abhi jo bacha huwa hai wo ghar par huwa hai ap ke?

Woman-10: han

Interviewer: or phela bacha kahan huwa tha?

Woman-10: wo Aisha Hospital Gulshan e Hadeed main

Interviewer: apki taleem kya hai?

Woman-10: nahi kuch nahi hai

Interviewer: ap kuch kaam karti hain ya ghar par hi hoti hain?

Woman-10: ghar ke hi kam karti hun

Interviewer: ap ke khayal main bacha paida karney ke lye bhetreen jaghan kya hai?

Woman-10: ghar

Interviewer: kyun?

Woman-10 : hospital main sharam aati hai is lye ghar main theek rehta hai

Interviewer: kis tarhan se sharam aati hai.

Woman-10: bas wahan wo chillatey hain idher jao udhar jao ghar main ye sab nahi hota

Interviewer: ap ke sath aesa huwa hai hospital main ke kabhi kisi ne danta ho ya chillaya ho?

Woman-10: nahi mere sath nahi huwa

Interviewer: phir ap ne kisi ka suna ke kisi ke sath aesa huwa ho?

Woman-10: nahi

Interviewer: to phir ap ko kese pata laga ke wahan chillatey hain danttey hain

Interviewer: bas wahan na chalatey bohat hain idher se udhar

Interviewer: or ghar par apko kya acha lagta hai?

Woman-10: ghar par maa sath main hoti hai or to bas

Interviewer: ghar main bacha paida karney ka kya faida hota hai?

Woman-10: faida ye ke koi dekhney wala nahi hota hum ne doctor ko bulaya tha wo ghar main delivery karti hain Ibrahim haidry main hoti hain

Interviewer: apko lagta hai ghar main karney ke kuch nuqsanat bhi hain or kya?

Woman-10 :jee ghar main garmi bohat hoti hai light kabhi hoti hai kabhi nahi ye masla ghar par hota hai

Interviewer: or koi sehat ke hawaley se nuqsanaat?

Woman-10 : bas garmi ki hi waja se ghabrahat hoti hai aesa lagta hai bas aj main mar jaongi

Interviewer: ap ke ghar main jo barey hain unki bachey ki pedaaish ke hawaley se konsi jaghan behtar hai?

Woman-10: phele sab ghar boltey the lakin ab hospital khete hain ke ghar main ab khatra ho sakta hai

Interviewer: apka irada kahan karney ka tha?

Woman-10: phele mera bacha hospital main huwa tha to main dar gai thi hospital se

Interviewer: kyu kya huwa tha aesa

Woman-10: bas phele bachey main pata nahi hota na to main bas pure hospital main bhaag rahi thi idher se udhar ke mujhey ghar jana hai main bas dard jo tez tez ho rahe the us se dar gai thi

Interviewer: unhon ne aesa koi ilaaj ya asi koi bat ki thi jiski waja se ap dar gaen thin?

Woman-10: nahi nahi sab theek tha bas udhar janey main ghabrahat ho rahi thi wo kabhi injection laga rahe hain kabhi drip laga rahe hain ase kabhi phele huwa nahi tha to ghabrahat ho rahi thi

Interviewer: to wo apko batate nahi the ke ye sab kyu kar rahe hain kyu laga rahe hain?

Woman-10: jee nahi us se bhi ghabrahat horh thi phir na ammi bhain koi pas nahi tha sab alag the islye bhi

Interviewer: ase kon se halat mushkilaat hoti hain jinki waja se apko lagta hai ke hospital jana zaroori ho jata hai?

Woman-10: ghar par ye hai ke hospital tak paidal jana parta hai

Interviewer: or koi sehat ke maesael jiski waja se hospital jana zaroori lagta ho?

Woman-10: hospital main ye hai ke dawai wagera mil jati hai jo masla ho ghar main dawai nahi mil pati jese mere phele bachey main mujhey dawai calcium ke sachet sab miley the

Interviewer: or ap hamarey center par aen thin?

Woman-10: jee 2 3 dafa aai thi abhi is wale bachey main bhi 2 dafa aai thi ak dafa file bani thi meri or phir dusri dafa

Interviewer: to ap sirf 2 3 dafa itna kam kyu gaen?

Woman-10: ghar main kaam hota hai na mene wahan bhi kaha tha ke khana wana main hi banati hun or ghar ke kaam

Interviewer: or to koi waja nahi thi rastey ka masla faasla ziada tha ya kuch or?

Woman-10: nahi bas fursat nahi mil pati thi

Interviewer: or koi rukawaten thin?

Woman-10: nahi

Interviewer: or center jati thin ap to center kesa laga apko?

Woman-10: acha tha bas wahan time bohat lagatey hain

Interviewer: or unka ilaaj ya bat karney ka andaaz sab sahi tha apko sab kuch sahi se samjha rahe the?

Woman-10: jee sab sahi tha

Interviewer: jab ap jati thin to kya kya cheezen btate the apko wahan?

Woman-10: bas ultrasound karti thin or kharey kar ke measurements wagera

Interviewer: apko aesa kuch btaya tha ke ap ke sath kya kya maesael ho saktey hain jinki waja se apka hospital aana zaroori ho sakta hai?

Woman-10: jee btati thin ke koi takleef ya bemaari ho to foran dekhane aana, dawai time par lena or gutka chor do ab , main gutka khati hun na to mana kya tha ke chor do ab phir main dawai lene gai hi nahi

Interviewer: koi waja jiski waja se ap ja nahi saki thin?

Woman-10: waja ki nahi bas mujhey time nahi milta tha jab milta tha to m jati thi

Interviewer: or kuch samjhaya tha unhon ne ke bacha kabhi ulta bhi ho skta hai ya kabhi khoon ziada bhi aa sakta hai?

Woman-10: jee jab main phele bachey main test karwane gai thi tab mujhey bola tha ke apka bacha ulta hai lakin abhi time hai to time par sahi hojaega

Interviewer: agar apka dusra bacha bhi ulta hota to ap hospital jati ya ghar par hi karti?

Woman-10: main hospital jati

Interviewer: center main kuch aesa huwa jiski waja se ap ne socha ke mujhey hospital main nahi karna?

Woman-10: nahi

Interviewer: akhri waqt tak apko bacha ghar par hi karna tha?

Woman-10: jee

Interviewer: phir ap ne ghar par TBA ko bulaya tha? kesa ilaaj kya tha TBA ne?

Woman-10: TBA ne goliya di phele check kya phir kaha khana kha kar ye goliya lo us waqt khana to mujhey acha nahi lag raha tha dard bohat tha phir mene goli khai unhon ne kaha 3 bajey le kara ana isey mene goli khaai thi to dard mujhey or ziaxcda hogya main 3 baje gai wahan phir 4 bajey hogya

Interviewer: unhon ne btaya tha ke goliya kyu di thi?

Woman-10: nahi bas kaha tha ke dard ziada hunge

Interviewer: apka phele bachey ka hospital main tajruba kesa raha?

Woman-10: wo acha tha ghar main takleef bohat huwi or garmi bhi hospital main ziada acha raha bas wahan kisi ko andar nahi aane dete pas ammi bhaino wagera ko

Interviewer: agla bacha apka ap kya chati hain kahan ho?

Woman-10: dekhainge halat ache huwe to hospital chale jaenge

Interviewer: halat kis waja se?

Woman-10: main pese jama karti hun na jab prt se hojati hun to bchey ke liye pese jama karti hun

Interviewer: ap ke ghar ke faisley kon karta hai?

Woman-10: nani

Interviewer: unkey or ap ke khayal ak hain bachey ki pedaaish ki jagha ke mutaliq

Woman-10: nahi wo to meri marzi hoti hai wo kuch nahi kheti lakin unhon ne ghar par kye hain to ghar hi kheti thin lakin mujh par chor dya tha phir

Interviewer: center or hospital ke ilawa ap kis par bharosa karti hain delivery ke liye?

Woman-10: hospital par hi

Interviewer: apko kabhi hamare center aane main koi masla to nahi hota faasley wagera ka?

Woman-10: nahi koi masla nahi hota aesani se poanch jati hun

Interviewer :or kis ke sath jati hain ap wahan ap?

Woman-10: nand ke saath

Interviewer: or ilaaj ke liye ya delivery ke liye kabhi pese kharch karney pare hain apko?

Woman-10: nahi

Interviewer: ap ne jab Aisha Hospital par kya tha us main kitney pese lagey the apke?

Woman-10: 30 hazar

Interviewer: ye to bohat hain acha ap ko hamarey center par bataya gaya tha ke wahan sab free main hota hai?

Woman-10 :jee bataya tha

Interviewer: unhon ne apko bataya tha ke gari aaya karegi ye sab?

Woman-10: jee bataya tha ke gaari aegi apko khud lene sab lakin mainne kaha mujhey dar lagta hai yahan karwane se

Interviewer: to Aisha Hospital main apko aesa kya sahi laga?

Woman-10: wo to achanak dard hogay to hospital le gay meri saas ne aisha hospital main kya tha islye wahan le gay

Interviewer: or koi rukawaten hain jinki waja se ap center nahi pati?

Woman-10: nahi or koi rukawat nahi hai
Interviewer: ap ye bataen apko ghabrahat kis waja se hoti hai hospital main?

Woman-10: han dard hota hai ghar par ye hota hai jese dard hota hai wese teeka lagate rhete is baar ghar par mujhey 4 teekey lagey the ghar par upper se garmi iti or wese main to paani bhi nahi de saktey na ke khete hain kuch nahi dya jata us waqt

Interviewer: to ap mawazna kar sakti hain kon si jagah ziada behtar hai?

Woman-10: hospital

Interviewer: kya ap batana chaheingi kuch aesa jo hum hospital ya center main tabdeeli la saktein hain kya bhetri akr saktey hain?

Woman-10: bas wahan 2 log ho ziada na ho ak doctor ak nurse or mareez agar bole to ammi ya behan wagera ko aane den hospital main bas alag se kamra ho ak nurse or ak doctor bas

Interviewer: or kuch aesa jo ap batana cha rahin hun ya hum pooch na sakein ho?

Woman-10: nahi bas yehi jo mene kaha

Interviewer: okay bohat shukrya

___________________________X_____________________________

IDI WOMAN-11

Interviewer: apki umar kitni hai?

Woman-11: 25

Interviewer: apki shadi ko kitna time huwa hai?

Woman-11: 3 saal

Interviewer: ap k kitney bachey hain?

Woman-11: 2

Interviewer: ap ne phela bacha kahan kya tha?

Woman-11: ghar par

Interviewer: ap ne taleem kitni hasil ki hai?

Woman-11: 8 class

Interviewer: ap kahin kam karti hai ya ghar par hi hoti hain?

Woman-11: ghar par hi hoti hun

Interviewer: bachey paida karney ke liye ap ki nazar main behtreen jagah konsi hai?

Woman-11: mene to hamesha ghar main hi kya hai is liye mujhey to ghar main hi bhetar lagta hai

Interviewer: or ghar main paida karney ke kya faidey lagtey hain apko?

Woman-11: mere ghar wale mere pas hote hain

Interviewer: or ghar par paida karney ke koi nuqsanaat lagtey hain apko?

Woman-11: han nuqsan bhi ho saktey hain jese takleef ziada hoti hai zor laganey main to nuqsaan ho sakta hai hospital bhi sahi hai mene kabhi kya nahi hai bacha hospital mail akin mujhey lagta hai ghar se ziada hospital sahi hai

Interviewer: ap ka irada tha hospital main karney ka?

Woman-11: han han irada tha lakin itna time nahi mila jaldi jaldi m ho gaya

Interviewer: or phele bachey ki dafa main bhi apka irada hospital main tha?

Woman-11: nahi tab ghar main hi karna tha

Interviewer: ap ke ghar ke barey kya khete hain hospital sahi hai ya ghar?

Woman-11: hospital

Interviewer: or apka khayal bhi un ke jesa hai ya alag?

Woman-11: nahi ak jesa hi hai

Interviewer: acha ap hamarey center par aati thin hamal ke doran? or kitni dafa aaen?

Woman-11: 2 dafa aai thi phir nahi aai phele bachey main bhi 2 dafa aai thi

Interviewer: 2 dafa hi kyu or mazed bad main kyun nahi thin?

Woman-11: kyu ke mujhey lagta tha ke main thyek hun mujhey asi koi takleef nahi hoti thi ke mujhey jana parey

Interviewer: center ki sab se behtreen cheez kya lagti hai apko?

Woman-11: wo ultrasound kartey hain khon test wagera hota hai to ye sab acha lagta hai wahan ka pata chalta hai ke main or bacha sahih ai

Interviewer: center ke barey main kuch asa jo apko sahi nahi lagta ya jo hum bhetar kar saken?

Woman-11: nahi kuch nahi

Interviewer: acha hospital ke bare main ap ke kya khayalat hain?

Woman-11: ase kuch khayal nahi kyu ke mene khi hospital main delivery ki nahi hai isliye mujhey nahi pata kya hota hai wahan kya nahi bas ziada hospital bhi mujhey thyek nahi lagta

Interviewer: hospital ke barey main ap ne kuch suna hai ya jesa ap ne kaha hospital apko thyek lagta hai to asa kya hai jiski waja se ap hospital nahi jaengi?

Woman-11: jee kuch log hai hamare jinho ne hospital main delivery ki hai to kuch log khete hain hospital thyek nahi hai wahan ki nurse wagera or kuch khete hain k thyek hai

Interviewer: ziada tar kya sunti hain ke sahi hai ya sahi nahi hai?

Woman-11: main ziada tar ye sunti hun ke sahi hai hospital main delivery

Interviewer: or jo log khete thy sahi nahi hai wo kis waja se khete thy k kya sahi nahi hai?

Woman-11: wahan ki jo sisters hain jo delivery karwati hain wo ziada tar takleef deti hain larti hain dantti hain k ase ase ase sab

Interviewer: jab ap center jai thin to wo sahi se bat kartey thy ap se or sahi se cheezen sab samjhatey thy dawai wagera sab? kabhi ye bataya ke delivery ke doran apko kya kya takleef ho sakti hain?

Woman-11: jee sahi se bat kartey thy

Interviewer: ye bataya kabhi k delivery main kya kya masley aa saktey khoon ziada nikal jana ya kuch?

Woman-11: nahi asa to kuch nahi samjhaya kabhi mujhey

Interviewer: wo shayad is liye ke ap 2 dafa hi gaen thin na , 2 dafa hamal ke kis doran gaen thin?

Woman-11: pheli dafa 4 month main or ak 5 month main

Interviewer: apko kya lagta hai delivery ke doran kon se masael ki waja se hospital jana zaroori hota hai?

Woman-11: paani kam hota hai to delivery ke doran takleef ziada hoti hai is waja se

Interviewer: ap ke bachey normal huwe hain?

Woman-11: ap ke saath ya bachon ke sath koi masla to nahi huwa tha?

Woman-11: nahi Allah ka shukar koi masla nahi huwa tha

Interviewer: to delivery ke waqt TBA aen thin?

Woman-11: jee

Interviewer: un ke sath apka tajruba kea raha?

Woman-11: sahi tha wo bhi sahi karwati hain achi tarhan se

Interviewer: unhon ne kese ilaaj kya apka sab cheezen kese ki?

Woman-11: in sab ka to pata nahi us waqt takleef itni hoti hai ke kuch samajh nahi aa raha hota
Interviewer: acha ap mawazna ka sakti hain TBA ke sath or hospital ke bare main?

Woman-11: hospital main to mene suna hai andar ki safai ache se hojati hai TBA to apne tareeqe se karti hai isliye TBA par bhi ziada bhrsosa nahi ke us ne safai thyek se ki hai ya nahi

Interviewer: to ap ka achanak ho gaya tha bacha center walon ne ultrasound main bataya nahi tha ke kab tak hoga apka bacha kis time ya kuch?

Woman-11: nahi kuch nahi bataya tha

Interviewer: or koi waja jiski waja se ap center nahi ja rahin thin fasley ka masla ya kuch or?

Woman-11: nahi koi masla nahi tha bas mujhey lag raha tha ke main thyek hun koi takleef nahi thi bas islye nahi gaai

Interviewer: delivery ka waqt jab qareeb tha tab ap ne center par call ki thi?

Woman-11: nahi ki thi

Interviewer: lakin apka hospital main karwane ka irada tha?

Woman-11: jee lakin jaldi hogya tha bacha

Interviewer: jese ap ne kaha k mujhey nahi zaroorat nahi pari hospital janey ki to wo kon se wajuhat hoti hain jin main apko hospital jana zaroori lagta hai?

Woman-11: ultrasound ki waja se ke kuch pata chaley ke bacha kesa hai sab thyek hai khoon test ho pata chale ke khoon ziada hai kam hai

Interviewer: acha ap ke ghar main faisley kon karta hai?

Woman-11: meri saas
Interviewer: ap center bhi un ke sath jati thin ya kisi or ke sath?

Woman-11: main akele jati thi center

Interviewer: apki saas ke kya khayal hain ke bacha hospital main ho ya ghar main?

Woman-11: hospital mai

Interviewer: jab sab ka khayal ak hi tha to hospital main call na karney ki waja?

Woman-11: ase hi nahi kya hogaya bas

Interviewer: idher health worker aati thin?

Woman-11: bas wo aa kar kuch sawal karti thi or chali jati thin

Interviewer: wo ap ko bolti thin ke apko center aana chaiye?

Woman-11: jee bolti thin

Interviewer: ap center ke ilawa or kahin gaen thin ilaaj ke liye?

Woman-11: nahi

Interviewer: TBA ne ap ke donu bachey paida kye hain un se apka taaluq kab se hai

Woman-11: jee unhon ne hi kye hain yahin hamarey paros main hain to unhain pata chala to wo aagaen thin

Interviewer: un ke sath delivery main apko ya acha lagta hai?

Woman-11: bas ziada takleef nahi hoti batata rheti hain sab

Interviewer: wo kuch laati hain apne sath delivery ke liye?

Woman-11: han ak injection lagati hain shayad dard barhane ke liye

Interviewer: or wo apko sab samjha rahi hoti hain?

Woman-11: jee sab samjahti hain

Interviewer: or ap ne kabhi pese kharch kye hain ilaaj ya delivery ke liye center main ya kahin or ?

Woman-11: nahi

Interviewer: dawai wagera main kabhi nahi?

Woman-11e : jee nahi

Interviewer: TBA ko kitney paise dene parey thy?

Woman-11: 3 hazar

Interviewer: or kabhi paison ka masla aya hai kuach hamal ke doran?

Woman-11: nahi

Interviewer: or center main fasley ka bhi masla nahi hota koi mushkil hoti thi aane jane main?

Woman-11: han door hail akin gari bulwati thi to wo aajati thi

Interviewer: or kuch asa k center main ap chati ho ke change laya jay?

Woman-11: nahi asa to kuch nahi

Interviewer: ap ke liye center ya hospital main sab se ahem cheez kya hai?

Woman-11: mere liye yehi hai bas ke ilaaj sahi se hi dawai miley or bas

Interviewer: pheli dafa main apka irada nahi tha hospital jane ka lakin dusri dafa main apko hospital jana tha asa kyu?

Woman-11: pheli dafa main mene socha nahi tha hospital ka lakin mujhey ghar main bohat ziada atkleef huwi is liye is bar mene socha ke hospital sahi hai lakin is baar ase jaldi jaldi main huwa ke kuch pata hi nahi chala

Interviewer: ab bachon ko center le kar aati hain ap teekey wagera lagwaney?

Woman-11: teekey to yahin aajate hain laganey

Interviewer: agar agli dafa apko bacha ho to kahan karna chaheingi?

Woman-11: irada to hospital ka hi hai center hi aongi

Interviewer: Or koi cheez hai jo ap batana chaen ya hum pooch na sakein ho?

Woman-11: nahi

Interviewer: koi bhi aesi cheez jo hum tabdeeli la sakein hamri sholiyaat main center main?

Woman-11: bas yehi ke dantna wagera na ho to sab thek hai us waqt wese itni takleef hoti hai to acha nahi lagta or bas

Interviewer: okay jee bohat shukriya.

___________________________X_____________________________

IDI WOMAN-12

Interviewer: ap ki umar kitni hai

Woman-12: 30 saal

Interviewer: 30 saal thek hai aur ap kitne arsey se shadi shuda hain

Woman-12: mere 13 saal hogaye hain shadi ko

Interviewer: mashallah aur ap k kitey bache hain

Woman-12: meri 4 beti 2 bete hain

Interviewer: thek hai aur ap ke saare bache hispatal mein paida huye hain ya ghar pe

Woman-12: ghar pe

Interviewer: thek hai aur ap ne kitni taaleem haasil ki hai

Woman-12: taleem yeh k quran shareef parha hai school parha hai

Interviewer: thek hai abhi ap kaam kar rahi hain ya ghar pe

Woman-12: nahi ghar pe

Interviewer: to hap mujhe bata sakti hain ap k khayal mein bache ko pata karne ki jagha sab se behtareen jagha konsi hai ap k khayal mein

Woman-12: sab se behtareen jagha toh hospital mein meri yeh joh abi huyi hai 8 maheena horaha hai munni ko toh kehti hun heart ki mareez hun BP ki mareez hunt oh rehri goth k center walon ne jawab diya that oh JPMC walon ka card banwaaya that oh 3 4 din mein JPMC mein admit thi toh phir in logon ne kaha ap ka case nahi leinge JPMC mein jao toh phir mein JPMC mein chali gayi toh un logon ne mujhe operation ka date diya jis din operation ka date diya dusre din meri munni 5 bajhe subha paida huyi

Interviewer: acha hai toh irada tha

Woman-12: han mera poora irada tha

Interviewer: thek hai toh lekin ap keh rahi hain ap k pehle bache bhi ghar pe hi paida huye

Woman-12: jee ghar pe paida huye

Interviewer: toh ap ko kia lagta hai k ap ko ghar pe kia ziada acha lagta hai

Woman-12: hospital mein sab se ziada yehi hai k hospital mein koi nahi tha bs meri ammi thi sirf toh ghar pe doctorni hamare baraabar mein hain isi k paas paida karaya tha

Interviewer: thek hai aur ap k khayal mein ghar pe paida karne k faidey kia hote hain

Woman-12: faida toh kuch nahi hai ghar mein paida kiye hain mein ne lekin phir bhi hospital mein gaye hain jahan par card banwaaya tha wahin pe usi subha dusre din gaye check up k liye

Interviewer: thek hai thek hai toh us k hawale se jaise kuch log hum jaise baat kar rahe hote hain woh keh rahe hote hain k ghar pe araam ziada mehsoos hota hai thek hai un k saath ghar waale hote hain

Woman-12: pata nahi hospital mein abi tak mein ne bacha paida kiya nahi joh pata chale ghar mein mujhe toh ghar mein sakoon milega sambhaalna uthna beithna sab meri ammi karti thi isliye

Interviewer: thek hai jaise ap keh rahi thin Aunty

Woman-12: han Aunty hamare ghar k barabar mein

Interviewer: toh in k saath ap ka tajaruba kaisa raha

Woman-12: achi Aunty hai na badtameezi na kuch koi koi hospital mein deakhein hain ab toh mere operation ka time tha phir bhi mein ne kaha mein nahi karongi phir meri ammi ne kaha nahi karwa lo lekin un k mareezon ko deakha toh mujhe bohat dar laga

Interviewer: kis cheez se dar laga

Woman-12: doctor ase daraate hain kehte hain k kia karo kia karo aesi baat thi kyun k bacha kabhi hospital mein paida hi nahi kiya isliye

Interviewer: thek hai woh sahi se baat nahi karte

Woman-12: nahi karte itna cheek cheek k baat karte

Interviewer: sahi toh ap jaise keh rahi thin k doctor ghar pe aati hain

Woman-12: jee ghar pe bhi aati hain un k ghar pe bhi jaate hain

Interviewer: thek hai

Woman-12: yeh barabar mein gali mein hamari samdan bhi hai na kyun ke mere cousin ka saas hai

Interviewer: oh acha sahi sahi toh jaan pehchaan bhi hai thek hai toh jab woh aati thin toh jaise woh koi cheezein le k aati thin

Woman-12: woh saamaan apna apna woh injection bhi lagaati hai goliyaan rakhti hain yeh sab saamaan is k saath saath hai

Interviewer: toh aur woh samjhaati thi k acha mein agar injection lagaaon gi toh kia hoga

Woman-12: han han k is tarhan ka dard hai kia karon mein sab bataati hain

Interviewer: acha aur ap k khayal mein ghar mein

Woman-12: mere khayal mein ghar mein kyun k mein ne hispatal mein bacha paida kiya hi nahi hai

Interviewer: toh lekin ap ko kia lagta hai k ghar pe paida karne se nuksaan kuch hosakta hai

Woman-12: abhi tak toh hua nahi kyun k mein ne 6 bache mashallah paida kiye hain Allah ka laakh laakh shukar na koi aesa nuksaan na koi aesi musibat Allah ka shukar hai abhi tak aaye nahi

Interviewer: aur saari deliveriyan normal huyi

Woman-12: normal huyi jee

Interviewer: koi ap k sehat k masaail

Woman-12: nahi nahi kuch bhi nahi Allah ka shukar hai kuch bhi nahi

Interviewer: aur jaise ghar pe koi ap k jaise bare hain ap ki koi behan

Woman-12: sab se bari behan meri ghar mein

Interviewer: toh ap k darmiyaan jaise ap log baatein karte honge toh bachon ki pedaaish k hawaale se koi khayalaat hain unhon ne bhi ghar pe kiya tha

Woman-12: han unhon ne bhi ghar pe kiya tha meri choti behan hai us k bache operation mein thy KGH mein paida huye abhi meri bhabi hai is ka bhi beta hua hai KGH mein paida hua hai

Interviewer: toh jaise ap log ek dusre se baat kar rahe hote ho ap ka tajaruba un k tajarube mein

Woman-12: hum sab ek hi baat hai na hum jaise maa behnein hain beti maa nahi hain maa behnain hain

Interviewer: sahi jaise k phir ap ko kia lagta hai k kin surate haal mein hisptal jana zaroori par sakta hai ya laazmi hojaata hai

Woman-12: jaise hamal k doraan mein ne center pe card bhi banwaaya har waqt check up k liye bulaate thy agar kabhi pait mein dard hota hai ya koi aur masla hota hai phir bhi hum jaate hain phir bhi hum jaate thy center mein check up karwaane k liye dawaai wagera mashallah center pe bhi acha intezaamiyaan hai acha check up hota hai kyun kk yahan par kiya hai

Interviewer: aur unhon ne ap se baat ki thi k hamal k doraan kia kia karna chaiye

Woman-12: han haamla doctor thi jaise har cheez k bare mein bataati thi k kis tarhan khana peena rakhna hai bachon k sath kis tarhan rehna hai yeh sab

Interviewer: aur jaise k delivery k time pe ap k khayal mein kin surate haal mein ya halaat mein ap ko hispatal jana zaroori par sakta hai us k bare mein ap se baat ki thi unhon ne

Woman-12: woh logon har waqt yeh bolte thy [woman 12] jab bhi ap bacha paida karte ho ghar pe is baar ghar pe paida nahi karo mein yeh kehti thi nahi ghar pe paida nahi karongi lekin kiya toh ghar pe paida kiya

Interviewer: ap ka pehle se iraada tha k ghar pe karna hai

Woman-12: kyun k hispatal se dar lagta hai

Interviewer: dar lagta hai

Woman-12: kyun k mein ne hispatal mein deakha hai KGH ka toh nahi deakha kyun k JPMC hispatal mein 3 4 din wahan pe admit thi mein ne daekha hai usi se us halaat se yeh log doctor mareezon k sath kis tarhan pesh aate hain mein ne deakha hai lekin mashallah meri choti behan ne abhi bacha paida kiya hai woh 2 maheeno ka hua hai mashalaah bohat ache tareeke se tha

Interviewer: woh bhi hispatal gayin thi

Woman-12: han hispatal mein hi hua tha

Interviewer: KGH mein

Woman-12: han

Interviewer: un ka tajaruba sahi raha

Woman-12: han

Interviewer: sahi sahi aur kisi se ap ne suna hai kabhi k un ka tajaruba acha nahi raha hai

Woman-12: KGH walon k center walon k buraai kabhi suna nahi kyun k meri bari beti is k baad ek meri beti 9 saal ki hai us k baad abhi 8 maheena meri beti ko horaha hai mein apna card banaati hun lekin bacha ghar mein paida karti hun

Interviewer: sahi acha to hap center kitni dafa arahin thi hamal k doraan

Woman-12: jaise mera chatta maheena (6 months) shuro hua hai mein aayi hun end tak

Interviewer: sahi hai to hap har maheene aati thin

Woman-12: har maheena ya maheene mein do martaba agar tabiat kharab hoti thi maheene mein 3 martaba bhi hota tha is tarhan

Interviewer: thek hai

Woman-12: mashallah goli dawaai, khoon ki drip sab kuch lagaate thy

Interviewer: hm hm hm toh bas mein wohi samjhna chah rahi hun k jaise ap keh rahin thi k ap ne shuru se kaha k ghar pe hi karna hai ap phir bhi arahin thi center pe ne toh center pe ap ko kia acha lagta hai

Woman-12: center mein jaise jaati thi agar meri tabiat kharab hoti thi mujhe injection lagte thy dawaai goli deity thin is se mujhe sakoon mil jaata tha

Interviewer: thek hai aur jaise ap keh rahi thin woh bolte toh thy [woman 12] ghar pe nahi karna

Woman-12: han [woman 12] bacha paida mat karna mein kehti thi nahi par phir bhi ghar pebacha paida karti thi

Interviewer: toh hum wohi janna chah rahe hain ghar pe

Woman-12: ghar pe meri yehi hoti thi na yeh raat ko mera bhai sirf hota hai mera husband joh hai maheeno mein 2 maheeno mein aata hai joh mera bara bhai hai woh bhi army mein hai woh bhi ghar pe nahi hota hai aur 2 chote chote hain bhaabi toh abhi aayi hain abhi ek saal horahe hain mein bache bhi chorna nahi chahti thi koi aur hai nahi isliye is wajha se pehle Aunty hamare saath thi isliye asaani hoti thi bache paida karne mein

Interviewer: thek hai aur waise ap ko kia lagta hai kis pe bharosa ziada karti hain hamal k doraan delivery k doraan jaise ap kehti hain ghar pe asaan toh ghar pe koi log hain jis pea p ziada bharosa karti hain

Woman-12: ghar pe hai na sirf hamari ammi hain aur koi hai nahi bhaabi hai abhi ek saal huye hain ghar pe aaye huye aur meri choti behan hai

Interviewer: aur sehat k hawale se ap kis par ziada bharosa rakhti hain jaise hispatal k doctor pe jaise ap keh rahi thi ap k paros pe hain

Woman-12: is k baad sirf usi time jaati thi jab delivery hoti thi sirf jis time time pe jaati thi

Interviewer: center pe

Woman-12: center pe jaati thi kyun k us k paas wohi end k time deity thi waise nahi

Interviewer: hm toh ap ko jaise ap center toh ap kehti thin bohat arahi thi aur ap har bache k sath arahi hain ap ne kab se shuru kiya hai center aana

Woman-12: 6 saal se arahe hain

Interviewer: 6 saal se arahi hain mashallah toh acha ap keh rahi hain ap har dafa center aati hain elaaj udhar karwaati hain acha lagta hai ap ko

Woman-12: acha hai kyunk izzat bohat karte hain

Interviewer: izzat karte hain thek aur lekin phir bhi ap ko haspatal se dar bhi lagta hai kabhi center walon se baat ki hai

Woman-12: mein jab bhi Dr. A se baat karti thi, who kehti thi [woman 12] nahi karne se galti huyi nahi karongi lekin karti thi apne dil ki

Interviewer: jaisa ap se kehti thi ghabraahat hoti hai

Woman-12: U baaji kehti [woman 12] ap har waqat yeh kahogi yahin pe paida karogi lekin ap ghar mein hi paida karo ek martaba ek bacha toh hamare paas paida karo is baar karogi mein ne kaha nahi phir bhi ghar pe

Interviewer: un logon ne ap ko bataya tha

Woman-12: un logon ne mujhe likh k diya na toh 3 din mein JPMC mein ek din cardio mein is tarhan yeh 3 4 din hospital mein thi dil ki dharkan bohat ziada hoti thi BP control nahi hota tha jab yeh mera munni choti thi woh dusra 9 saal ki beti hai saal do saal ki bachi thi mujhe 2 martaba chota heart attack hua hai

Interviewer: Allah!! Acha

Woman-12: han hua hai do martaba mujhe kaha k JPMC mein jao isliye

Interviewer: aur bhi bara masla hai

Woman-12: masla hai na isliye

Interviewer: sahi

Woman-12: ye log ne bola hamare paas aesi machine nahi hai Allah na kare ap ki tabiat kharab hojaaye kuch hojaaye toh phir hum log kahan pe le jaayein toh isliye kaha JPMC mein

Interviewer: toh jaise k delivery k time ap ki sehat pe koi masla hua

Woman-12: nahi abhi toh mein ne bola mein band karwaaongi lekin JPMC walon ne bhi operation nahi kiya hai isi blodd pressure ki wajha se abhi nahi hota mujhse bacha paida karna abhi kamzor hogayi hun bohat nahi hota taaqat nahi hai abhi

Interviewer: hm hm

Woman-12: waise Allah ka shukar hai 4 betiyan hain isliye mein ne kaha band karwaongi toh yahan par center walon pe le k gaya leikin wohi itna bara mera peit bhi nahi hai isliye operation kiya hi nahi keh raha hai ap ka peit bara hai sun nahi hota hai

Interviewer: nahi toh jaise waqfe k liye na

Woman-12: han mein toh bola bilkul operation kar k bas khatam

Interviewer: hm hm

Woman-12: lekin us ne bola ap ka operation nahi hoga ap ka peit sun nahi hoga yeh woh toh phir mein ne bola aaj toh yahan se aese nahi niklungi kuch na kuch kar lungi toh phir us ne 12 saal ka challa charwaaya

Interviewer: sahi thek

Woman-12: jaise ek maheena deid maheena takleef huyi thi jaise mera blood wagera menses aate thy phir jaate thy abhi mashallah sahi hai

Interviewer: sahi hai aur jaise ap center aati thin toh us mein ap ko koi mushqilaat ka saamna karna toh nahi para tha

Woman-12: kyun mushqilaat darwaaze se gaari le k jaati thi wahan se darwaaze pe a k chorti

Interviewer: acha aur ap akele jaati thin ya koi ap k saath aata tha

Woman-12: meri ammi hoti thi

Interviewer: thek hai sahi hai aur kabhi ap ne jaise hamal k doraan delivery k liye paise kharch kiye hain sehat ki sahuliyaat k liye

Woman-12: jaise ziada toh nahi is baar kuch ziada hi hua hai kyun k mere haalaat aese thy tabiat ziada kharab huyi thi isliye JPMC waale toh aese gande hain ek Panadol bhi nahi deite

Interviewer: Oh ho!!

Woman-12: nahi deite keh rahe hain bahar se le k aao nahi deite ek raat mere ko wahin pe check kiya keh rahe hain bibi ap ko bukhaar hai Panadol kha lo phir mein ne nurse se bola doctor ne bola Panadol la k bahar se le lo is se acha center tha center walon k bhi haath mein nahi hai toh is liye jaise teen din mein mere 10, 15 hazaar gaye hain

Interviewer: Oh ho!! Dr. Aunty ne kitne liye

Woman-12: Dr. Aunty joh hain jaise agar mein usay 3000 bhi dun ya 4 bhi dun waise toh us ka rate hai 6, 7 leikin hum se is tarhan nahi

Interviewer: kyun k ache talukaat

Woman-12: ache talukaat hain kyun k rishte mein hum samdun bhi hai aur mashallah gali mein sab isi se karwaate hain hamare paros mein abhi is baar joh meri munni huyi hai woh Aunty se nahi ki woh saamne TBA hai TBA se karwaayi thi bangaali hai

Interviewer: acha toh yeh joh ap ne saare bache Dr. Aunty k saath hi kiye hain

Woman-12: yeh Aunty ki hai us se ek choti Aunty ki hai phir yeh Aunty ki hai us k baad mein ne gaaon mein kiya yahan par nahi thi gaaon mein shift huyi thi apne susraal k paas yeh bhi bangaali tha abhi joh hai woh bhi bangaali hai

Interviewer: acha sahi

Woman-12: do bangaali hai

Interviewer: ap k TBA aur Dr. Aunty k mawaazna kar sakti hain k kaisa laga dono k sath

Woman-12: kuch bhi mujhe mehsoos nahi hua

Interviewer: mehsoos nahi hua koi fark

Woman-12: fark nahi mehsoos hua

Interviewer: sahi toh ap ko behtar kahan par laga

Woman-12: dono samjho mere liye maa thi kyun k meri ammi thi meri ammi aesi hai BP ka mareez hai ammi ko deakh k BP ziada bar jaati hai kyun k koi aur nahi hota tha na ek Aunty hoti thi yay eh Aunty bangaali hoti thi mashallah dono sahi kuch masla nahi hua

Interviewer: hm dono k sath acha laga delivery sahi hai

Woman-12: ab joh meri baby huyi khud pata bhi nahi chala k baby kaisa hua

Interviewer: bas hogaya

Woman-12: bas hogaya

Interviewer: toh jaise ap keh rahi thin ap parents k sath JPMC mein do teen din reh k wapis aagayin

Woman-12: han admit huyi thi mein

Interviewer: bola kia abhi time hai

Woman-12: time toh jaise mein phir gayi thi jab saatwaa maheena (7 months) tha mera jab mein admit thi hospital mein

Interviewer: thek hai

Woman-12: toh us k baad phir U baaji ne Dr. A ne mujhe un dono ne bataya k hum log ap ka case nahi kareinge ap JPMC mein jao kyun k ap ki condition is tarhan hai hum case nahi kar sakte phir mein JPMC mein gayi wahan jumme wale din ko JPMC mein gayi thi raat ko ek bajhe meri tabiat kharab huyi aur ghar mein ek aadmi bhi nahi tha toh phir mein ne ammi ko bola ja k jab tak Aunty ko bulao check kar lein phit Aunty ne kaha koi operation ka naubat nahi aaye ga mein paida kar lungi

Interviewer: hmm

Woman-12: toh phir ek bajhe se le k 5 bajhe tak , 5 bajhe mashallah mera baby hogaya

Interviewer: thek hai acha toh jaise ap ka hispatal ka tajaruba kab se shoru hua phir

Woman-12: hispatal mein yeh joh mera beta tha 6 saal ka hispatal mein toh jaati bhi nahi thi jab se yeh joh mera pehla beti hai joh meri tabiat kharab hogayi pehle toh mujhe pata nahi tha meri pehla bachi thi toh phir meri ammi mujhe civil hispatal le kar gaye thy tab hum log yahan pe naye aaye thy civil hispatal le kar gaye thy woh log bhi is tarhan se operation kareinge phir mera jheit ne mere ko utha k ghar pe le kar aaya phir Aunty k paas paida huye

Interviewer: konsa operation karaane waale thy

Woman-12: bara operation kareinge raat k 8 bajhe tak bacha nahi hoga toh hum log operation kareinge

Interviewer: acha cesarean

Woman-12: han toh mere jheit ne manah kiya tha mera jheit tha mera husband mera saas tha pehle bache k process mein mere saath that oh phir mere jheit ne manah kiya tha

Interviewer: hm hm hm

Woman-12: waise mere jheit ne ghar pe le k aaya raat ko hum log 10 bajhe aaye subha 7 bajhe paida hua Aunty k paas

Interviewer: jaise ap k jheit ne manah kiya tha koi wajha thi

Woman-12: keh rahe hain jaan booj k operation karwaayeinge

Interviewer: unhon ne ap ko wajha di thi

Woman-12: kuch nahi

Interviewer: nahi bataya tha

Woman-12: nahi kuch nahi bataya tha sirf yeh bataya tha k magrib k waqt pe azaan horaha that oh phir un log ne yeh bataya tha 8 bajhe tak baby nahi hua toh hum log operation kareinge toh phir mere jheit ne manah kiya

Interviewer: acha sahi hai jab ap ne Dr. Aunty k han bacha paida kiya tha toh phir woh bilkul normal tha

Woman-12: normal toh bilkul sahi

Interviewer: seedha nikla tha

Woman-12: abhi toh bohat kamzor hai mashallah hatta katta bachi thi meri

Interviewer: acha toh woh bas aese hi karwana chah rahe thy

Woman-12: han shukar mera jheit mere saath tha warna meri saas meri ammi meri phuppo saas yeh sab thy yeh toh mera operation karwa leite lekin mere jheit ne bacha liya

Interviewer: hm hm h map ko kia lagta hai ap k ghar mein jaise ap ki ammi kis jagha pe ziada bharosa rakhti hain hispatal ya ghar par

Woman-12: meri ammi toh manah karti hai ghar pe meri is condition ki wajha se meri ammi darti hai phir bhi mein ammi ko kehti hun nahi mujhe hispatal nahi jana woh mera bhai hai is ka husband woh

kehte hain hispatal cheez mein hispatal chalo lekin mein nahi gayi thi mein ne kaha mein ghar mein sahi hun hispatal mein bohat tangi hoti hai mujhe acha nahi lagta hai hispatal

Interviewer: ap ka joh dar hai hispatal se kab se

Woman-12: woh toh shuru se dar hai

Interviewer: waise ap ne kisi se suna tha

Woman-12: nahi waise bhi mujhe hispatal se dar lagta hai bacha ho ya bina bacha dar mujhe hispatal se bohat lagta hai

Interviewer: sahi hai toh hispatal k bare mein phir hum kia behtar kar sakte hain ta k jin ko bhi ghabrahat ho hispatal se dar ho toh hum un k liye behtar kar sakein ta k woh aayein hispatal

Woman-12: jaise ap logon ko acha lage mujhe na hispatal jana na bacha paida karna hai

Interviewer: jee jee jee sahi lekin jaise matlab ap k khayal mein agar kuch hota hispatal mein

Woman-12: hispatal mein jaise mujhe toh mein ne kisi aur ko deakha nahi hai mashallah meri choti behan hai us k teen baby hai

Interviewer: hmm

Woman-12: ek toh normal hau hai sab se bari aur do operation mein hua hai yahin par KGH mein hua hai us ko na kuch hua hai kuch pareshani na kuch problem kuch bhi nahi hua hai lekin bas mere dil mein kis liye kis baat ka dar rakha hua hai isliye

Interviewer: to hap ko lagta hai k agar doctor ap se sahi se baat karein

Woman-12: agar koi mujhe teizi se baat kare sab se pehle mere ko khud bardaash nahi hota hai lekin saamne waale se badtameezi na karo is baat pe

Interviewer: sahi aur unhon ne jaise ap ko pata hai k nuqsaanaat kia hosakte hain agar ghar pe paida karte hain toh us k sath

Woman-12: nahi mere saath toh kabhi hua nahi joh mujhe pata chale kia hota hai ghar pe kia nahi hota hai abhi tak toh kuch hua nahi hai

Interviewer: kisi se suna hai koi nuqsaan

Woman-12: center pe bhi kisi se kuch suna nahi hai aur ghar mein mashallah mere cousnein bhi hain joh bhi hai mera cousin ka ek saal horaha hai ek saal se bhi kam hai us ka bhi baba hua hai mera poora khandaan hispatal mein jaata hai mujhe dar hai

Interviewer: ap ko dar hai sahi thek hai sahi sahi

Woman-12: meri chachi hai abhi us ko do ek baba aur baby hua hai woh bhi hospital mein hua hai operation hua hai sab jaate hain hospital lekin mujhe nahi jana

Interviewer: acha center mein unhon ne ap ko jaise kuch aurton ka bacha ulta hojaata hai

Woman-12: ulta toh mera is ka bhi bataya tha doctoron ne keh rahe hain teira hai

Interviewer: kahan pe bataya tha center pe bataya tha

Woman-12: center pe bhi bataya tha aur kuch din mera woh joh teen talwaar nahi hai

Interviewer: hm hm hm

Woman-12: defence pe us din us ka aakhari ultrasound tha usi din yeh paida hua ghar pe

Interviewer: acha toh teen talwaar kahan se agaya teen talwaar pe ap ultrasound kara rahin thi

Woman-12: jee ultrasound karwaane in logon ne rangeen ultrasound k liye na joh is k bare mein bhi JPMC mein gayi hun

Interviewer: jee

Woman-12: toh un logon ne bhi likh k diya tha mere ko k wahan teen talwaar pe ja k ultrasound kar lo

Interviewer: acha

Woman-12: toh jis din mein ne bola k subha mein jaongi us mein pata nahi kis wajha se mein nahi gayi thi mein ne kaha kal jaongi 8 bajhe yeh paida hua ghar pe

Interviewer: hm hm hm hm lekin ultrasound toh hamare center pe bhi hosakta tha

Woman-12: nahi JPMC walon ne likh k diya tha

Interviewer: acha toh is doraan ap center pe bhi arahin thi

Woman-12: JPMC pe bhi gayi thi chek up k liye jis din center ka check up tha center pe gayi thi JPMC wale din ka JPMC pe gayi thi wahan rangeen ultrasound toh nahi hota na center pe

Interviewer: hm hm

Woman-12: toh JPMC walon ne rangeen ultrasound likh k diya

Interviewer: acha thek hai sahi sahi

Woman-12: toh is liye in logon ne bola teen talwaar mein mein ne bola sahi hai 3000 wahan se bach gaye toh mein ne Aunty ko de diya

Interviewer: hm hm hm sahi aur kisi se kabhi suna nahi k ghar pe paida karne se koi masla hua hai

Woman-12: nahi kyun k hamare ghar meri bari behan hai mashallah us k bhi 4 bete hain ek beti us k bhi sab ghar pe paida hua sab sehatmand hain mashallah ek toh bara beta yahin hai meri ammi k paas hai shuru se meri ammi k paas hai yahin par hai aur 4 bache mashallah un k apne paas hai

Interviewer: thek thek thek aur jaise ap k khayal mein aur koi rukaawatein jis ki wajha se aurtein hispatal jana na pasand karti hon

Woman-12: mujhe toh pasand nahi hai baaki logon ka mujhe nahi pata k kis ko kia pasand kia lekin mujhe pasand nahi hai

Interviewer: hm kyun k kuch like aurtein kehti hain ek toh jaise ap ne bhi bola woh badtameezi se baat karte hain

Woman-12: han badtameezi mujhe achi nahi lagti hospital walon ki is wajha se bhi mujhe acha nahi lagta

Interviewer: hm jaise jab TBA aati hai ghar pe ap ki ammi sath

Woman-12: ammi toh mere saath hoti hain lekin hospital wale nahi chorte

Supporter: main gate se nahi chorte

Interviewer: yeh JPMC ki ap baat kar rahe ho

Woman-12: KGH pe bhi nahi chorte

Interviewer: KGH pe nahi chorte thek hai

Woman-12: kyun k meri bhaabi ka baba hua hai na meri ammi andar thi meri nand bhi hai meri bhabi bhi hai keh rahe thy do bande nahi chorte lekin mein kehti hun mere saath meri ammi ho meri saas ho joh bhi ho sar k paas hi ho

Interviewer: hm hm hm

Woman-12: is wajha se

Interviewer: aur woh nahi aane de rahe

Woman-12: nahi aane deite woh toh nahi aane deite

Interviewer: aur kuch log yeh bhi kehte hain paison k masle ki wajha se woh aese hispatal le jaate hain ap ko kabhi paise ka masla aese

Woman-12: waise paise k mein ne bache ka check up k liye center pe gayi thin a mera paisa kharch hua center mein na kuch kiraaya wagera JPMC pet oh mein ghanjee hoti thi ek din bhi chali jaati thi check up k liye jaise 1000 1500 2000 mera araam se chala jaata tha kyun k kiraaya wagera yeh sab kuch hota tha

Interviewer: hm woh bhi faasle mein hoga ap kaise jaati thin riksha mein

Woman-12: nahi bus mein jaati thi

Interviewer: bus mein jaati thi

Woman-12: riksha mein wohi aana jaana hua toh wohi 4000 3000 ap ko pata hai kiraaya kitna mehanga hogaya hai

Interviewer: bilkul toh gaari k liye paise deine nahi parte thy

Woman-12: jab center pe jaati thi kabhi kabhi center pe gaari kam hote thy agar nahi hota tha agar time pe nahi pohche toh usi lalabaad tak chali jaati thi phir lalabaad se riksha kar k 150 200 toh center tak chali jaati thi kabhi kabhi waise toh ghar k darwaaze par chor deite thy le k jaate thy isi tarhan

Interviewer: thek thek

Woman-12: center se koi problem nahi hai

Interviewer: hm hm hm

Woman-12: JPMC mein toh mein kehti hun na ek Panadol bhi apne paas se nahi deite

Interviewer: acha toh center pe sab se behtareen cheezein hain ap ko kia pasand hai sab se acha

Woman-12: center mein mashallah sab kuch achi hai jaise masiyaan bhi hain center pe apne balochein hain tameez se baat karte hain agar doctorein bhi hain U baaji hon ya Dr. A ya koi aur ho sab ache hain mashallah

Interviewer: thek thek thek

Woman-12: kyun k mein jab yeh thi is k baad abhi choti huyi mashallah meri bhaabi ki abhi huyi mashallah 9 maheeney tak paida bhi KGH mein karna tha abhi bhi aaya aaj bhi aaya check up karne k liye aur meri bisma ka poochne k liye bhi har waqt aate hain

Interviewer: hm hm hm sahi

Woman-12: kyun k check up k liye abhi bhi aate hain agar abhi center walon k han taraf se is k liye bhi poochne aate hain k tabiat kaisi hai toh center se koi pareshani nahi hai

Interviewer: sahi sahi in ko bhi center pe le k jaati hain wahan elaaj hota hai

Woman-12: is ko mein do martaba le k gayi is ko bukhaar tha ek martaba le kar gayi thi

Interviewer: teekey weekey lage

Woman-12: han hifazati teekey aur jaise motion laga tha do martaba toh bas le k gayi thi

Interviewer: sahi

Woman-12: center se koi pareshani nahi huyi

Interviewer: sahi hai bas hispatal se wohi ghabraahat

Woman-12: han bas hispatal se hai par bacha hispatal se paida nahi kiya

Interviewer: shuru mein shayad mein ne poocha nahi lekin ap ko matlab koi nuqsaan ya koi nazar aata hai ghar pe bacha paida karne mein k koi masle ho sakte hain ap k khayal mein

Woman-12: mein ap se yehi keh rahi hun na mere sath koi aese masle nahi huye

Interviewer: thek hai sahi aur koi aesi cheez hai joh ap batana chahein shayad ap se poocha na ho

Woman-12: nahi ap ne pooch liya hum ne bata liya

Interviewer: chalein bohat shukriya

____________________________X______________________________

IDI WOMAN-13

Interviewer: apki umer kitni hai?

Woman-13: 26 saal

Interviewer: shadi ko kitna arsa hogya?

Woman-13: 8 saal

Interviewer: ap ke kitney bachey hain?

Woman-13: 3

Interviewer: sarey bache ap ke hospital main huwe hain ya ghar?

Woman-13: pehla wala ghar par phir dusra hospital meinor ab ye teesra wala bhi ghar mein huwa hai

Interviewer: ap ne taleem kitni hasil ki hai?

Woman-13: Quran Paak parha hai bas

Interviewer: or ap kuch karti hain kam ya ghar meinhoti hain?

Woman-13: ghar par hoti hun

Interviewer: ye bataen bacha paida karney ke liye ap ke nazdeek bhetreen jagha kya hai?

Woman-13: sab se bhetreen jagha agar normal ho to ghar par or normal na ho to hospital mein aksar doctors keh dete hain na ke apko normal hoga ya nahi to us hisaab se Jahan jana ho

Interviewer: toh wo kya samjhatey hain apko normal mein kya hota hai or jo normal nahi hote un mein kya hota hai

Woman-13: normal mein dard nahi hota hai operation mein dard aate hainor dard se bacha paida hota hai to agar dard aajay to ghar mainhi ho jata hai

Interviewer: ap hospital ya ghar kis ko tarjeeh deti hain?

Woman-13: ghar par

Interviewer: waja?

Woman-13: ghar par islye ke ghar par ho jane se ye hota hai aadmi na ho koi or bhi muhalley mein ho to ghar par karna parta hai is dafa meine ghar par kiya kyun ke mere shohar bhi nahi thy ghar par samundar gay huwe the is waja se mujhey ghar mein karna para

Interviewer: lakin apka apna irada kyat ha?

Woman-13: mera irada to hospital jana hota hail akin ghar par koi tha nahi mobile bhi shohar ke pas tha islye ghar par karna para wana hospital mein theek rheta hai wahan dard hote hainto asani hojati hai jism bhi acha rheta hai ghar mein bohat zor dena parta hai

Interviewer: ghar mein bacha karney ke kya nuqsanaat ho saktey hain?

Woman-13: filhal to mujhey kuch bhi nuqsan nahi huwa normal hi tha sab

Interviewer: or kabhi kisi se kuch suna ho agar?

Woman-13: wo to suna hai aksar ke khoon ziada aajata hai ya theli barh jati hai kabhi to uski waja se dard ziada hota hai

Interviewer: ap ne jab ghar par kya bacha to ap ke pas kon tha apki madad kis ne ki?

Woman-13: meri jethani hai na unko bulaya tha wo TBA ko laen thin unke jurwa bachey bhi TBA se huwe the to wo laen thin

Interviewer: or apka tajruba kesa raha TBA ke sath sab theek se kya unhon ne?

Woman-13: jee achar aha sab theek se kya tha

Interviewer: or wo TBA thin jis se phela bacha huwa tha?

Woman-13: nahi wo dusri thi

Interviewer: acha TBA mein apko kya kya cheezen samjhati hain un ke ilaaj main?

Woman-13: unka ilaaj to bas wo ak drip jis mai dard ka injection wagera hota hai wo laga deti hain bas

Interviewer: lakin jesa ap ne bataya hospital mein takleef kam hoti hai asa kyu?

Woman-13: wahan to drip laga dete hain na to dard kam hote hain ak hi baar mein jhatka hota hai wo

Interviewer: acha or TBA sirf injection de deti hai?

Woman-13: jee

Interviewer: TBA samjahti hai ke ye dya hai mene to is ke bad kya hoga kese hoga?

Woman-13: jee jee bata deti hai samjha deti hai

Interviewer: or hospital mein sab smjhaatey thy?

Woman-13: nahi khud hi khud kardiya bas

Interviewer: koi aesi cheez jo apko hospital ki sahi na lagi ho koi ilaaj ya doctor ap se theek se baat ka rahe thy?

Woman-13: nahi theek tha sab baat bhi theek se kar rahe thy

Interviewer: daant to nahi rahe the doctors?

Woman-13: nahi kyu dantenge hum to paise de rahe hain na, woh unhain danttey hain hain jo chillate hai nkyu chillate hain bardasht karlen khud thora

Interviewer: ap ke sare bachey normal the koi masla nahi huwa kabhi?

Woman-13: jee nahi Allah ka shukar hai normal huwe sab koi masey nahi huwe

Interviewer: acha mujhey puchna tha ke ap ke ghar ke jo barey hainwo kya chate hain hospital ko tarjeeh dete hain wo ya ghar ko?

Woman-13: wo to khete hain abhi jo dor chal raha hai na uski waja se khete hain hospital main hi karo

Interviewer: asa kis waja se kehte hain wo?

Woman-13: kyu abhi TBA jo hai na wo ase hi chor deti hai phele ki TBA zimedari se sab karti thi abhi to bacha hote hi kehti hain paisa do paisa do ghar jana hai or phir abhi ked or mein paani ki kami khoon ki kami ziada hoti hai pehle to asa kuch nahi hota tha islye kehte hain ke hospital mein hi paida karo

Interviewer: phir bhi apko ghar ziada behtar lagta hai ya hospital?

Woman-13: mein kya bataon apko mein keh rahi hun na mere shohar ghar par nahi the warna to mein chali jati hospital hi

Interviewer: acha koi nahi tha is waja se , warna to apko hospital hi behtar lag raha hai

Woman-13: jee han

Interviewer: kya ap bata sakti hain kon kon se ase nuqsanat hote hain jiski waja se humein hospital jana zaroori hota hai?

Woman-13: khoon ki kami bacha hote hi na maa ko ak jhatka sa lagta hai sarey jism mein ak dard hota hai jism Thanda par jata hai jiski waja s moat bhi ho sakti hai isi waja se hospital mein hi bacha karna behtar hai

Interviewer: hamal ke doran ap hamare center aati thin or kitni dafa jati thin?

Woman-13: han jab jab bulate the tab tab jati thi

Interviewer: apko center kesa lagta tha unka ilaaj wagera?

Woman-13: unka ilaaj to wo bas check kartey the ghar bhej dete the check kar ke laal goli or peeli goli dete the or bas ye hi dya tha mujhey

Interviewer: apko center ki sab se achi cheez kya lagti hai?

Woman-13: jo hamein maloomaat milti hain na wo sab se achi lagti hai ke bacha theek hai kesa hai ye sab

Interviewer: wo apko batate the ke kab hospital jana parega apko kab bacha hoga?

Woman-13: han tareekh dete the aur file, main ussi tareekh se chali jati thi

Interviewer: or kuch samjhatey the wo jese khoon ziada aa jana ya bachey ka ulta ho jana?

Woman-13: nahi wo bas samjhatey the ke paani ki kami ho sakti hai khoon ki kami ho sakti hai or kharish ho to aajana khoon ka masla ho to yahan aa jana sab samjhatey the lakin Allah ka shukar mujhey to asa kuch nahi huwa

Interviewer: or jab apka time qareeb tha delivery ka wo sab samjhaya tha? ke kese apko call karni hai kese jana hai?

Woman-13: han han sab samjhatey the humey wo Mahjabeen or Sanam hain na wo aati thin hamare pas sab samjhati thin

Interviewer: or ap ke tamam sawalaat ka wo jawab dete the? or kis tarhan bat karti thin wo?

Woman-13: jee sab jawab deti thin or ache se baat karti thin

Interviewer: center ya hospital ki koi cheez jo hum behtar kar saktey ho?

Woman-13: wo bachey ko nazla khaansi mein hum jatey hain na to wo khete hain bas ke bhaap do wo acha nahi lagta humein dawaai bhi nahi dete hum khana bager pakay bchey ke ilaaj ke liye jate hain dusrey bachey ghar chor kar unhain chaiye humein dawaai de kehte hain nazla hi hai bhaap lene se sahi hojaega lakin sahi nahi hota seena kharab hota hai un ke liye bukhaar hona zaroori hai to dawai dete hain wo warna khete hain k kuch nahi hai or hum jhoot bol rahe hain asa khete to nahi hain wo log lakin dawai nahi dete iska matlab hum samajh jatey hainke unhain jhoote lag rahe hain hum bas ye hi shikayat hai mujhey islye mujhey center jana nahi hota tha mere shohar mujhey khete hain ke tum jao file hai sab mein ajti hun to ulta mere sar main dard ho jata hai jao ao khana pakao aa kar phir dawai bhi nahi milti islye mein apne shohar ko kheti hun ke mujhey jana hi nahi hai, abhi bhi mere bachey ko seena jaam nazla hai lakin bukhar nahi hai is liye wo dawai nahi denge to jane ka kya faida? Main yehi bethi sahi hun mera pura dil tha jane ka lakin isko bukhar nahi hai jab ke seena kharab hai ankhon se pani bhi aa raha hai humey pata hota hai bacha kitna bemaar hai lakin wo log khte hain tum doctor ho maa ko pata hota hai na bachey ki halat to bas ye hi shikayat hai mujhey or koi nahi

Interiewer : ghar mein sarey faisley kon karta hai?

Woman-13: mere shohar or mein donu mil kar kartey hain

Interviewer: to ap donu ki raay ak hi hoti hai ke hospital mein karna hai ya ghar par?

Woman-13: jee ak hi khayal hote haindonu chate hain ke hospital mein ho

Interviewer: hospital ke ilawa ap kis par bharosa karti hain delivery ke liye?

Woman-13: Allah par Allah se bhetar kon waseela wohi bhejega na hamari salamati ke liye

Interviewer: jaise kuch log khete hain hum dai par bharosa kartey hainya kisi or clinic wagera par?

Woman-13: nahi nahi Allah par chor dete hainAllah khud asani karta hai hamare liye normal ho jata hai baaqi thori takleef to hoti hai warna bacha kese hoga to wo she leti hun mai

Interviewer: acha ye bataen center jane ke lye jo faasla hai us mein koi masla hota hai ap ja sakti hainasani se?

Woman-13: haan main tou paidal jati thi road tak jati thi paidal phir bus main beth gai koi masla nahi hota tha hum to bachey hone ke time chaltey hi rhete hainke chaltey jao chaltey jao jab tak bacha paida na ho

Interviewer: akele jati hainap ya kisi ke sath?

Woman-13: han baaqi meri ammi bhi gaen thi mere shohar bhi or meri jethani bhi gaen thin sath mere

Interviewer: kabhi pese dene parey hain alag se ilaaj k?

Woman-13: han unhain hum ne 5 hazar dya huwa tha clinic main dusrey clinic mein Jahan ye pehla bacha huwa tha center par to mein bharosa hi nahi karti thi tab

Interviewer: to apki soch kese badli phir

Woman-13: hamari parosan hai na Amna us ne kaha ke wahan jana chaiye Allah na karey kabhi bara case hojaaye koi masla hojay to wahan lelenge to hum file nahi banatey na kuch log to unhain kehte hain ke file nahi bana huwa to wapis ajao ghar to unka masla ho jata hai na to mene socha ke file banalun kisi time par agar zaroorat par jay

Interviewer: ap keh rahi thin ke agar normal ho bacha to ap chaheigi ghar par hojay to is ke bawajud ap keh rahi hain hospital ka

Woman-13: is ke time par to mujhey kuch bhi nahi tha normal type tha sab sab kam kye us ke bad hospital gay ak dam sab normal tha phir bhi gai thi mein hospital

Interviewer: kis hospital gaen thin phele bachey mein ap

Woman-13: surraiya wo hospital nahi hai ak chota sa clinic hai pehle to 5 hazar liye thy is ke time par ab to kher daur ke hisab se mehangaai hogai na 7 8 hazar lete hain

Interviewer: or dusrey wale ko jab ghar mein TBA ne kya to kitney dye?

Woman-13: 3 hazar

Interviewer: or koi rukawaten jinki waja se ghar mein bacha paida karna parta hai apko

Woman-13: nahi bas phone nahi tha to confuse hogai thi mein to jethani daaai ko le aai abhi to doctor ki tarhan karti hain TBA sab hota hai un ke pass pehle ki TBA to rassi se bandhti thi ab to clip laga deti hen

Interviewer: agli dafa ap ko bacha paida karna ho to kahan karna chaheingi?

Woman-13: hospital mein

Interviewer: apko ki date di thi ke kab hoga bacha?

Woman-13: han lakin mujhey 4 din bad huwa tha upper hogaye thy din

Interviewer: to ap ne koi intezam nahi kya huwa tha?

Woman-13: pese rakh diye the bas or kya intezam kartey logon ko to bithaney se rahe

Interviewer: nahi agar ap ke days upper hogay the tw koi mobile wagera ap sath rakh letin ke hospital walon ko call kar sakti thin na gari bulwa lein ya sab

Interviewer: us waqt yad nahi raha bas har waqt to ghar hote the bas us waqt yaad nahi raha

Interviewer: acha acha koi baat nahi or koi asi baat jo ap puchna chahein ya batana chahein jo hum pooch na sakey ho?

Woman-13: nahi bas

Interviewer: okay jee bohat shukriya

IDI WOMAN-14

Interviewer: apki umar kitni hai?

Woman-14: taqreeban 18 saal

Interviewer: apki shadi ko kitna arsa hogaya hai?

Woman-14: 1 saal hogaya hai

Interviewer: ap ke bachey kitney hen?

Woman-14: acha ek tha na jiska inteqal hogata ab ye dusra peit mai hai peit se hun mai

Interviewer: pehla ap ne ghar par kya tha?

Woman-14: nahi bangali paarey mai hai na wahan kyat ha

Interviewer: ap ne kitni Taaleem hasil ki hai?

Woman-14: 5 class

Interviewer: ap kuch kam karti hen?

Woman-14: nahi ghar par hi hoti hun

Interviewer: acha sab se phele ap mujhey ye bataen ke bachey piada karney ki sab se behtreen jagah ap ki nazar mai konsi hai

Woman-14: phela bacha Jahan mene kya us ke ilawa mai kahin nahi gai to mujhey to pata nahi hai dusri jaghan ka lakin wo jaghan mujhey theek lagi

Interviewer: ap kuch bata sakti hen ke wo jaghan apko kiyu achi lgi?

Woman-14: islye ke us ne phele thori parhai bhi ki or kam asani se kardya or jaghan bhi ghar jesi hi thi islye wo achi lagi or at karney ki bhi achi hen to acha lagta hai dil bhelta hai

Interviewer: ye bataen ke ghar mai paida karney ke kya faidey ho saktey hen?

Woman-14: faidey ye k hum khud ilaaj kartey hen na ye jo barey barey hen ye ilaj apni tareeqey se kartey hen to acha hota hai

Interviewer: acha or ghar par karney ke koi nuqsanat?

Woman-14: nahi asani hoti hai bahar to hum karney se dartey hen na ke hum asa karen to ye humey kuch bolen na ghar mai asa nahi hota asani se kam hojata hai

Interviewer: kissi waja se hospital gaen hen phele?

Woman-14: nahi mai center mai gai thi naam dakhil karwaya tha to bohat manzil mai chal nahi sakti islye is baar mene naam nahi karwaya dakhil. Gari bhi khete hen ap eik ho na ek ke liye nahi chal sakti gaari

Interviewer: hospital ke barey mai ap ke khayal kya hen ke wo kesi jaghan hai

Woman-14: hospital to mai kabhi gai nahi hun jo mujhey pata ho islye main nahi bol sakti asi hai ya wesi hai kyu ke mene kabhi wahan to nahi kya hai

Interviewer: kisi se suna ho kisi or se hospital ke barey mai?

Woman-14: han meri devraani hai na wo shyd KGH gai thi ye mje nahi pata lakin wo kehti hai ke wo bohat achi jagah hai mujhey pata bhi nahi chala asani se hogaya lakin main to nai gai na islye mujhey nahi pata

Interviewer: acha ap ke ghar ke baron ke bacha paida karney ki jaghan se mutaliq kya khayal hain ke kahan akrna chaiye?

Woman-14: mere susar barey hen wo bolta hai ke Jahan apko acha laga wahan karo mera kam to hai apko pese dena or achi salah dena baaqi Jahan bhi apko acha laga wahan karo mai jahan chaongi wahan jaongi waqt par

Interviewer: ap bata sakti hen kin masael ki bina par hospital jana zaroori hota hai delivery ke time par?

Woman-14: bohat manzil hai takleef hoti thin a to mai thak jati thi islye is baar mene naam nahi likhwaya

Interviewer: hamal ke doran ap kitni bar center gaeen thi?

Woman-14: pehla bacha to mera pait mai hi mar gaya tha phir mai wahan gai to unhon ne kaha ap pehle kyu nahi aen mene kaha manzil bohat hai islye main nahi aai upper se ap gari bhi nahi dte unhon ne kaha ye to ap ki galti hai mene kaha han galti to meri hai bas mujh se chala nahi jata itna mai thak jati hun to unhon ne kaha ke ap ka bacha to mar gaya pait mai ab ap ghar walon se ijazat le kar ao to hum apko KGH bhejen mene kaha theek hai mai ijazat le kar aati hun yahan aai to sab parehsan hogay or mandap le gay wahan 10,000 diye to mera kam asan hogaya

Interviewer: kon se month mai apka bacha mar gaya tha?

Woman-14: pure 9 mahine the

Interviewer: to hamal ke doran apko sehat ke masley the koi?

Woman-14: ulti to mujhey nahi aati hai, pait mai or kamr mai dard bohat hota hai wo to khatam hi nahi hota pheli baar bhi tha is baar bhi hai

Interviewer: kisi ne ap ko kuch samjhaya center walon ne ya kisi ne bhi ke kis waja se bacha mar gaya?

Woman-14: unhon ne mujhey kaha 3 din hogay marey huwe ap ko phele aana tha na taa ke hum apka checkup kartey

Interviewer: mandak jo ap keh rh hen Jahan se bacha karwaya tha to wahan phele bhi gaen thi ap?

Woman-14: nahi pheli dafa jab pata chala ke bacha mar gaya to kuch samajh nahi aya pareshani mai to wahan gay mujhey sab ne kaha meri dewrani wagera gaen thi wahan to kaha acha hai phir mai gai to mujhey bhi acha laga

Interviewer: ye wohi dewarani hen jinhon ne hospital mai karwaya tha?

Woman-14: jee wohi hen

Interviewer: to unhon ne apko kabhi bataya ke wo kon si jaghan ko ziada tarjeeh deti hen?

Woman-14: wo hospital hi kheti hen abhi mai naam likhwaya hai center mai, wo bhi pet se hai na

Interviewer: ap center kitni dfa aati thin?

Woman-14: jo tareekh wo dete the us par 9 mahine bas akhir mai der se gai 2 3 din to unhon ne kaha ap late kyu aai ho

Interviewer: or wo apka ilaaj kese karwa rahe the kya kya cheezen or dawai dete the apko?

Woman-14: bas dawai dete the drip bhi nahi lagatey the

Interviewer: center ka tajruba kesar aha apka kya cheez sab se bhetra lagi apko?

Woman-14: center mai to sab theek hai bas manzil door hai na islye main ahi jarh hun warna sab acha hai wahan

Interviewer: is ke ilawa sab theek hai wahan ap se bat wagera theek se kartey hen wahan?

Woman-14: jee sab sawalka jawab bhi dete the bat bhi theek se kartey the

Interviewer: or wo apko batatey the ke apko sehat ke hawaley se kya karna chaiye ya hamal ke doran kya mushkilat aa sakti jinki waja se apko hospital jana par sakta hai ye sab?

Woman-14: jee batatey the

Interviewer: kya bataya tha

Woman-14: wo khete the ap apna khayal rakho fruits khao or sb to mai sab khati thi goliya jo di wo bhi leti thi jo khete the wo karti thi mai

Interviewer: is tarhan ka kuch nahi bataya ya samjhaya ke khoon ziada aa jata hai kabhi ya bacha ulta paida hota hai?

Woman-14: nahi ye sab to nahi han test hote the mere ultrasound wagera mujhey dil ki dharkan sunate the mere bachey ki jab bachey ki dharkan band hogai to wo bhi bataya to mai to wahin rone lagi thi

Interviewer: unhon ne apko batay nahi ke ab kya karna hai?

Woman-14: nahi unhon ne mujhey kuch nahi bataya bas danta ke late kyu aaen mene kaha han meri galti hail akin manzil door hai islye gaari ki waja se der se aai mai

Interviewer: phir jab ap ghar aaen jab bachey ka pata chala to kis ne kaha ke TBA ke pas jana chaiye?

Woman-14: nand meri wo thori chalak hai to ase kam wo sanbhalti hai hum sab ke

Interviewer: pure hamal ke doran faisley kon kar raha tha?

Woman-14: hum sath sab susar nand wagera sab

Interviewer: nand ko kese pata ke TBA ke pas jana chaiye?

Woman-14: usey pata hai in sab ka uski apni beti ke do bachey hen islye usey pata hai

Woman-14: faisley lete waqt nand ka ziada hota hai ya ammi ka

Woman-14: jo sahi salah de uska a us ne kaha TBA achi hai sab ne bhi kaha ke han achi hai to wahan chalay gaye

Interviewer: or apki ammi ka kya khayal tha?

Woman-14: meri ammi ka tha ke jism ai meri bachi ke liye asani ho nand ne kaha TBA ka to wahan chale gay meri ammi bhi mere sath thi

Interviewer: center walon ne apko samjhaya tha ke gari aegi lene apko or sab?

Woman-14: jee samjhay tha number bhi dya tha

Interviewer: or ye nahi samjhaya ke kya kya nuqsanaat ho saktey hen hamal ke doran bacha ulta hojana yakhon ziada aajana?

Woman-14: jee ye to samjhay tha

Interviewer: ap kis par bharosa karti hen delivery ke liye ke mujhey in se bacha karna hai?

Woman-14: mujhey to Allah par bharosa hail lekin TBA bohat achi hen us par bharosa hai mai kl bhi gai thin a to us ne kaha ke ap ache khane khaon to mai khati hun us ne dawai bhi di hai mujhey to mujhey bharosa to Allah par hai or phir TBA par

Interviewer: acha jese ap keh rahi thin ke apko rastey ka masla hai door ka chal kar jati thin ap gari bhi nahi aati thi

Woman-14: jee wo khete the ap akeli ho or akeli ap ke liye gari nahi aa sakti to mai paidal jati thi or apko paidal pata hai ap to gari mai aai ho ap ko kese pata hoga

Interviewer: jee jee hamla aurat ke liye paidal aana bohat mushkil hai , or ap akele jati thi ya kisi ke sath?

Woman-14: jee meri ammi ke sath

Interviewer: to wo log kitney log chate the gari mai?

Woman-14: jese mai apne paarey mai akeli hamla thi islye to wo khete the 2 3 hamla ak sath ho

Interviewer: pese kitney kharch hote the hamal ke doran ap k?

Woman-14: center mai to nahi huwe baaqi TBA ke pas jo karwaya tha bacha to 10,000 diye the center walon ne to kaha tha ke operation hoga jab ke TBA ne asani se normal kardya to itna to haq banta hai uska

Interviewer: apko operation se dar lagta hai?

Woman-14: jee mujhey operation se dar lagta hai mene to kabhi kisi ka hote huwe bhi nahi dekha

Interviewer: TBA ne ilaaj kese kya apka?

Woman-14: dawai di thi abhi to mujhey itna yad nahi shayad injection lagaya tha goli wagera or bhi kuch dya tah lakin mujhey yad nahi

Interviewer: to apko koi dard wagera nahi huwa?

Woman-14: nahi dard mujhey bas hamal ke doran hota hai kamar mai or pet mai bad main ahi hota

Interviewer: unhon ne samjahya tha ke in sab goliyo wagera se kya hoga?

Woman-14: han samjhaya tha goli di thi ke apka bacha to mar gaya hai agar sansen hoti to wo khud bahar aajata lakin phir goli se kya

Interviewer: kabhi ap ne hospital ke barey mai kuch suna kejee wo to mene suna hai sab se ke hospital wahan ye hota hai wohota hai?

Woman-14: jee wo to mene suna hai aksar ke hospital mai sab acha hota hai aram se hojata hai pata bhi nahi chal jata lakin phir mai unki baton ko ziada gor nahi karti mujhey dar lagta hai hospital ki baton se

Interviewer: dar kyu ap to phele kabhi gai nahi thi logon se sunney ke bad bhi apko jyu dar lagta hai ksi se kuch bura suna hospital ke hawale se?

Woman-14: nahi bas bemaari se hospital se itna darn ahi lagta jitna wahan mareezon se lagta hai

Interviewer: lakin hamla aurton ke liye aka lag room hota hai wahan alag se

Woman-14: wo bat nahi hai yahan kartey hen sab baten ke hospital acha hai wahan sab ache se ho jata hai acha amla hota hai

Interviewer: hamal ke doran ap gahr par karna cha rahi thi ya hospital?

Woman-14: mai to ghar mai hi cha rahi thi ke kam hojay hamarey yahan to ziada tar ghar mai ho jata hai jab ziada mushkil aajay koi to hospital jatey hen warna gahr mai hi

Interviewer: center ka apko kese pata chala?

Woman-14: wahan ke amley se or meri dewraani wagera or bhi bohat aurten gaen thi tw wo btati thin ke bohat acha hota hai sab wahan to mai sab se puch kar gai sab ne kaha kam asani se hojaega koi takleef bhi huwi to wo sab ilaaj kar dete hain

Interviewer: phir bhi ap ghar par karna chati hen asa kyu?

Woman-14: wo to jesa time aega us hisaab se kabhi hospital kabhi ghar bas ghar mai hogaya to theek warna hospital

Interviewer: or koi rukawaten jinki waja se ap ghar mai karna chati

Woman-14: ab ye to bas abhi pet mai jo mere bacha hai iska bhi Jahan naseeb hoga bas Allah Paak asani se kardey sab baaaqi koi rukawat nahi mujhey acha lagega to mai hospital jaongi bas manzil door hai

Interviewer: ap apna agla bacha kahan karwana chati hain?

Woman-14: Jahan mujhey acha lagega mujhey koi rukawat nahi shohar bhi mera kehta Jahan jana chaho susar bhi bas manzil door hai baaaqi abhi socha nahi ghar mai sahi lagta hail akin koshish karungi ke hospital mai ho

Interviewer: gari ke ilawa or koi aesi cheez jis se apka khof nikal jay hospital ka ya hum kuch behtri la saken?

Woman-14: nahi bas hospital se nahi mareezon se khof aata hai aur bemariyan se, lakin ap keh rahi ho ke kamra alag hota hai mai to gai nahi kabhi wahan tak phir Allah na karey jana huwa to phir mai dekhungi

Interviewer: or koi aesi baat jo ap batana chahein ya hum ne poocha na ho?

Woman-14: jee nahi bas line wine bhi lagti hai to wo bhi masla nahi bas manzil bohat door hai

Interviewer: okay shukrya

___________________________X_____________________________

IDI WOMAN-15

Interviewer: sab se pehle mein ap se poochna chah rahi hun ap ki umar kitni hai?

Woman-15: meri 25 saal

Interviewer: 25 saal thek hai aur ap ki shadi ko kitna arsa hogaya hai?

Woman-15: 11 saal hojaayega

Interviewer: 11 saal hojaayega thek aur ap k kitne bache hain?

Woman-15: mere 8 bache

Interviewer: 8 bache thek hai aur ap k yeh joh saare bache hain kahan kahan pe paida huye

Woman-15: ghar pe

Interviewer: ghar pe huye sahi aur ap ne kitni taaleem haasil ki hai

Woman-15: taaleem yeh k quran parha hua hai

Interviewer: acha thek hai aur ap kahin pe kaamkarti hain ya ghar pe

Woman-15: nahi nahi ghar pe hoti hun

Interviewer: thek hai acha sab se pehle mein ap se poochna chah rahi hun k bachon ki paida karne ki sab se behtareen jagha konsi hoti hai aur kyun

Woman-15: mere khayal mein doctor k paas hoti hai

Interviewer: hm hm acha toh ap ko kyun lagta hai doctor k paas ziada behtar hota hai

Woman-15: waise toh mein kabhi gayi nahi hun abhi kamzori ki wajha se menses k sath yahan pe ghar pe nahi hosakta paani bhi nahi hai na jism k

I’ve never been to the hospital. I can’t do home births because of weakness, there is less water in my body

Interviewer: hm toh ghar pe ap ko kaisa lagta hai paida karna

Woman-15: yeh 2 3 ghante lag jaate hain

Interviewer: 2 3 ghante lag jaate hain aur ap ka tajaruba kaisa raha hai

Woman-15: sahi

Interviewer: sahi raha hai ap ko acha lagta hai ghar par paida karna bacha

Woman-15: han

Interviewer: aur TBA ko bulaate hain ap

Supporter: TBA nahi woh madam hota hai

Woman-15: woh madam hota hai woh bhi doctor hai woh drip lagaati hain

Supporter: aesa nahi hai 8000 9000 us ko bhi deina hota hai

Woman-15: woh 9000 10000 le leita hai

Interviewer: acha us ka naam kia hai

Woman-15: us ka toh afshan naam hai

Interviewer: acha toh woh hamesha joh aath (8) bache huye hain un sab k le rahi hain

Woman-15: nahi woh gaaon mein thi pehle 3 bache wahan paida kiye ghar pe hogaya mashallah TBA ki zaroorat nahi pari jaise hi bache paida hote hain in k abbu TBA ko le k aajaate hain aese hi hua mere saath

Interviewer: acha sahi toh ghar pe ap ka kis tarhan se woh TBA aati hai kia le k aati hai

Woman-15: gaari le k aati hain

Interviewer: gaari le k aati hain

Woman-15: dur hai woh dur rehte hain gaari le kar aate hain yahan

Interviewer: toh un ka woh kia karti hain dhood pilaati hain maalish karti hain kia karti hain

Woman-15: dhoodh nahi bas aese hi

Interviewer: acha aur sahi hai aur woh idhar wali joh TBA hain woh bas kuch nahi le k aati

Woman-15: kia

Interviewer: jab woh TBA aati hain bacha paida karwaane k liye toh woh apne saath kuch samaan le kar aati hain

Woman-15: nahi woh nahi laati kuch bhi khaali pan hum log rakh deite hain wahan ki baat kar rahi hain yahan toh bohat kuch chaiye hota hai wahan khaali teil laati thin bas

Interviewer: sahi aur yahan k liye

Woman-15: yahan Dettol pamper sab kuch teil , blade

Interviewer: sahi aur acha ap bata sakti hain ghar pe kia faide hote hain bacha paida karne se

Woman-15: koi faida nahi hai yehi nuksaan hai TBA bhi itna late aati is liye toh AKU se form bhara tha

Interviewer: hm hm

Woman-15: gareeb haalat hai bacha ziada hogaya jaan ki kamzori khoon ki kami hogaya toh woh log apni taraf se lagaate

Interviewer: hm sahi hai nuqsaanat kia hosakte hain gahr pe bacha paida karne se konse nuqsaanat hosakte hain ghar pe paida karne se

Woman-15: kuch bhi nuqsaan nahi hota dawaai mangwa leite hain bahar se woh likh k de deite thy

Interviewer: hm nahi jaise ap keh rahin thi k ghar pe karna ap ko sahi nahi lagta hai na

Woman-15: han

Interviewer: toh kyun kis wajha se

Woman-15: yehi k paani ki kami hojaata hai insan ko

Interviewer: hm

Woman-15: ghar par dard bhi to nahi aatey na bas is wajha se bol rahi thi

Interviewer: sahi acha ap ko koi masle huye hain delivery k time pe

Woman-15: nahi nahi koi masla nahi

Interviewer: sehat k masle ya bachon k koi pedaaish k time pe koi masle huye hon

Woman-15: yehi hota hai bacha mar jaata hai

Interviewer: hm

Woman-15: do abhi dono bache aesa hua hai

Interviewer: dono bache

Woman-15: han pehle beti tha woh bhi aesa hogaya

Interviewer: acha toh abhi acha do bache aese zaaya huye hain

Woman-15: han

Interviewer: acha sahi ap kehte ho 8 bache toh yeh 8 mein se do zaaya huye hain?

Woman-15: abhi 6 zinda hain baaki do mar chuke sab mila k 8 hogaya

Interviewer: acah thek hai thek hai ok samjh mein agaya aur ap bata sakti hain k joh bhi ap k ghar mein khaandaan mein joh bare hain log aur ammi abbu ya saas susar joh bhi un k kia khayalaat hain kahan pe karna chaiye bache ko

Woman-15: susar toh mar gaya faut ho chukka hai saas hai khaali woh toh bichaari to apni hi us mai kaha bhi nahi jata un se to

Interviewer: hm thek hai aur aesa kuch sunne mein toh koi baat k hispatal mein nahi jana chaiye ya hispatal k bure tajarubaat hon joh ap ne sunewe hon is ki wajha se dar lagta ho

Woman-15: nahi

Interviewer: hm aur acha mein yeh janna chah rahi hun k agar ap ko ghar pe paida karna sahi nahi lagta hai toh ap ne 8 bache ghar mein kyun paida kiye hain

Woman-15: woh toh wahan gaaon mein thi na 3 bache wahan kiye

Interviewer: acha 3 bache udhar huye thy

Woman-15: yeh bhi doctor k jaise hi hain drip wagera lagaate hain

Interviewer: gaaon mein?

Woman-15: nahi yahan pe yahan Karachi pe

Interviewer: thek hai

Woman-15: yeh drip laga leite thy goliyan bhi khila dete thy dard ke wahan paida kiye hain mujhe jahan acha laga wahan paida kiye

Interviewer: toh woh un k ghar pe jaati hain

Woman-15: ghar pe jaati hain wapis a bhi jaati hain woh

Interviewer: thek hai thek hai sahi hai toh is wajha se ap ne socha mujhe center aana shuru kar leina chaiye

Woman-15: mein ne yeh socha hai k us ne yeh baat mujhe bola kamzori bohat mehsoos horahe thy toh center pe bacha wahan paida kar leina wahan pe elaaj bhi sahi hota hai khoon ki zaroorat hua toh woh bhi laga leinge woh toh mein nahi laga sakti tujhe jahan acha lage wahan chale jana us ne jawab diya toh mein center chali gayi

Interviewer: acha toh yeh mashwara ap ko kis ne diya tha

Woman-15: yeh ammi ne diya tha

Interviewer: ammi ne sahi sahi thek hai phir ap ne joh kis bache se ap ne center jana shuru kiya

Woman-15: ek bacha hai abhi is se abhi joh faut hua

Interviewer: abhi joh faut hua us se

Woman-15: pehle abhi nahi

Interviewer: sahi hai acha center ap kitni dafa a jaati thin hamal k doraan

Woman-15: mein har maheene mein jaati thi time deity thi na 10 taareek 9 taareek 2 taareek mein chali jaati thi

Interviewer: aur ap tajaruba center k sath kaisa raha hai udhar se achi cheez ap ko kia pasand aayi ap ko

Woman-15: sab sahi hai wahan khoon test bhi pasand agaya har waqt test karte thy saans ki bimaari pata chal jata tha woh bhi aur ultrasound bhi karta tha sab kuch acha laga mujhe

Interviewer: sahi sahi jaise ap center ja rahin thi toh ap ka iraada tha hisptal mein bacha paida karein

Woman-15: nahi mujhe irada tha center pe jaane ki yehi tha matlab wahan chale jaongi wahan ache insan ko dekh leinge kahin aesa na ho khoon direct hojaaye woh log deakhte bhi hain ek insan ko bharosa nahi hota na phir yeh baat mein ne socha tha lekin nahi hua wahan chale gaye thy saalgirah pe wahan aese haalat hogaya tha

Interviewer: acha acha sahi jaise ap chah rahin thi k phir center pe through woh log hispatal bhijwaate hain na pedaaish k doraan toh ap chah rahin thi k phir aap center k through hispatal mein paida karein ya bacha ghar pe hi hojaaye ap kia chah rahin thi

Woman-15: mein !! mein toh center jana chah rahi thi

Interviewer: center pe

Woman-15: han wahan pe elaaj bhi kiya mein ne woh har din chocolate bhi de deite thy har din 10 din k baad 5 din k baad khoraak joh ap ko bataai chocolate shaasha phir mein khaati thi us k baad meri haalat sahi hua phir baad mein jab mein gayi na haalat kharab hogayi thi

Interviewer: hm acha ap mujhe bata sakti hain k center pe ap ko sahi se samjhaate thy sahi se baat karte thy

Woman-15: han

Interviewer: joh bhi ap k sawal thy woh us ka jawab deite thy

Woman-15: han

Interviewer: aur unhon ne ap ko yeh samjhaya tha k kia pedaaish k time pe nuqsanaat ho sakte hain ya koi bhi masaail ho sakte hain jis ki wajha se hispatal jana par jaaye ya bacha ulta hota hai

Woman-15: nahi nahi inhon ne bhi yehi bola tha koi bhi aesa masla hojaaye ap foran pohach jana yahan phone kar deina

Interviewer: unhon ne yeh bataya tha k kis tarhan k masle hosakte hain

Woman-15: han woh toh bolte hain koi bhi masla hojaaye sakina beithe mat rehna ghar pe yahan ajaana joh shaasha deite hain mujhe har waqt samjh k jaate hain bolte hain is takleef mein mat beithna koi bhi masla hojaaye hamein bata deina hum gaari mein pohcha deinge

Interviewer: thek hai acha aur center pea p ko yeh bhi samjhaate thy k jab ap k delivery ka time aayega ap k liye gaari aayegi ap ko is number pe call karna hoga paise itne rakhne hain matlab woh thora sa kharcha hota hai ward ka hispatal k liye lekin yeh sab bataya k ap ka time hoga phone kar deina

Woman-15: han yeh bataya tha

Interviewer: sahi aur ap ko samaan rakhna hai jab ap jaayeingi hispatal yeh sab bataya tha

Woman-15: han

Interviewer: thek thek aur sahi is k elaawa mein ap se poochna chah rahi hun k center ki koi aesi cheez thi joh ap ko achi nahi lagi ya behtar kar sakte hain un ko

Woman-15: nahi nahi sab ache thy

Interviewer: sab sahi tha ?

Woman-15: sab sahi tha mashallah

Interviewer: sahi acha aur ap ki ammi bhi yehi chahti hain k jahan bhi ap chahein hisptal mein karein in ka kia khayal hain

Woman-15:   in ke?

Interviewer: han

Woman-15:   in ke bhi yehi khayal hai

Interviewer: sahi hai toh ap dono k khayalaat ek hi hain thek hain aur center aur hispatal k elawa kis pe bharosa rakhte hain k bache ki delivery k liye

Woman-15:   woh attiya

Interviewer: joh TBA hain

Woman-15:   nahi

Interviewer: joh hospital mein

Woman-15:   attiya joh ap logon k AKU mein

Interviewer: acha han han thek hai

Woman-15:   wahan woh sahi hai

Interviewer: acha toh thek hai aur ap bata sakti hain k aesa koi masla hota hai faasle ka ya aese koi ap kis tarhan se jaati hain center pe

Woman-15:   mein toh gaari mangwaati hun

Interviewer: acha gaari mangwaati ho woh aate hain

Woman-15:   call kar leiti hun ajaate hain

Interviewer: sahi ap kis k sath jaati hain

Woman-15:   mein akeli jaati hun

Interviewer: sahi hai kabhi aesa hua hai k paise ap ko kabhi paise kharch karne pare elaaj k liye

Woman-15:   nahi nahi

Interviewer: ya dawai k liye

Woman-15:   nahi

Interviewer: sahi hai paison ka masla kabhi hota hai is hawale se

Woman-15:   nahi nahi

Interviewer: sahi hai aur thek hai acha to hap ne phie se center pe aana is liye shuru kiya kyun k ap k khud k sehat k masle horahe thy ap ne kaha behtar hoga mein center jaon

Woman-15:   han socha mein ne

Interviewer: sahi hai jab ap ka ye hap keh rahin thi k ap kisi birthday mein gayin thi saalgirah mein gayin thi us doraan phir aap ka bacha zaaya hogaya us k baad phir ap ne kia kiya

Woman-15: us k baad kuch bhi nahi

Interviewer: un ko pata nahi chala than a ap kahan gayin thi hispatal

Woman-15: nahi nahi ghar pe

Interviewer: center pe kab gayin thi ap

Woman-15: center mein kabhi nahi gayin yahan pe aayi thi na us ko file mein ne de diya kaha k masla ho gaya un logon ne bola sahi hai woh aate rehte hain poochte rehte hain in ko de diya file woh log poochne lage kia horaha tha mein ne kaha koi masla nahi mein ne bataya nahi un ko bole acha

Interviewer: acha toh ap kisi aur k ghar gayin huyi thi jis ki wajha se gaari nahi a paayi

Woman-15: han file nahi tha number tha asal baat yeh hai file mein ghar pe chor gayi thi mujhe toh nahi pata tha mujhe jana tha 9 taareek ko unhon ne time diya hua tha mujhe toh nahi pata achanak aesa hua

Interviewer: hm hm hm aur yeh aathwwe(8) maheeney mein tha ya 9 maheeney

Woman-15: 9 maheeney yani k 9 din 10 din

Interviewer: sahi hai toh jab phir ap ne in se baat ki unhon ne kuch kaha ap ko k kyun hua ya kuch bhi ap se baat ki is bare mein k kis wajha se zaaya hua bacha aur ap se aesi koi baat ki k ab joh zaaya hogaya is ka bhi kia elaaj kar sakte hain

Woman-15: nahi nahi mujhse nahi kaha

Interviewer: sahi hai toh phir unhon ne nahi kaha k operation karana hai kuch bhi

Woman-15: nahi nahi normal hota hai mera bacha

Interviewer: bache matlab ap k 9 maheeney hogaye thy toh is k liye operation karate hain nab ache nikaalne k liye

Woman-15: nahi mere toh normal hote hain ghar pe bhi normal hi hota hai

Interviewer: sahi hai aur unhon ne yeh kuch nahi kaha hai k kia masla tha ya ultrasound mein nahi deakha unhon ne

Woman-15: unhon ne bola sahi hai ultrasound jab bhi nikaala bacha normal hi bataya

Interviewer: sahi hai unhon ne yeh kuch kaha k kitne din huye thy ya kuch aur koi aesi rukaawatein pehle ap hispatal mein nahi jaati thin ghar ko tarjeeh deity thin

Woman-15: asal mein baat yeh thi wahan pe hispatal tha dur sahi hai wahan pe gaaon ka elaaqa hai in ke abbu zameen pe kaam karte thy toh paise nahi hote thy is wajha se ghar pe bacha hojaata tha majboori k halaat pe sab wahan aesa hai is wajha se ghar pe bacha hojaata abhi toh yahan Karachi pe aayi hun doctor k paas ja rahi hun phir inhon ne bola AKU sahi hai us se file bana dein har din ek jaisa nahi hota mein ne bola sahi hai mein file bana deity hun is wajha se mein center aayi thi

Interviewer: acha sahi hai thek hai

Woman-15: file bhi meri achi bani thi

Interviewer: achi bani thi

Woman-15: han mein is ko bura nahi keh sakti file mujhe achi bana k diya tha in logon ne phir mere naseeb ki baat thi

Interviewer: ap ka irada tha ap hamal mein thi toh ap ka irada tha k ap hispatal mein karein ya ghar pe karein

Woman-15: mein toh center chahongi

Interviewer: center chaheingi sahi hai

Woman-15: mein ne ammi logon ko bola tha

Interviewer: toh center wale jaise ap ko bacha paida karne ka time aata hai na toh gaari toh phir hispatal bhijwaate hain toh ap kia chah rahi hain k ghar pe hi hojaaye ya phir hispatal pe hi hojaaye

Woman-15: us time haalat aesa tha center pe karne wali thi ap ko bola tha number nahi tha phir mein phone nahi kar saki mein wahan se aayi raat k 10 bajh chuke thy raat ko kon aayegi haalat kharab mein gaari bhi nahi thy bike se aate toh file le k jaate koi nahi tha wahan pe drip laga diya ek ghanta do ghanta bacha hogaya mara hua tha

Interviewer: sahi hai toh jaise ap center aayin thi toh ap ko batatey honge k jaise k delivery ka time kareeb araha hai toh phir kia kia karna hai is tarhan ki koi baatein huyin thi number hai joh call karein jab time ajaaye yeh bataya tha

Woman-15: han bataya tha

Interviewer: bataya tha thek hai acha aur aese hisptal k bare mein ap k kia khayalaat hain

Woman-15: hispatal?

Interviewer: hispatal jaise koi ward wagera jaise woh kehte hain na agar ap ko delivery karni hai ap hispatal mein hi kareingi toh is k bare mein ap ka kia khayal hai ap kabhi gayin hain hispatal

Woman-15: bachon ko le jaati hun kabhi bukhaar ajaata hai

Interviewer: toh matlab koi dar war ya koi bura tajaruba aesa koi jaise jin se koi baat kar rahe hote toh bata rahe thy k hum kabhi gaye nahi hain hispatal un ko dar lagta hai unhon ne koi kahaniyan suni hongi kisi aur se na ap ko dar lagta hai

Woman-15: han

Interviewer: toh ap ko kis cheez se dar lagta hai

Woman-15: mujhe yeh lagta hai k yeh log aese na kar lein phir ammi log toh yeh bolte hain k woh hamare jaise insan nahi hain woh kia kar sakte hain

Interviewer: hm toh kia sahi nahi karein k jaise kis cheez se ap ko dar lagta hai

Woman-15: mam k woh log koi bolta hai k jaane se jab case ho jaata hai khoon dilate hojaata hai aese koi masla hojaaye is baat se mein darti hun

Interviewer: hm hm

Woman-15: aur koi baat nahi

Interviewer: hm lekin wahan koi aesa masla ho toh hispatal is liye hai agar koi masla ho toh usi waqt us ka elaaj

Woman-15: han woh toh hai woh toh ammi ne samjhaya tha us wajha se bol diya kyun k hum log gaaon pe rah k aayein hain na demag aqal nahi tha ab aaya hai

Interviewer: nahi koi baat nahi

Woman-15: han isi wajha se abhi joh mein soch rahi hun na center k bare mein joh hai na sahi hai center abhi koi masla hojaaye na mein wahin pe jaongi aese koi masla nahi hai center pe

Interviewer: aur ap kia bachon ko le k jarahi hain center un k teeke weeke elaaj k liye

Woman-15: han mein toh yahin se lagaongi center se

Interviewer: toh sahi acha agar phir se agar ap hamal se hongi

Woman-15: han

Interviewer: tabhi ap kia chaheingi ap center pe aayeingi

Woman-15: han mein aaongi

Interviewer: aur hispatal ka iada irada hai ya ghar pe

Woman-15: nahi mein wahan jaongi center

Interviewer: center pe sahi hai

Woman-15: yahin se file banwaaongi yahin se jaongi

Interviewer: aur koi cheezein ap ne suni hain hispatal k bare mein jaise kisi ki koi waaqiyaat sahi nahi hai ache tajarube thy kia suna ap ne hispatal k bare mein

Woman-15: bohat suna hai mein ne ke jab phone karte hain jab insan ki tabiat kharab hoti hai tab nahi aate gaari mangate hain foran nahi aate yeh shikaayat hum ne suna hai aur yeh baat hai mere bhai k yahan baby ap logon k AKU wahan pe hua tha normally sahi tha woh toh bolte hain bohat acha hai wahan bacha sahi paida hota hai ghar se acha hai wahan woh sambhaalte hain jab tak bache hojaate hain tab take k ghanta do ghanta un logon ko bithaate hain

Interviewer: sahi aur koi rukaawatein hain jin ki wajha se ap kahin hispatal ya center nahi ja saktin aur ghar pe deliver karna sahi hai

Woman-15: nahi kuch bhi nahi

Interviewer: koi rukaawat nahi

Woman-15: nahi nahi

Interviewer: acha aur kia cheez hai jaise center mein agar koi cheez mein behtari la paayein ap k khayal mein jaise center k bare mein kisi bhi cheez mein agar ap ko achi nahi lage us mein hum behtari la paayein jis ki wajha se aur aurtein aana chahein

Woman-15: nahi nahi woh toh hai mere devar aaye hain un ko mein ne bataya tha bohat ache se center mein file banaate hain un ko bata diya bola han mein chali jaongi baaki mere sath tu bhi chale jana file banaane k liye lekin udhar scissor hi hota hai na do bacha scissor mein hi hua tha bola k scissor hota hai us k liye file nahi banaate

Interviewer: un ka operation hua tha

Woman-15: han un k file nahi bante thy

Interviewer: nahi nahi file toh bante hain zaroor bante hain jab woh pedaaish ka time aata hai na woh doctor hispatal pe woh log deakhte hain

Woman-15: yeh aese k yeh log bol rahe meri bhabi wahan jaayein mein ne bola center pe sahi hai normal hoga toh normal hojaayega bacha, operation hoga toh operation ka har cheez unke paas maujood hai

Interviewer: hm hm hm

Woman-15: woh toh hai na mein gayi nahi hun mein ne suna hai mein gayi thi devar k sath

Interviewer: bas wohi deakhte hain joh haamla aurat hai us ka elaaj karte hain aur jab delivery ka time aayega toh woh gaari mein hispatal bhijwa deite hain center pe agar bilkul emergency hai phir bache paida hote hain agar time nahi hai ghar se nikalne wala toh phir center pe us ki delivary hoti hai warna yeh log wahin chahte hain hispatal mein sahulat ziada hoti hai toh wahan pe normal bhi hota hai agar un ko lag raha hota hai k masla horaha hai us ki wajha se operation bhi karna parta hai

Woman-15: mein ap ko bataon mein center se file kiyun banwaai thi. Pehle joh meri beti guzar gayi thi na us ki zaroorat thi oxygen nahi bolte

Interviewer: hm hm

Woman-15: us ki zaroorat thi mere paas paisa nahi thy mein gaaon se aayi thi

Interviewer: hm hm hm

Woman-15: yahan pe aayi hun 3 saal hogaye phir mein yahan apna ghar le k yahin beith gaya mein nahi jana chahti wahan pe kaam dhanda nahi hai ab nahi jana chahti mein jab center se file banwa rahi thi toh har cheez deakhte hain na woh log is wajha se mein ne bola ke dubara bacha ho to mere ase nahi zaya ho to is waja se file banwai thi wahan se kyu ke mere pas to pesa nahi tha oxygen lagane ke liye fridge mai rakhwane ke liye to mai kya karti phir is waja se mene file banwai thi ye ap log puch rahe hen to mai bata rahi hun

Interviewer: kya apko pata hai kea p ke bachey kyu zaya huwe ap ke khayal mai ?

Woman-15: mujhe to lagta hai meri hi galti hai kyu ke mai gai hun na wahan to raat mai gai hun to rastey mai wo jagha kharab thi wahan se guzar huwa tha is waja se meri bachi ka nuqsan huwa tha lakin center

mai jab gai tou izzat se baat bhi ki or acha raha hamesha sab mai kyu jhoot bolun mujhey dubara bhi jana hai wahan bas ak bat jo mene apko bola bachi ki dharkan ki waja se la parwahi ki thi bas mere 9 mahine mai huwa tha ye to mai dar gai thi lakin mai dubara gai to kaha sab sahi hai dharkan sahi hai wo kisi or aurat ka bata dya tha mujhey

Interviewer: to kitney din bad apko pata chala

Woman-15: 15 din ke bad

Interviewer: theek hai or koi asi cheez jo ap batana chaen ya hum ne puchi na ho?

Woman-15: jee nahi

Interviewer: jab apko phle kaha dharkan sahi nahi hai or dubara jane par kaha ke harkan sahi hai to unhon ne apko waja nahi batai thi ke phele kyu nazar nahi aai thi dharkan ?

Woman-15: nahi unhon ne kuch nahi bola tha mere pas par kursi par ak aurat bethi thi us ne mujhey kaha ke mere bachey ki dharkan nahi hai to mene kaha mujhey bhi ase hi kaha hai to us ne kaha nahi mere bachey ka kaha hoga wo log bhool jatey hen lakin mai dubara un ke pas nahi gai mai gari le kar foran ghar par aagai phir ammi ko batay to unhon ne mujhey kaha tum 2 3 din bad dubara jao phir mai gai dubara ke ab mujhey alag feel ho raha ap mera ultra sound dubara Karen to unhon ne phir mujhey kaha ke sab sahi hai ap pareshan nahi ho hum ne galti se apko bol dya tha hum ne kisi or ko bola hoga ap ne apna samajh liya

Interviewer: phir apko kab pata chala kitney din bad ke apka bacha expire hogaya ?

Woman-15: wahan se aane ke 15 din bad

Interviewer: theek hai shukria.

___________________________X_____________________________
