# Supplementary material for: Why do women still give birth at home; perceptions of Pakistani women and decision-makers from marginalized communities
Source: PLOS Glob Public Health. 2023 Oct 13;3(10):e0002217. doi: 10.1371/journal.pgph.0002217 (PMC10575520; doi:10.1371/journal.pgph.0002217)
Supplement: S2 Data — (PDF) [file pgph.0002217.s002.pdf]

IDI DM-01

Interviewer: apki umar kitni hai?

DM-01: 40 saal

Interviewer: ap ne kitni taleem haasil ki hai?

DM-01: taleem bilkul bhi hasil nahi ki hai bas Quran thora thora parha hai.

Interviewer: konsi jamaat tak parha hai?

DM-01: 2 main thi to nikal gayi thi khud dil nahi lag raha tha.

Interviewer: ap inki kya hain (Woman-01 ki)?

DM-01: main inki saas hun

Interviewer: ap koi kaam karti hain?

DM-01: machine chalati hun bijli wali.

Interviewer: apki raaye main ek aurat ko hamal k doraan kin saholiyaat ki zaroorat hoti?

DM-01: TBA ki ya phir maa ho to acha hota hai na ho to chal jata hai

Interviewer: in ke liye TBA ap thi?

DM-01: ji han

Interviewer: aur koi aesi cheez hamal k doran koi saholiyaat or koi khidmaat?

DM-01: nahi sahi rehta hai ghar mai.

Interviewer: khane peene ki koi saholiyaat ya kaam ka kam ziada hona?

DM-01: khane peene ki zaroorat bhi puri hojati hai agar kabhi kam par jaaye toh bhi kar leite hain kahin na kahin se.

Interviewer: aur in ke elawa dawaai wagera or dusri kya zarooratein hoti hain?

DM-01: dawaai wagera ki zaroorat to hoti hai doctor k paas jaatey hain woh likh kar deite hain toh store se le kar aate hain

Interviewer: ap k khayal main ek aurat ko delivery k doran kin khidmaat ki zaroorat hoti hai?

DM-01: delivery bhi hojati hai hamarey yahan joh hath ki dawaai wagera banai jaati hai usi se insan sahi hojata hai ya ziada koi takleef hojaaye toh doctor ko bula kar aate hain woh injection wagera lagata hai to sahi hojata hai.

Interviewer: acha doctor ko ap kahan se bulaati hain?

DM-01: yahan paas wala family doctor hai usi ko bula leite hain kyun k isi masley ka ilaaj woh karta hai.

Interviewer: acha wo kahan par bethtey hain, center par to nahi hote hai na?

DM-01: nahi nahi qareeb bethta hai wo hamare ghar se. agar ziada takleef hojati hai toh barey hospital chale jaate hain.

Interviewer: ap k khayal main ek aurat ko bachey ki pedaaish ghar par karwana sahi hai ya kisi health center ya hospital main?

DM-01: ghar par hota hai to ghar par karwa lete hain lekin agar hospital ka boldein toh jana chaiye hospital, mujhey ghar par ziada sahi lagta hai kyun k hum ne bhi ghar main paida kiye hain isliye hum boltey hain ghar main paida karen toh woh sahi hai sehat k liye bhi.

Interviewer: mujhe ye bataein kya bachey ki pedaaish k mutaabiq koi aise ache ya bure tajarubaat hain?

DM-01: ache hi hain burey to nahi hain

Interviewer: jaise in k do bachey huye un main koi aise tajarubaat joh burey hon?

DM-01: ache hi huye the bas insan ko kabhi paison ki kami hojaati hai to udhar lena parta hai phir bachey jaate hain darya par wahan se kama kar aate hain toh wapis de deity hain.

Interviewer: koi aisa bura tajaruba raha jaise in ki sehat k hawaley se hamal k doraan ya baad main koi masla?

DM-01: aisa hai k isko qabz hoti hai ziada is k ilawa to koi mushkil nahi, sehat k liye khana khichri doodh wagera bhi deite hain jis se sehat achi ho ye mere jitni moti thi lekin isko qabz nahi khatam horahi.

Interviewer: koi aisa masla jiski waja se bohat kamzor hogai ho?

DM-01: nahi aisa kuch nahi.

Interviewer: acha jaise k doctors hote ap ne bataya woh family doctor toh unhein bulane main un se raabta karney main koi masla hota hai apko?

DM-01: raabta nahi yahin betha howa hota hai toh jaa kar bula kar laatey hain.

Interviewer: aisa kabhi hua k hamare center se aise kisi ko bulana para ho apko?

DM-01: nahi nahi aisa kabhi nahi hua hai.

Interviewer: ap TBA ko bulatey hain?

DM-01: han woh aajaati hain phone karney se.

Interviewer: in k (Woman-01 ki) dono time par aai thi?

DM-01: ji waise inko main le kar gayi thi yahan nazdeek ghar hai phir ek din main beti k ghar thi ghar jab aayi toh yeh nahi thi maine kaha ye itna bara peit le kar akele kahan chali gayi, ye khud chali gayi thi daai k maine kisi se pocha toh pata chala phir main gayi daai k toh ye wahan thi main bethi kuch deir samaan wagera diya laa kar or 15-minute main hogaya bacha normal.

Interviewer: aap TBA ko bohat pehle se jaanti hain?

DM-01: han jantey hain ek TBA to bohat purani hain jis ne hamare bhi bachey kiye or iska bara wala bhi usi ne kiya, agar woh boldein k bacha ghar main nahi hoga to phir hum chale jatey hain hospital le kar.

Interviewer: aise kabhi hua k TBA ne kabhi galat mashwara diya ho apko ya koi aisa jisey ap janti ho jisey kabhi galat mashwara mila ho TBA se?

DM-01: nahi nahi kabhi nahi hamare saath to nahi hua.

Interviewer: aur joh woh delivery karti hain woh sab bachey zinda hi hote hain?

DM-01: han han zinda hote hain Allah ka shukar hai jiski zindagi hoti hai, or jinki nahi hoti wahan se zindagi usey hum tum ya hospital wale bhi nahi bacha sakta.

Interviewer: kya ap k khayal main bachey ki pedaaish se mutaaliq inki apni raaye aeham hai?

DM-01: han bilkul aeham hai joh faisla karein ghar main karna hai to theek ya hospital main jana hai to bhi theek hai woh tension nahi hai.

Interviewer: aur jaise ap kahein k ghar main karna hai or ye kahen k nahi hospital main toh kya le kar jaainge hospital?

DM-01: Ji toh le kar jaainge hospital kyun k marzi to karney wali ki hoti hai ab jisey dard ho wohi janta hai mujhe ya apko dard ho to hum janey na. jisko hota hai wohi janta hai.

Interviewer: apki raaye main kuch khawateen apne bache ki pedaaish k liye ghar ka intekhaab kyun karti hain?

DM-01: shayad usey sahoolat ho ghar main paida karney k lye door bhi jana na parey asani se ghar main hojaaye.

Interviewer: is k elawa koi baat raazdari wagera ki waja se karti ho?

DM-01: han ye bhi raazdari ki wajah se bhi

Interviewer: kabhi aisa hua center par ya hospital main aayi ho or bura tajaruba raha ho?

DM-01: nahi aisa kabhi nahi hua.

Interviewer: toh ap k nazdeek hai islye intekhaab karti hain ghar ka k wahan asani hojati hai aram se?

DM-01: han aram ziada hota hai or takleef bhi kam hojati hai TBA dawaai deti hai us se yaa hum khud bhi apne liye bana lete hain usi se insan sahi hojata hai or teesrey din uth jata hai, hospital main ye hota hai k 3 din tak wahan raho or phir us k baad ghar par aa kar bhi parey raho.

Interviewer: aur faasley ka ya door ka koi masla hota hai?

DM-01: nahi aisa kuch masla nahi hota.

Interviewer: kya apko lagta hai haamla aurton ki zindagi main koi rukawatein aisi hoti ho jinki wajah se woh chahti ho k ghar main hi karlo bacha?

DM-01: nahi nahi is time to ab yeh nahi chahti hai kahin na kahin se Allah ka shukar hai kaam hojata hai.

Interviewer: koi or aisi baat joh ap batana chahein ya joh hum ne na poochi ho?

DM-01: nahi nahi aisa kuch nahi hai……

______________________________X_____________________________

IDI DM-02

Interviewer: apki umar kitni hai?

DM-02: 63 saal

Interviewer: ap ne taleem hasil ki hai?

DM-02: Nahi

Interviewer: apka Woman-02 ke saath kya rishta hai?

DM-02: chachi hain

Interviewer: abhi ap kuch kam karti hain ya ghar hi hoti hain?

DM-02: Ghar hi hun abhi

Interviewer: ap k khayal main ek haamla aurat ko hamal k waqt kis tarhan ki sehat ki sahuliyaat chaiye hoti hain?

DM-02: acha khana wagera

Interviewer: bachey ki pidaaish k waqt haamla aurat ko kin cheezon ki ziada zaroorat parti hai ap ke khyal mai?

DM-02: taaqat wali cheezen chaiyen hoti hain jis se maa or bacha sehatmand ho doodh wagera

Interviewer: toh yeh cheezein kahan se milti hain jaise ap ne kaha k TBA ko bulaya tha ap ne to kya woh le kar aati hain ye cheezein

DM-02: woh toh sirf bacha paida karwati hain

Interviewer: toh in sab cheezon ka intezaam kon karta hai?

DM-02: ghar par pari hoti hain , jaise kheer hogai ya ache ache khane ki cheezein hogai

Interviewer: ap ki raaye main bacha paida karney k liye konsi jagah behtar hoti hai hospital ya ghar?

DM-02: ghar main

Interviewer: wajah bata sakti hain k ghar main kyun ya kya faidey hote hain?

DM-02: kabhi gai nahi main hispital lekin ghar main hi Allah hamari madad karta hai ghar main hi sahi rehta hai

Interviewer: kisi aise ko ap janti hain jinka hospital ya ghar main bacha paida karna acha nahi raha jiski wajah se ap ko lagta hai k ghar main karna behtar hai?

DM-02: agar hamein pedaaish se pehle pata chal jaaye k bacha taira hai ya bachey ya maa ko koi masla hai andar to hum kehte hain k ab jana chaiye humein hispital ab ghar par nahi karwana chaiye

Interviewer: jaisa ap ne kaha ap hispital nahi gayin kabhi to ap ko kaise pata chala k hispital jana sahi hai ya nahi

DM-02: muhalley main bohat se aise waqiye hote hain

Interviewer: koi misaal de sakti hain k kis tarhan ke masaail ho saktey hain hispital main ya koi aisa case suna ho ap ne apne as paas

DM-02: nahi

Interviewer: toh ap khud se bolti hain k ghar par karna chaiye?

DM-02: Jee han

Interviewer: koi mukhtalif wujoohat hain ap k paas hispital na janey ki ya kabhi kuch aisa suna ho ap ne jis se apko lagta hai k ghar main sehat k masaail hal karna zaida behtar hai?

DM-02: ghar main sahi hai bas hispital main pata nahi kya kya hota hai ilaaj bhi theek se nahi hota islye ghar main theek hai bas

Interviewer: apki raaye main apko kya lagta hai k aurtein kyun ghar main bacha paida karna chahti hain?

DM-02: operation se dar lagta hai

Interviewer: kyun dar lagta hai koi wajah?

DM-02: us se masla nahi hota lekin hispital main jaane se operation ki wajah se maa or bachey ko kuch ho sakta hai is wajah se.

Interviewer: kabhi suna ya dekha hai ap ne aisa kuch hote huye kisi k sath jiski wajah se ap aisa sochti hun?

DM-02: nahi dekha nahi suna bas apni raaye hai

Interviewer: apki raaye mani Woman-02 ki pasand zaroori hai agar yeh chahein k hispital main karwana hai ghar main nahi toh

DM-02: Jee agar kahegi toh hispital le jaayeinge

Interviewer: matlab agar agley bache main Woman-02 hispital jana chahein toh ap le aajengi?

DM-02: Jee le jaayeinge

Interviewer: acha hamal ya pedaaish se mutaaliq koi aesi cheez ya baat jo hum nahi pooch sakein ho lekin ap batana chahein?

DM-02: nahi koi nahi hai

Interviewer: center main koi aesi cheez ya baat aesi hai jiski wajah se apko lagta ho k aurtein chahti hain k woh bacha ghar par hi karlein center ya hispital pohanchney main mushkil hoti hai ya kya wajah hai ap k khayal main?

DM-02: sahi hai acha hai sab kuch center main

Interviewer: apko kaise pata ap toh kabhi gaen nahi?

DM-02: gayi hun 1, 2 baar

Interviewer: acha to apko lagta hai sab thek hai center main

DM-02: Jee gareebon k liye acha hai

Interviewer: aese kon se masaail hain apki nazar main jinki wajah se apko lagta hai ghar main bacha paida karna behtar hai? jaise center main aana jana ya faslay ki wajah se mushkil lagta hai ya apko lagta ho k wahan kharchey ziada hojaenge to aapki nazar main aisay koi masaail hain?

DM-02: masley toh hote hain pohanchney k hogaye jaise gaari nahi hai toh jaane ka masla islye bohat koshish kartey hain k ghar main hi hojaayein.

Interviewer: toh gaari ki wajah se matlab faasla ka masla hota hai?

DM-02: Jee han

Interviewer: phone wagera hote hain k araam se aap call karlein center ko gari k lye?

DM-02: han phone hote hain lekin gaariyan to dur hoti hain

Interviewer: phir ap TBA ko kis tarhan bula rahi hoti hain?

DM-02: TBA to aas paas hi rehti hai

Interviewer: toh kya ap TBA ko pehle se jaanti hain?

DM-02: han hamari parosan hi hai bohat pehle se janti hain

Interviewer: or ap ke apne bhi tamam bachey ghar main hi huye the?

DM-02: Jee han saat bache ghar main normal huye hain

Interviewer: acha kya is wajah se apko lagta hai k ghar main asaani se hojaatey hain bache?

DM-02: Jee han

Interviewer: hamare liye koi sawal ya kuch aesa joh ap batana chahein shayad hum ne poocha na ho?

DM-02: nahi

Interviewer: okay, shukriya bohat bohat

____________________________X___________________________

IDI DM-03

Interviewer: acha jee to hap ki kia umar hai

DM-03: hamari toh abhi shayad arsat(68) hai

Interviewer: acha arsat (68) saal hai acha jee toh ap ne kitni taaleem haasil ki hai

DM-03: hum khali primary 5 jamaat

Interviewer: 5 jamaat parha hai

DM-03: jee

Interviewer: sahi aur mujhe bataayein k ap ka unse kia rishta lagta hai jis se andar interview liya gaya hai

DM-03: han bahu hai

Interviewer: acha sahi aur ap ki maujooda mulaazmat ki kia hasiyat hai

DM-03: hamari dukaandaari hai parchiyon wala

Interviewer: sahi sahi sahi acha toh mujhe batao ap ki raaye mein ek aurat ko hamal k doraan kin sahulaat ki zaroorat hoti hai kia lagta hai ap ko

DM-03: woh toh sahi hai hispatal mein karta hai hamal toh woh sahi hai hispatal

Interviewer: hispatal mein nahi waise kia zaroorat hoti hai like ap ki raaye kia hai is bare mein k jab aurat hamal se hojaati hain shuro toh us ko kia zaroorat hoti hai kis cheez ki konsi khidmaat ki zaroorat hoti hai un 9 maheeno mein

DM-03: han khoon woon thi

Interviewer: khoon woon thy .. acha sahi us k elawa kuch test wagera ap ki nazar mein zaroori hote hain agar karwana

DM-03: jee karna parta hai

Interviewer: test karna parta hai sahi aur jab woh udhar aati hai na center pe toh udhar kia kia karwaati hain joh ap ko sahi lagta hai?

DM-03: woh to aap ne poocha hoga unse

Interviewer: jee

DM-03: hamari koi problem nahi hai hum udhar gaya nahi

Interviewer: jee

DM-03: un ki committee dusri hai

Interviewer: jee jee jee sahi sahi hogaya koi masla nahi hai acha ab ap k khayal mein jab aurat zachgi k doraan jab hoti hai jab bacha paida hone wala hota hai toh us waqt kia zaroorat hoti hai kis cheez ki ap ko lagta hai

DM-03: us din matlab k kabhi kabaar toh idhar hi hojaata hai han

Interviewer: hmm

DM-03: bacha paida hojaata hai ghar pe

Interviewer: hmm

DM-03: nahi toh phir hispatal ki zaroorat parti hai

Interviewer: sahi hai sahi toh us time pe jab bacha paida horaha hota hai toh un k us waqt kia zaroorat hoti hai

DM-03: us waqt?

Interviewer: hmm.. kis ko bulana parta hai ilaaj karwaana hota hai

DM-03: us waqt doctor ko dikhata hai

Interviewer: hmm hmm

DM-03: sahi

Interviewer: sahi

DM-03: aur TBA bhi hota hai

Interviewer: hmm

DM-03: gharelo

Interviewer: jee ghar pe jee jee

DM-03: gaaon ki TBA hoti hai

Interviewer: jee jee jee sahi sahi hogaya toh mujhe bataayein ap ki raaye mein aurat ko jab bcha ghar pe us ko matlab woh karna chaiye zachgi k doraan ghar pe hi karna chaiye ya hispatal jana chaiye ap ki nazar mein ap ko kia lagta hai

DM-03: nahi abhi joh x ray wagera horahe hain

Interviewer: han

DM-03: x ray hain

Interviewer: hmm

DM-03: bacha abhi ziada bra hojaata hai phir pedaaish hoti hai

Interviewer: sahi

DM-03: ghar pe nahi huyi toh hispatal mein thek

Interviewer: sahi hai waise agar normal chal raha ho saare toh ghar pe behtar lagta hai ap ko ya phir hispatal jana

DM-03: woh ghar pe behtar hai

Interviewer: kia wajha hai

DM-03: kharche warche bach jaate hain

Interviewer: kharche ka masla lagta hai ap ko

DM-03: han

Interviewer: sahi toh kharche kis cheez pe hota hai

DM-03: bhaara kiraya dawaai

Interviewer: dawaaiyan hmm lekin center pe toh sab muft hai

DM-03: center pe muft hai kabhi kabaar aesa din aata hai joh ap ka band bhi hota hai upar neeche ka

Interviewer: hmm hmm hmm

DM-03: shayad muharram ka chelam ka ya aesa din ajaata hai 12 rabi ul awal ka eid wagera ajaati hai is mein phir band sahi

Interviewer: hmm hmm

DM-03: baaki toh center ka kaam sahi chal raha hai

Interviewer: jee jee jee jee ap kabhi aaye hain center

DM-03: hum nahi aaya hai

Interviewer: acha ap bhi aaye aap ki deakhein

DM-03: han

Interviewer: shayad ap ko acha lage (laugh) kia pata .. acha mujhe bataayein k ap k us mein kabhi jitney bhi ap k saal guzre hain us mein ap ko aesa mehsoos hua hai bache ki pedaaish k waqt koi bura tajaruba hua ho ya koi acha tajaruba hua ho bache ki pedaaish k doraan ya hamla k doraan

DM-03: woh bas idhar k joh hai na TBA joh hai

Interviewer: hmm hmm

DM-03: TBA hai us ki wajha se hojaata hai kyun k us k paas koi xray waxray nahi hai

Interviewer: sahi

DM-03: woh bolti hai hojaayega hojaayega aesa hua hai

Interviewer: hmm toh TBA k us mein kia lagta hai kia ache comfortable lagta hai us k sath

DM-03: kis cheez ka

Interviewer: TBA jab aati hai to hap ko acha lagta hai sahi kaam kar rahi hain ya bura kaam kar rahi hain

DM-03: woh asaani se hojaata hai phir woh kehta hai TBA sahi hai nahi hoga phir bolega

Interviewer: to hap k sath kabhi is tarhan hua hai koi sahi waaqiya na hua ho

DM-03: idhar toh nahi

Interviewer: un sab k jitney bhi ap ne pedaaish deikhe hain ghar pe joh bhi huyi hain sab sahi huyi hain ? acha ap TBA ko jaante hain matlab sab unhi ko jaante hain

DM-03: han TBA toh paros ki hoti hai

Interviewer: paros ki hoti hai jee jee jee aur un k woh pehle bhi a chuki hain ghar pe kabhi

DM-03: pehle bhi

Interviewer: pehle bhi achuki hain sahi aur sab har dafa unk sath sahi gaya hai ilaaj aur sab kuch zachgi sab sahi gaye hain

DM-03: han

Interviewer: acha aur ap mawaazma kar sakte hain hispatal aur TBA k beech mein jaise ap ko behtar konsa lagta hai aur kyun

DM-03: nahi nahi hispatal ka toh behtar hai na woh xray wagera karte hain

Interviewer: hmm

DM-03: sahi

Interviewer: hmm sahi hai

DM-03: xray mein joh hota hai na

Interviewer: jee

DM-03: woh sahi hota hai na

Interviewer: jee sahi .. sahi hogaya aur ap ko koi hispatal ki koi cheez shayad achi nahi lagti ho koi bhi cheez ap bata sakte hain kyun k us cheez ko hum behtar karna chahte hain aur ap araam se bata sakte hain kisi ko pata nahi k kon bol raha hai

DM-03: nahi nahi sahi hai yeh hai k jab aurat ko khoon woon ka zaroorat hota hai toh un ko khoon deina chaiye

Interviewer: han jee jee toh woh hispatal mein hosakta hai

DM-03: han

Interviewer: sahi .. toh woh nahi deite khoon

DM-03: woh aese taareekh deita hai phir taal deita hai

Interviewer: hmm acha aur TBA ki koi cheez ap ko buri lagti hai ya sirf un ka acha lagta hai

DM-03: TBA bhi khud jaati hai udhar ap k center mein

Interviewer: hmm hmm hmm

DM-03: jaati hai woh taawan mein hai us ka

Interviewer: jee jee jee

DM-03: center se joh udhar se bolta hai woh udhar batati hai sahi

Interviewer: jee sahi aur center ki cheez shayad ap ko sahi nahi lage koi bhi cheez ap ne suni ho

DM-03: kia cheez ka

Interviewer: center ka … joh hamara center hai na

DM-03: han

Interviewer: us ki koi cheez shayad ap ko achi nahi lagi ho

DM-03: hum jaata udhar nahi hai

Interviewer: hmm

DM-03: hum ne suna nahi hai

Interviewer: suna nahi hai acha aur hispatal k bare mein kuch suna hai

DM-03: nahi nahi

Interviewer: nahi ?

DM-03: nahi

Interviewer: koi bhi buri cheez

DM-03: buri nahi suni

Interviewer: acha toh agar ap ko hispatal jana acha lagta hai toh phir ghar pe kyun us ko tarjee deite hain

DM-03: nahi nahi joh pehle zamaane k joh system chal raha tha

Interviewer: jee jee jee

DM-03: abhi woh ahiste ahiste khatam horaha hai

Interviewer: jee

DM-03: sahi hai

Interviewer: jee jee

DM-03: han khatam horaha hai abhi yeh hispatal aayi hai sahi hai

Interviewer: jee jee jee acha mujhe batao ap ki raaye mein ghar ki pedaaish ka jab taawan karte hain toh us mein aurat ki pasand aam hai ya phir ghar walon ki us mein raaye zaroori hoti hai ya us ko tarjee di jaati hai

DM-03: nahi nahi woh aurat bhi koshish karti hai k ghar mein hojaaye

Interviewer: acha

DM-03: bache waali aurat woh bhi koshish kar k ghar mein hojaaye ghar pe log bhi kyun k yeh nahi karte k hamara bacha zaaya hojaaye is mein kisi ka nahi hai

Interviewer: hmm hmm

DM-03: sahi hai isi mein kisi ka raaye nahi hai who bolta hai hojaaye ghar pe araam se agar ghar pe nahi hota hai toh hispatal mein sahi hai

Interviewer: matlab ap ki nazar mein woh khud apna decision le sakti hai

DM-03: han

Interviewer: is tarhan se woh ijaazat khud se apne

DM-03: hum toh joh bare hain woh ijaazat deite hain baaki joh naye larke hain nahi shadi karte hain toh woh bolte hain k abhi kia roz roz jaane center pe

Interviewer: sahi

DM-03: naye larke hai na

Interviewer: samjh gaya mein

DM-03: woh bolte hain rozana kyun center pe

Interviewer: sahi acha toh agle hamla pe agar ap ki yeh joh bahu ya koi bhi ghar mein kahe k hamein hispatal pe karna hai us ko udhar le jaayeinge

DM-03: han han le jaayeinge

Interviewer: le jaayeinge acha toh acha ap ki raaye mein is k baawajood jitni bhi hum ne saari baatein ki ap ko is mein abhi bhi yeh deakha jaata hai k aurtein ghar pe pedaaish karti hain toh is ki kia wajha hosakti hai ap ko kia lagta hai

DM-03: yeh pehle gaaon mein aesa hota hai na hispataalein dur thin na toh yeh gaaon mein hota tha abhi toh ziadatar hispatal mein hota hai

Interviewer: sahi

DM-03: abhi yeh hum ne muharram mein suna hai yeh idhar aage yeh k woh le k gaya tha do hamla larki

Interviewer: sahi

DM-03: us ko phir taareekh de raha tha taareekh de raha tha

Interviewer: taareekh?

DM-03: phir unhon ne private pe kar diya

Interviewer: acha

DM-03: private pe karwa diya

Interviewer: private pe kara diya toh phir kia hua us se

DM-03: hogayi larki paida

Interviewer: paida huyi bachi woh sehatmand hai?

DM-03: han

Interviewer: toh wohi hispatal pe gaye thy kahan peg aye thy woh kohi goth?

DM-03: kohi goth gaya tha phir bola abhi nahi hoga

Interviewer: hm

DM-03: 4 5 din aaye thy woh ruke nahi

Interviewer: hm

DM-03: larki k walidein thy na

Interviewer: hm

DM-03: woh bola dusri jagha se karwa lo

Interviewer: acha woh konsi jagha thi yaad hai ap ko koi

DM-03: yeh apna madam hai udhar

Interviewer: acha

DM-03: riya naam hai pata nahi

Interviewer: sahi acha acha acha

DM-03: jee

Interviewer: sahi toh acha toh ab mujhe poochna tha k ap hispatal ya center ka koi aesa masla hota hai k faasla bohat hai ya kuch sahi se baat nahi karte kuch ap ne suna hai in k bare mein

DM-03: nahi aesa nahi suna

Interviewer: faasle ka masla nahi hota?

DM-03: nahi nahi

Interviewer: aur agar le k jana parta hai toh gaari waari ka masla hota hai

DM-03: gaari kabhi toh le k jaata hai kabhi nahi bhi milta hai

Interviewer: jee jee jee jee sahi sahi

DM-03: us ko baat karo k le k jaawe phir le k aawe

Interviewer: han jee jee jee to hap k ghar mein joh saare bache huye hain woh sehatmand paida huye hain ya kuch

DM-03: ziadatar masla hai hamara khud ka sehadmand hai

Interviewer: jee jee jee aur koi

DM-03: baaki toh udhar ilaaj bhi hua hai ap k center pe

Interviewer: acha woh un ko sahi laga tha?

DM-03: woh bhi sahi tha

Interviewer: sahi laga tha acha aur ghar mein koi aesa hua hai k kisi ka bacha paida hua lekin woh zinda nahi tha

DM-03: aesa nahi hua

Interviewer: aesa nahi hua acha sahi hai

DM-03: thek hai aur bas?

Interviewer: bas aakhari do sawal reh gaye hain acha kareebi rishtedaaron mein kisi ka bacha zaya hua ho hamla k doraan

DM-03: hamare ko abhi yaad nahi

Interviewer: yaad nahi hai ap ko

DM-03: yaad nahi hua hoga shayad

Interviewer: hua hoga shayad jab bacha zaya hota hai toh ap ko lagta hai k kyun hua hai koi masla kia masle hosakte hain is k ya tashkeesh sahi nahi huyi thi kisi cheez ki is tarhan se ap ko lag raha hai

DM-03: shayad koi bacha kamzor paida hota hai ya us ki khoraak ki wajha se ho

Interviewer: hmm ap ko lagta hai k shayad pedaaish karne ki jagha pe masla hosakta hai is liye woh zaaya hogaya

DM-03: jagha toh ghar pe sahi hoti hai sab ka ghar toh ghar hai

Interviewer: jee jee jee sahi hai toh ap ko lagta hai jaise bache ki haalat thori si woh k us ko kisi cheez ki zaroorat ho hispatal ki toh ghar pe sahulat na us waqt maujood ho ap ko nahi lagta k behtari hogi k hispatal mein agar Allah na kare kisi k sath bhi hosakta hai agar koi is tarhan ka waaqiya ho

DM-03: nahi nahi hispatal behtar rahega

Interviewer: han na

DM-03: ghar mein toh phir nahi pohanch sakta hai na

Interviewer: pohanch nahi sakta hai na

DM-03: ilaaj behtar nahi hoga bache ka

Interviewer: hmm hmm hmm

DM-03: hispatal mein sahi hai

Interviewer: fori ilaaj ghar pet oh kuch cheezein is tarhan ki hoti hai k khoon charhaana hai koi antibiotic deini hai kisi bhi kisam ki toh woh ghar pea p ko lagta hai k woh sahulat foran de sakte hain ap

DM-03: nahi ghar pe nahi milegi

Interviewer: ghar pe nahi milegi ?

DM-03: han hispatal sahi hai

Interviewer: aur us ka hum andaaza bhi nahi laga sakte k pedaaish se pehle kis cheez ki zaroorat hogi kis ki nahi

DM-03: andaaza nahi hai

Interviewer: andaaza nahi laga sakte hai na

DM-03: nahi

Interviewer: toh agar yeh saari cheez rakhte huye phir ap ko kia lagta hai k ghar pe phir bhi pedaaish behtar hai

DM-03: nahi nahi ghar pe behtar nahi hai tabhi toh jata hai hisptal

Interviewer: hm acha mujhe poochna tha k ghar mein ap hi faisle karte hain jab bhi kahin ilaaj k liye jana ho bache ki sehat kharab horahi hai aurat ki sehat kharab horahi hai ap k ghar mein

DM-03: hamari larki mashallah jawan hai na

Interviewer: hm hm hm

DM-03: woh khud karti hai

Interviewer: acha

DM-03: hum apna khaam karte

Interviewer: jee jee jee toh faisle agar kon jaise ap ki bahu jis se hum ne baat ki hai toh un k liye faisle kon kar raha tha

DM-03: yeh toh abhi joh pehla joh hota hai na hamare joh pehla hi tha na is ka bacha

Interviewer: hm hm hm

DM-03: un k waaldein mein paida hua

Interviewer: acha

DM-03: larki k waaldein mein

Interviewer: acha

DM-03: pehli baar udhar hota hai

Interviewer: jee jee jee jee

DM-03: saatwein aathwein mahiney mein na

Interviewer: hm hm hm

DM-03: waaldein k paas chala jaata hai

Interviewer: acha toh paida bhi udhar hi hua tha bacha un k ghar

DM-03: udhar hi hua tha bacha

Interviewer: udhar hi hua tha toh un ka saara faisla waisla un ka

DM-03: han un ka hota hai

Interviewer: acha acha acha

DM-03: dusra bacha abhi shuru hoga abhi joh hoga na

Interviewer: ab dusra bacha (laugh)

DM-03: phir zindagi bhar idhar hi hai

Interviewer: inshallah inshallah

DM-03: thek hai

Interviewer: sahi hogaya

DM-03: pehle joh hai na pehla bacha joh hai shadi hota hai

Interviewer: hm

DM-03: shadi k saatwein aathwein mahiney waise toh un ka likha hua hai saatwaan mahina

Interviewer: hm

DM-03: kisi ko woh bahu bolegi k hum idhar hi sahi hai

Interviewer: jee jee jee

DM-03: toh aathwaan mahina nauwein mein zaroor jaayegi

Interviewer: acha sahi hai

DM-03: phir baaki zinadgi hai joh woh sasuraal mein

Interviewer: jee sahi sahi sahi acha aur koi cheez hai joh hum ne poocha na ho hispatal ya pedaaish ya ilaaj k bare mein joh ap batana chaahein aur koi bhi cheez hamein behtar karni chaiye

DM-03: abhi toh log ap k center aate hain

Interviewer: jee

DM-03: toh taawan kiya karo khoon ka khoon diya karo gaari ka masla hai udhar pohchana hai abhi toh kohi goth pe jaraha hai ziada

Interviewer: jee bilkul

DM-03: toh idhar bhi beitha hai attiya hai

Interviewer: jee jee

DM-03: idhar bhi hum ne suna hai

Interviewer: jee jee jee k

DM-03: aakhari mein _________(17.42-17:46)

Interviewer: jee

DM-03: baaz jagahon pe ap ka taawan sahi hai baaki aese din ajaate hain

Interviewer: hm hm hm sahi

DM-03: is pe bhi ap log sahi karoge time muharram ho ya muharram ka dasween

Interviewer: han har waqt har waqt chalna chaiye

DM-03: eid ho us pe bhi ap jaari rakho kaam

Interviewer: bilkul isi liye joh ap se bhi raaye le rahe hain na usi pe kaam sehat pe kaam kar sakein aurton aur bachon pe

DM-03: nahi nahi ap ka kaam sahi hai

Interviewer: bohat shukriya ap ka waqt ka ……………..

____________________________X___________________________

IDI DM-04

Interviewer: kya umar hai apki

DM-04: 30 saal

Interviewer: kitni taleem hasil ki huwi hai ap ne?

DM-04: nahi ki

Interviewer: ap Woman-04 ke kon hain?

DM-04: shohar hun

Interviewer: mujooda mulazimat kya hai apki?

DM-04: company mein kaam karta hun

Interviewer: mujhe bataaein jab ek aurat hamal se hoti hai to kin cheezon ki zarooriyat hoti hai?

DM-04: sir hamal ke doraan sab thek hota hai bas delivery k doraan aurat ko kamzori ki waja se dard to shuru hojata hai delivery ka lekin kamzori se bacha nazar nahi aata k abhi kaise hoga wo ek cheez bohat pareshan karti hai jo ke shayad achi giza na milney ki waja se hoti hai kuch ham kama nahi paatey jese hum 2 bhai kamaney wale hain or ghar mein 18 afraad to wo cheez hamari aurton ko dekhni parti hai phir to Jahan bhi hospital mein le kar jatey hain to yehi khete hain ke khoon ki kami hai or naseeb ki bat hoti hai kisi ko mil jata hai khoon kisi ko nahi milta to hamey khud dena parta hai , mene khud apni behan ko khoon diya hai 2 baar khoon. To meri nazar mein hamarey maashrey mein khoon ki kami ziada tar aurton ko hojati hai na khaaane peene ki waja se. dusri saholiyat nazar to aati hain lekin wahan janey ke baad wo kam hojati hain jese apko andar jane nahi dete , KGH mein 2 ladies hein yahan se le kar gaya hun to meinn bahar wait karta hun or aurten andar ab unka tamam kharchiyaat wagera mere paas hai or mein hi bahar hun or wahan andar ladies ko bol rahe hain ke ye lao wo lao phir mein gate se de raha tha to wahan ye security ka masla hai or center mein khoon ka masla hai wahan khoon nahi milta to ye ek cheez hai gareebi ki waja se nahi ho pata pura nahi ban pata aurton mein khoon to is cheez mein ap log bhi kuch support karenge to hamarey bachey jo hain wo normal paida honge , ap wahan jaa kar khoon mangtey ho is se bhetar yahan aa kar khoon ki bottle chara do.

Interviewer: to ap ke nazar mein hamal ke doraan achi khoorak or khoon ki bottles charna zaroori hai?

DM-04: Jee han.

Interviewer: is ke ilawa koi or cheez jo apko lagta ho hamal ke doraan zaroori hoti hai?

DM-04: or to ye k center wale dete hain bachey ya maa ke khane k liye jab ke abhi tek hamarey yahan kuch TBA hoti hain jo kheti hain ye nahi do wo nahi khilao to kuch maaon ki bhi galti hoti hai ke wo unka kaha manti hain jab apko ek parhi likhi madam samjha kar bata kar gai hai ap usko nazar andaz kar rahi ho or TBA ki sun rahi ho to ham ek is cheez par chalen kiyu ke ye science hai or ye aage barh chuka hai Ma Sha Allah to ab hamey inki sunni chaiye lekin kuch log abhi bhi wahin ruke huwe hain.

Interviewer: acha ye bataaein kya apko lagta hai jo test wagera hote hain hamal ke doraan wo bhi zaroori hote hain? ultrasound wagera?

DM-04: jee zaroori hote hain

Interviewer: or jo cheezen center mein faraham ki jati hain wo apki nazar mein zaroori hain? aap aa chuke hain center par?

DM-04: hum mard nahi jatey wahan normally qareeb hi hai to itna to aurton ko bhi nikalna chaiye ke wo khud chali jaen

Interviewer: acha ap keh rahe thy ke TBA ko bula lete hain , to unhain kon bulata hai

DM-04: TBA meri saas hai

Interviewer: agar ap kahain ke mein apna bacha hospital mein karwana chata hun lekin wo kahain ke nahi ghar mein bhetar hai to asa hota hai ase mukhtalif khayalat aate hain?

DM-04: jee aate hain kiyu ke hum log gareeb hain hamarey paas itne ikhrejaat nahi hote ke hum foran se gaari le aen unhain le jaen hospital mein rekhain khilaen pilaen , islye hum chate hain ke kuch yahan par (during pregnancy) kharch karen un par taa ke normal hi ghar mein inka hojay or ye yahan par safe rahain or hum bhi jaa kar kama seken or hum tasalli mein rhete hain ke meri biwi to ghar mein hai mein jaa kar kaam kar sekta hun to yehi meri khuwaesh hoti hai lekin nahi majboori mein jo hai wo hospital jana parta hai meri 800 rupee ki dihari mar jati hai pareshani mein hospital jana parta hai peechey peechey call aajati hai ke ye masla hai wo masla hai to ye pareshaniyan jo hain wo thori tang karti hain to aap yahan test karwao khoon charao baaqi to Ma Sha Allah sab hota hai madam aati hai dekhti hai btati hai is mein khoon ka masla hai ab ye puriya khao ab wo karo lekin bas jab ye paida karney ka masla aata hai tab bas ye khoon ke masley par mein keh raha hun bas.

Interviewer: to apki raay mein ghar mein hona chaiye ya center mein?

DM-04: shuru shuru mein to ghar mein hote hain na , ye to science or waqt asa hai ke hospital mein ho rahe , jab ke qudrat to yehi chati hai na ke ghar mein ho normal ho lekin gareebi wahan center mein le jaati hai lekin agar han wahan doctor hum gareebon se kuch na mangen bas delivery karen ke Mubarek ho bachi huwi hai ap ke lekin us phele ye khete hain k khoon chaiye hamy khoon ki zaroorat hai aap apney bhai ko le ao ap apne falan ko le ao mein apni bhanji ko de chuka hun khoon apne bhatijey ko de chuka hun , mein apni beti ko kahan se dun ab meinn nahi de pa raha ab jab ke aulaad to meri yehi hai lekin abhi pichley hi mahiney mene dya to ab kaise dun jab ke usey zaroorat hai khoon ki to yehi ek cheez mujhe sahi nahi lagi baaqi to mujhe koi shikayat nahi hai .

Interviewer: hmm aap ki raai mein pese bohat lag jatey hain is mein kharcha bohat aajata hai?

DM-04: jee jee

Interviewer: is ke ilawa koi masla faasley wagera ka aane jane ka?

DM-04: jee nahi wo to gaari aati hai unki (center ki)

Interviewer: time par aajati hai?

DM-04: jee jee aajati hai , aaj bhi ap dekhain diwaron par number likh kar jatey hain wo ke jab bhi apko zaroorat ho aap bula len , meri bhatiji bemaar thi mene call ki to wo log aagay gaari ke samet mujhe hospital le gay admission karwaya bacha ward mein or kaha ke ap mamoo hain to ap ruken behan ke saath or koi masla ho to humey call kar di jyega. Ap yekeen karen mein wahan tha sir to mujhe doctor ne kaha ke is ke reerh ki haddi mein injection lagega jab ke bachi kamzor si thi , paros mein ek Khatoon thin unhon ne pucha ke ye bachi seh paegi injection? nahi seh paegi mar jaegi. Doctor ne kaha iska paani nikalna parega. To mene kaha nahi mein raazi nahi hun bachi kamzor hai usko injection laga kar maarna achi baat nahi hai , center wale to hamey chor kar chale gay apne upper se hata diya ab mein soch raha tha kya karun kya nahi mene raat ko 3 bajey chutti leli doctor ne bhi nikal diya kaha ke ap farigh ho bachi ko discharge kar dya tha ab mein raat k 3 bajey hospital se kaise ghar aonga gaari bhi nahi milegi mujhe to kher mein raat bhar idher udhar hota raha ke raat guzrey phr subah doctor ne mujhe itna bara ek parcha bhar kardya ke ye le jao mujhe English nahi aati thi wo mein laya to hospital wale mujhe bol rahe ke is mein likha hai ke tum ne badtameezi ki hai tum ne mana kara ye wo mene kaha mene to kuch nahi kya mene bas inkaar kya tha ke injection nahi lagao ke ap 2 ghantey dekho kuch or karo lekin injection nahi lagao , mene wo jo bajiyan aaen thin unhain bhi yehi kaha ke mene bas mana kya tha , aj Allah ka shukar hai wo bachi 6, 7 saal ki hai mein le aya tha subah subah bas Allah ka saharey ke bachi mar rahi hai mein injection nahi lagaonga. Phir center wale bhi ay thy 2 din dekha tha bachi ko kaha the k hai or wohi hospital mein phaink kar ay thy unhon ne hi 2 din rekha bukhar utar gaya to dedya to phele kiyu chor kar aaye thy hospital.

Interviewer: acha ye bataaein delivery ke aen waqt kis cheez ki zaroorat hoti hai apki nazar mein?

DM-04: usi waqt sir yehi hota hai ke mareez mein jaan nahi hoti kamzrori hoti hai , kuch pedaaishi kamzor hoti hain kuch ghr mein shohar wagera khayal nahi rekhtey us par phir bacha to wo to khoon mangega naa jo government hospital mein mangtey hain pese wese nahi mangtey lekin ye sab ..

Interviewer: to khoon ki zaroorat hoti hai?

DM-04: jee

Interviewer: ap ke qareeb koi ghar par pedaaish ka koi acha ya bura waqiya ap batana chaen jo ap ne dekha ho apne aas paas?

DM-04: jee ghar par to asa koi waqiya nahi bas jo apko btaya apni bhanji ka ye log hi kuch kar sektey thy lekin nahi kya wahan hospital ma chor kar aagay bad mein bhi unhi ne thek kya to kuch galtiyan mein samajhta hun doctors ki bhi thin ke wo samajh nahi us waqt mayus kya tabhi aage jane ki zaroorat pari phir bad mein kisi or doctor ne usi jaghan dekha to bhetar hogai.

Interviewer: to tamam bachey ghar mein asani se hogay thy?

DM-04: jee han , meri begum mujh se 2 saal bari hai or jaan mein bhi hai phir wo thori hushiyar bhi hai mein Allah ka shukar ada karta hun is par ke mujhe thori hushiyaar begum mili hai har kisi ki aurat asi ho to wo shohar ko bhi samjhey , aurton ko ghar mein maartey hain to wo bhi apni ehmiyat nahi samajh paati phi rase bachon mein sehat ke masaail bhi aatey hain to mein samajhta hun kuch hamari bhi galtiyan hain hm ap jeson se suntey nahi hain samjhtey nahi hain to hamey koi samjhaney wala ho ham samjhain ase hi hum mein taleem nahi hai hamara koi bacha parh raha ho parhtey parhtey sahi hojay phir ek sham kahin chala jay kuch pese kama le to bas usey dusrey din bhi kahainge ke tu kaam par chala ja taa ke wo 4 pese kama le to mein manta hun ke muashra bhi hamara kuch sahi nahi hai lekin aj kal

hospital mein bhi ap ruko to subah mein koi or doctor kuch or likh kar chala jata sham mein koi kuch or to mein kya karun ab beech mein jese mera chacha 2 mah se hospital mein hai mein us ke saath 8 din ruka subah mein alah team aati hai kuch log ache bhi mil jatey hain ache se samjha kar jatey hain lekin kuch khud bohat pareshaani mein ghum rahe hote hain har mareez ke sath unki lagi hoti hai kuch mareez bhi ase aate hain nafsyati jo unhain tang kartey hain .

Interviewer: ap ke sarey bachey hospital mein huwe hain?

DM-04: 2 betiyan hospital mein huwi hain or 3 normal ghar par huwe hain.

Interviewer: acha hospital kyu le jana para?

DM-04: center se hamey hospital bheja tha wahan ek beti mein to bas gari se nikala tha table par letaya ke normal hogai Ma Sha Allah se.

Inteviewer : asa kyu ke kuch bachey ghar huwe kuch hospital mein?

DM-04: bas phele haalat bhi kuch sahi thy gareebi bhi bohat si cheezen le aati hai ab 4, 5 saal se haalat kuch kharab huwe to uska bhi asar ek aurat par aata sehat par wo khayal nahi rekh pati apna to wo asar bachey par bhi parta hai phir.

Interviewer: ap ke ekhri bachey ki pedaaish ghar par huwi hai?

DM-04: jee

Interviewer: or jo hospital mein huwe wo kis waja se huwe normal nahi thi ya kya waja thi?

DM-04: nahi normal to thi wo us mein bas jaan nahi thi jab ek cheez samney saholat thi tekleef mein to wo kyu nahi istemal karey.

Interviewer: to jo ghar par huwe wo kharchey ki waja se?

DM-04: jee

Interviewer: ap ke nazdeek ghar mein bacha karna bhetar hai ya hospital mein jese apki bhain ke sab bachey ghar mein huwe or biwi ke kuch hospital mein bhi huwe to kis ka ziada behtar raha?

DM-04: biwi ka kyu ke meri biwi thori hushiyar bhi hai or hushiyaar insan pareshani mein shohar ke bhi kaam aati hai. Sabko asi hushiyar biwi mili to bachey bhi ache se ho or muashra bhi acha hojay.

Interviewer: apki raay mein bachey ki pedaaish ka decision aurat khud le yaa kisi se ghar mein pooch kar le?

DM-04: nahi wo khud faisla karey kyu ke dar wagera to usey dekhney partey hain naa.

Interviewer: agar wo hospital jana chaen ya ghar par karna chaen to jo unki marzi?

DM-04: jee jee

Interviewer: apki raay mein ghar mein asi konsi saholiyaat hoti hain jiski waja se wo ghar par karna pasand karti hain kya waja ho sekti hain?

DM-04: is ke lye apna khaal rekhna parta hai bolna bhi parta haia pni saas shohar ko ke mein khayal rekh rahi hun apna ya mujhe kuch acha khilao to ye sab cheezen wo khud karengi na apne andar taqat ke lye. Kuch saholiyaat ki waja se bhi ab foran chale jate hain ke saholiyaat nazar aa rahi hoti hain islye

Interviewer: acha apki saas jo hain wo TBA hain wo kisi or ke bhi bachey karwati hain to koi kisi ko masla to nahi hota?

DM-04: nahi bohat kam wo ghusti hain ase mamlaat mein wese to bht sarey case unhon ne yahan normal karway hain lekin ab ziada tar wo khud center bhej deti hain logon ko.

Interviewer: apka bohat shukrya ap ne bohat achi chezen batai hain is par hum kaam kar sektey hain

DM-04: madam sab se bara masla yehi hai khoon ka hamare yahan achi giza nahi hai aurton ko pata nai hai ke hamal ke doraan kya cheezen khaen jis se khoon barhey

Interviewer: acha koi or asi baat yaa cheez jo bachey ki pedaaish k mutaliq ap batana chaen jo hum na puch seken jo?

DM-04: jee nahi bas mere nazdeek yehi sab jo mene apko bataya.

Interviewer: ap ne bohat achi maloomat hamey di In Sha Allah hum is par kam karenge zaroor apka bohat shukrya hamey waqt dene ke liye.

______________________________X____________________________

IDI DM-05

Interviewer: apki umar kitni hai?

DM-05: 50 to hogi

Interviewer: apki taleem kitni hai?

DM-05: Quran Paak parha hai

Interviewer: ap Woman-05 ki kya hain?

DM-05: saas

Interviewer: ap abhi koi kaam karti hain ya ghar mein hi hoti hain?

DM-05: ghar mein hi hote hain maesaley banate hain garam masala mein or meri bahu wagera baqi ghar ke kaam hote hain or wo maesala banana hota hai bas

Interviewer: apki raay mein hamal ke doraan ak aurat ko kin cheezon ki ziada zaroorat hoti hai kon si saholiyat ki?

DM-05: aj kal dekhain bachiyon ko zehni sukoon chaiye hota hai jese ghar mein saas hoti hain unka rok tok karna ye karo wo karo to ye bohat bari uljhan ban jati hai tension ho jati hai ase larkiyon ko to ye nahi hona chaiye mein bhi apni bahuon ko tension nahi deti or bhi balkey agar mere beton ki taraf se kuch ho to unhain dant'ti hun mein

Interviewer: or kis tarhan agar tab zarooriyaat poochi jaye jese khana wagera kin cheezon ki ziada zaroorat parti hai?

DM-05: jesa khana jo dil chata hai wo laa kar dete hain kuch bahar ka khana ho to wo bhi manga kar dete hain kuch ghar mein pakana ho to wo bhi dete hain jo jesa inka dil chata hai wesa kartey hain

Interviewer: or jis waqt bacha paida ho raha hota hai us waqt apka kya khayal hai?

DM-05: khayal bas yehi hota hai ke jo Allah ki marzi bas meri bahu kher kheriat se ho mein in ke saath hoti hun donu bahu ke

Interviewer: or sehat ke hawaley se sab se zaroori apko kya cheez lagti hai?

DM-05: sab se zaroori bp lazmi check hona chaiye kiyu ke aj kal ki larkyon ko kisi ko high rehta hai kisi ko low to wo zaroori check karwana chaiye jese thori si inki tabyat kharab hoti hai to mein bolti hun ja kar check karwa kar aajao doctor bhi kardete hain bp to check to hum kara lete hain

Interviewer: to hamal ke doraan ap samajhti hain ke sab se zaroori yehi hai?

DM-05: jee bp check karna zaroori hai jese kabhi koi ghabrahat ho rahi ho ya chakar aa rahe ho to bp check krwane ka hi bolti hun mein

Interviewer: or apki raay mein sab se bhetreen jaghan konsi hai Jahan bacha paida karna chaiye?

DM-05: meri raaye mein to hospital sahi hai kyu ke wahan har cheez ki saholat mujood hoti hai or aj kal ki larkiyan kamzor hoti hain ye jese inka jo mashwara tha jab mene pheli dafa btaya kyun ke saholat to yahan bhi hoti hai ye in ke paas hamare to sare bachey inhi ke pass huyehain ye bhi family doctor hain lakin in ke paas saholat ye nahi hoti jese sans kam hota hai to wo machines wagera nahi hoti in ke paas ye hospitals mein hoti hain or isi ehtyat ki waja se mein apni tamam bahu ka naam likhwa deti hun meri bas koshish hoti hai ke koi masla na ho jo bhi masla ho us ke liye hum tayar hone chaiyen hain ab ye bahu to bachiyan hoti hain inhain kya pta hota hai ye sab to saas ko pata hota hai usey tajaruba hota hai kyun ke usi ke naqsh e qadam par bahu ya beti chlti hai isi lye saas hi karti hai

Interviewer: koi aesa tajaruba ya kuch kabhi aesa dekha ap ne ya suna jiski waja se bacha paida karney ki jaghan ke mutaliq apki raay badli ho?

DM-05: nahi mujhey to bas family clinic sahi lagta hai ya phir hospital mein ye jo TBAs hain aj kal inka kam nahi hai

Interviewer: ap ke bachey bhi family doctor ke pas huyehain?

DM-05: jee mere do chote bachey jo ab 17 or 15 saal ke hain wo un ke paas hi huyehain.

Interviewer: to apka tajaruba udhar bilkul sahi gaya?

DM-05: jee bilkul sahi gaya hamare muhalley ke jitney case hote hain meri nanden dewraniya sab idher hi jati hain ye family doctor hain hamari

Interviewer: apki nazar mein larki ki raay bhi zaroori hai bachey ki pidaesh ki jaghan ke hawaley se?

DM-05: jee zaror mein bilkul puchti hun us waqt eri nahi iski raay zaroori hoti hai kyu ke isko ziada bhetar pata hoga ke meri halat kya hai kesi hai to iski mujhey sunni chaiye us waqt

Interviewer: abhi to ap keh rahi thin in larkyon ko kahan kuch pata hota hai…

DM-05: nahi andar ki jo halat hoti hai wo condition to ye log hi ziada bhetar janti hain na mein bas apni raay jo hoti hai wo btati hun

Interviewer: ap ke khayal mein log ghar mein bacha kyu kartey hain kya wujoohat hoti hain ya jo ghar meinn nahi kartey wo kyu nahi kartey?

DM-05: mere 8 bachey hain Ma Sha Allah se to shuru ke 6 wo ghar mein hi huyehain mere to hamari ak rishtey daar doctor hain wo chotai sei hospital mein kaam karti hain aur wo ghar par aa kar case karti thin to mera kabhi daiyon se wasta nahi para

Interviewer: theek hai or apko kyu lagta hai ke log hospital kyu nahi jate or ghar mein ya qareeb se ilaaj ya bacha karwatey hain?

DM-05: shayad paise bachane ki waja se ye khayal nahi rehta ke kuch bhi ho sakta hai bacha pedaaish ke waqt ulta bhi ho sakta hai sans bhi kam ho sakti hai kuch bhi ho sakta hai islye ye bewaqufi nahi karwani chaiye ghar mein andar ki ya to koi tajrube kaar ho doctor ho to be shak ghar mein bhi aa kar kardey to phir wo injection lagay ya goli rakhey jo bhi karegi theek karegi

Interviewer: apki raaye mein wo konsi rukawaten hain jinki waja se aurten hospital nahi jatin?

DM-05: mere khayal mein to paise bachaney ki waja se ya bahu ko bahu nahi samajhte honge ke ye bhi kisi ki beti hai insan hai khayal na rakhney ki waja se shayad

Interviewer: peso faasla wagera ke ilawa apko lagta hai or kuch rukawat ho sakti hain?

DM-05: jee nahi

Interviewer: kabhi ap ne aesa kuch suna ya dekha kisi ka aesa case jiski waja se wo hospital se dar gay hun bacha paida karney ke hawaley se?

DM-05: jee nahi mene nahi suna

Interviewer: or apki dusri bahu ka tajaruba kesa raha family clinic mein karwane se?

DM-05: is ke bachey pidaesh ke waqt ultey hojatey hain to mein le gai thi isey family doctor kei paas unho ne kaha ke is mein himmat hai to mein karwa dungi yahan phir is ne kaha ke ammi mein yahin karwa lungi to phir wahin clinic mein karwaya mene iska

Interviewer: to apka tajaruba kesar aha wahan?

DM-05: acha raha lakin ase bacha ulta ho to hospital mein hi karwana chaiye

Interviewer: aesa kya huwa tha ulta bacha hone par?

DM-05: nikaltey huyebachey ki paon ki nas dab gai thi panjey ki

Interviewer: yahi huwa tha family doctor ke pass?

DM-05: jee ab to sahi hogya chalta bhi hai 3 saal ka hogya

Interviewer: to apko aesa lagta hai ke agar hospital mein hoti to ziada bhetar hota?

DM-05: jee mujhey lagta hai ke hospital mein hoti to ye haddi ka nas ka bhi checkup wagera kartey huyeultrasound wagera se unehn pata chal jata to wo iska bhi ilaaj kartey ab ye family doctor hain to achi lakin bad mein phir jo bhi hota hai ye kheti hain hospital jao ya Jahan jana hai wahan jao to bas wo to bacha paida karwati hai

Interviewer: to wo bhi ultrasound karti hain?

DM-05: nahi ak dusra doctor ka clinic hai wo wahan se karwati hai wahin ka mangti hai ab pata nahi rishtey daar hain ya kya lakin wahin ka mangti hain phir wo dekh kar bata deti hai ke mein karwa dungi bacha ya kabhi keh deti hai ke mein karwa to dungi lakin larki ko himmat rakhni hogi ye wo to phir is tarhan mein nahi karwati phir

Interviewer: center kin cheezon ke lye jati hain ap?

DM-05: checkup ke lye , ye din bohat ahem hote hain to udhar jatey hain to wo wahan test wagera se sab pata chal jata hai ke khoon ki kami hai ya nahi acha normal hai ya nahi hai or agar normal ki bol dete hain hospital wale hi to phir hum idher hi karwa lete hain nahi to phir hospital jan parta hai

Interviewer: acha Jahan se aap karwati hain ultrasound wahan kitney paise lag jate hain ap ke?

DM-05: 450

Interviewer: or hamare yahan to free hota hai

DM-05: jee wahan bhi jate hain bas 3 mahine to pata nahi chalta us ke bad zara bhi kuch tabyat kharab hoti hai mein intezar nahi karti mein khud le kar jati hun mere beto ya shohar ko to pata bhi nahi hota , ye din bohat ahem hote hain in mein gaflat nahi karni chaiye

Interviewer: apko hamarey center ka kuch bura lagta hai?

DM-05: abhi hum ne kabhi wahan case karwaya nahi hai to pata nahi hai meri dewraani ne karwaya tha to uska bilkul theek raha

Interviewer: ap ke khayal mein kuch aesa jo ap puchna cha rahi hun ya hum ne nahi poocha ho?

DM-05: jee nahi sab sahi hai

Interviewer: jee okay

____________________________X___________________________

IDI DM-06

Interviewer: apki umar kitni hai?

DM-06: 55

Interviewer: ap ne kitni taleem hasil ki hai?

DM-06: nahi kibas Quran Paak parha hai

Interviewer: or ap kuch karti hain kaam ya ghar mein hoti hain?

DM-06: jee ghar mein hoti hun

Interviewer: or apki nazar mein hamal ke doraan ak aurat ko kin kin cheezon ki zaroorat hoti hai?

DM-06: dawaai wagera or khane peeney se taqat wali cheezein baaqi ye 7 mahine mein jo pani chutta hai mujhe nahi pata kyu hota hai ho sakta hai bacha daani mein kharabi ho pehle achey se meine kaha ke shayad tum giri hogi washroom mein lakin is baar to yahan palang par lete lete paani chut raha tha

Interviewer: hamal ke doran sehat ke lye kya cheez zaroori lagti hai?

DM-06: bas usi time koi phal fruit koi taqat wali cheez koi dawaai dedein

Interviewer: or jis waqt bacha paida ho raha hota hai us waqt kis cheez ki zaroorat lagti hai apko?

DM-06: usi waqt bas pani ya khoon ki kami ka pata hona ab hume to nahi pata chalega na ke kya masla hai to wo doctor bta dete usi waqt hospital mein

Interviewer: jesa ap ne kaha ap ke apne sab bachey ghar mein huwe hain to ap ka khayal kya hai bachey paida karney ke liye konsi jaghan bhetar hai?

DM-06: hospital hi ghar par sahi nahi hai hamara time or tha abhi kuch or hai us waqt doctor or dusri saholiyat nahi thin

Interviewer: ap ke tajarubey mein koi achi ya buri cheezein huwi hain ya ap ne kisi se kuch suna ho?

DM-06: nahi aesa kuch nahi bas jo huwa wahin se theek hogya sab hospital ki bohat saholat hai bas ye akhri bachi mein bahu ki thori mushkil huwi bas lakin ye free hospital ham gareebon ke lye bohat zaroori or acha hai

Interviewer: jese ap keh rahi thin ke hospital mein ap ke ziada tar ache tajrube rahe hain or bas is dafa bura raha?

DM-06: jee bas is dafa gari nahi bhijwai thi unhon ne or kuch ilaaj bhi theek se nahi kya tha or ache se paish bhi nahi ay the jab bacha hogya us waqt call ki tab gari aai thi

Interviewer: ap ki raay mein ghar mein bachey ki pideash ka faisla kon karta hai ya kisey karna chaiye?

DM-06: bas doctors se karwana chaiye or hospital jana chaiye or aurat ki raay bhi aeham hoti hai lakin hum wohi karte jo doctor khete

Interviewer: ap ke khayal mein ghar mein bacha karney ki wujoohat ho sakti hain?

Interviewer: bohat si aurten kehti hain ke hum hospital nahi jaenge aesa kyu apki raay mein?

DM-06: bas takleef nahi uthane ki waja se rush or line wagera ki waja se mein to apni bahu ko kehti hospital jao line mein lago sab karo

Interviewer: center mein apko sab kaisa lagta hai?

DM-06: jee jee yahan sab acha hai taesalli bhi dete hain sab acha hai

Interviewer: or koi waja apki nazar mein jiski waja se aurten hospital nahi jana chati ya nahi ja pati

DM-06: bas takleef nahi uthani hoti line mein lagna gari mein aana jana intezar karna lakin takleef ke bager to kaam nahi hota na lakin koi koi banda sochta nahi hai ye sab

Interviewer: mein puchna cha rahi thi ke akhri bachey mein ap logon ka tajruba theek nahi raha to agli dafa kabhi ap center mein aana chaengi?

DM-06: jee jee shayad koi hamari galti bhi hai lakin mein hamesha wahin le kar jaongi

Interviewer: hum khush hain ke ap center se mutmaen hai or bhi hum bat karenge ke inka ache se ilaaj karen

DM-06: jee mherbaani hogi abhi tak iska khoon band nahi horaha

Interviewer: ak dafa ap wahan jaa kar apna masla unhain bata dein ap

DM-06: jee bas gaari ka masla hai itna door kese jaen bike par bachi choti hai ye bhi nuqsan hoga phir mere lye

Interviewer: or koi asi cheez jo ap batana chahein ya hum ne nahi poochi ho?

DM-06: nahi aesa kuch nahi sab hogya

Interviewer: chalen apka bohat bohat shukrya baaqi ak dafa ap inhain center le jaen taa ke inka ilaaj bhi hojaye or ap ke waqt ka bohat shukriya…

_____________________________X____________________________

IDI DM-07

Interviewer: acha DM-07 mein ap se poochna chah rahi hun k ap ki umar kitni hai

DM-07: meri umar ka andaza khud nahi hai k kitni hai

Interviewer: sahi hai acha waise takreeban kia lagta hai

DM-07: takreban itni hogi 30 hogi ya 35 hogi

Interviewer: sahi sahi sahi aur ap ne kitni taleem hasil ki hai

DM-07: taleem nahi hasil ki

Interviewer: thek hai aur ap k in k sath kia rishta hai

DM-07: is ki mein waise toh is ki shohar ki mein behan hun bari toh ami fout hogae to us k baad us ko mein ne paala hai

Interviewer: sahi sahi

DM-07: khala bhi hun aur matlab nand bhi hun

Interviewer: thek thek thek acha aur ap kaam karti hain ya ghar pe hi hain

DM-07: kaam karti thi

Interviewer: kaam karti thin?

DM-07: pehle kaam karti thin poori zindagi kaam kiya hai

Interviewer: jee jee

DM-07: jaaru poucha kaam pehle jaaru pouche ka kaam karti thi banglow pe phir baad mein idhar (community) mein aaye mein yahan pe government center hai bachon ko katre pilaane k yeh teekey us mein kaam dila us mein matlab 2 3 saal kaam kiya phir mein ne wo chor diya baad mein band hogaya tha phir chor diya phir dobara phir mein pehle waale seithon k paas chali gaen banglow pe kaam kiya yeh jaaru poucha bartan dhona aur abhi mera nikaah hogaya hai in ki shadi kar di baad mein mera nikaah hua bare bhai ne karwa diya abhi shohar nahi chorta

Interviewer: acha sahih ai acha toh ap k raaye mein janna chah rahi hun k aurat ko kia sehat ki saholiyat zaroori hoti hai hamal k doraan

DM-07: khaane peene ki giza bohat achi honi chaiye us ka khud ka bhi khayal rakhna chaiye achi achi cheezein deinge toh taqat aayegi bache paida karne k liye yehi toh matlab hamara tajaruba hota hai

Interviewer: jee jaise k hispatal mein kia saholiyat hai

DM-07: hospital mein jaati hun matlab ache se deakh bhaal ho samjhana chaiye phir us ko baad mein matlab achi wali dawaai jis cheez ki us ko zaroorat ho wo deini chaiye

Interviewer: jee sahi acha toh delivery k time pe kia saholiyaat aur khidmaat ki zaroorat hoti hai

DM-07: delivery k time pe matlab abhi pehli dafa horaha hai us ko samjh nahi hai toh un ko chaiye un ko thora samjhana chaiye pyar mohabbat se patient k sath sahi pesh aana chaiye toh us ko samjhana chaiye aur wo toh karte nahi hain faida kia dawaai ki zaroorat hai dawaai dou us ko deakho kia cheez ki zaroorat hai wo toh nahi deite hain bs apne kaam karte rehte hain

Interviewer: hm hm hm hm aur delivery k time pe jaise kuch kehte hain k wo machine bari bari machine hoti hai aur kia saholiyat ki zaroorat hoti hai

DM-07: drip wagera charhaate hain taqat ki agar khoon ki zaroorat hoti hai toh khoon ki bhi drip charhaatey hain jis cheez ki zaroorat hoti hai doctor ko hum se ziada pata hota hai lekin yeh log toh nahi karte wo cheezein mein ne apni bhabi ko le k gaye thy na JPMC mein us ko delivery nahi huyi thi khoon ki kami arahi thi un logon ne bola khoon ka intezaam karo agar ap k yahan se hota hai toh sahi hai nahi toh kahin se leina parta hai

Interviewer: hmm

DM-07: hum ne kaha hamara banda hai de deiga toh hum ne bulaya us k shohar ne apni biwi ko khoon diya usi waqat khoon diya usi waqat charhaaya usi waqat delivery normal hogayi

Interviewer: hm hm hm sahi aur jaise agar ghar pe ilaaj kar rahe hain TBA ko bula rahe hain toh wo kia saholeeat deity hai

DM-07: wo yeh deity hai ke matlab bache ko dekhti hai aurat ko pyar mohabbat se pesh aati hai agar zaroorat hoti hai phir hum bhi itne toh na samjh hum bhi nahi hai thora bohat tajaruba hai hum deakhte hain kw agar yeh case solve kar sakti hai hum us ko ziada time nahi deite hain us ko 10-15 min deite hain jab case solve hota hai toh sahi hai agar nahi toh hum private hi mein le k jaate hain toh yeh bolte hain ziada hamare mareez k sath matlab khan k totkey wagera nahi karte ap se hota hai toh kar lo agar nahi horaha hai 10-15 min k andar mein toh phir ziada case kharab hai toh hospital le k jaate hain

Interviewer: thek hai

DM-07: us ko ziada cheezein nahi istemaal karne deite

Interviewer: aur ap ko ap k raaye mein ziada behtar kia hai aurat ghar mein bacha de

DM-07: ziada meri raaye yehi hogi ke matlab ziada hospital mein bacha paida karna chaiye ziada faida hota hai achanak aese koi emergency case hota hai toh solve kar leite hain doctor ziada deakhte hain hamare se bhi matlab joh idhar ki karti hai na workerein toh isi wajha se mera dil khafa hota hai

Interviewer: jee

DM-07: khayal sahi nahi rakhtin

Interviewer: sahi sahi

DM-07: TBA se ziada doctor ache se deakhte hain

Interviewer: hm hm hm

DM-07: magar yahan ke doctor karte nahi hai

Interviewer: hm hm jee

DM-07: waise TBA se ziada faida hota hai hospital mein

Interviewer: hm hm

DM-07: achanak aesa case hojaata hai operation ye woh toh woh log usi waqat matlab solve karte hain magar yeh karte nahi hai na usi wajha se matlab bohat saari ladies naam toh likhwaati hain magar un ko waise nahi aata hai toh matlab woh change kar leite hain

Interviewer: hm hm hm

DM-07: isi wajha se TBA se acha hospital faidemand hota hai

Interviewer: hm

DM-07: bache k liye bhi hota hai bache ki maa ke liye bhi hota hai toh yeh nahi karti hain

Interviewer: je je

DM-07: aur TBA ka ghar mein yeh matlab faida hota hai hum apne mareez ke saath support mein hote hain yeh bas faida hota hai TBA ke saath aur toh faida nahi hota woh hamare saath matlab ache se deakh karti hai mareez ke saath yeh aur toh ziada hospital pe hi bharosa rakhte hain magar hospital waale khud apna naam gira deite hain toh us mein hamara toh kasoor nahi hai

Interviewer: hmm hmm hmm

DM-07: yeh jab is k saath dusra bacha hua na is ka pehle mein aesa hua phir mein ne manah kiya tha phir bhi yeh gayi phir mein kehti thi sahi hai shayad woh badal gaye ho pehle is ko nahi deakhte thy ache se phir bhi yeh jaati thi

Interviewer: jee

DM-07: phir bhi matlab apna time zaaya karti thi ghar ka yeh woh phir idhar se pedal jaati thi phir wahan se itna dhoop mein pedal aati thi mein us ko bolti thi jaane ka faida kia ke tu jaati hai center pe un ko yeh khayal nahi aata hai ke teri haalat kaisi agar hum udhar chal ke beithi hain tum log thora taang upar karte ho toh kia kehti hain upar madam beithi hain camera mein deakh rahi hai taang upar nahi rakho taang neeche nahi rakho dikhaawa kyun kar rahi hai toh itni diqqat charhaaye se upar hoti hai toh un ko diqqat nazar nahi arahi

Interviewer: hm

DM-07: aur jab wahan ja k beithti hain un ki aakhon ke saamne phir apna dikhawa karti hain yeh nahi rakho woh andar bari doctor deakh rahi hai woh manah karti hai bache ko kuch hojaayega maa ko kuch hojaayega aur itna yeh pedal aati hai charhaaye se upar neeche agar paaon phissal jaaye ye woh toh phir woh nazar nahi aata hai toh chalo aayi hai mareez us ko ghar tak bhi pohcha k aate hain woh toh nahi karte hain

Interviewer: hm hm hm

DM-07: toh yeh mein ne sawal udhar kiya tha do dafa udhar woh kia kehti hai ek doctor k han us waqat tumhare woh thori ghar pe hota tha ghar pe masla nahi hota hai jab tum logon ne date di huyi hai tumhein pata hai falana mareez aana hai toh us ko leine ajaya karo sahi hai na agar nahi aate aur mareez

ajaata hai baad mein us ko chorte hain ek dafa toh chor k aao nahi han 10 12 katthey honge phir le k jaayeinge warna nahi toh woh khud hi chali jaati hain phir bhi yeh chali jaati thi us ko bharosa tha magar ab yeh is ka bharosa toot chuka hai

Interviewer: acha sahi sahi aur yeh bolna chah rahi hun k joh bache ghar mein ap k paida huye hain ya kisi ap k janney wale joh bache paida huye ap k ache aur bure tajurubaat ap bata sakti hain jaise ap k ghar k

DM-07: matlab bacha TBA paida karwa deity hain hai na

Interviewer: hmm

DM-07: phir woh kehti hain ap ki marzi hai

Interviewer: hm hm

DM-07: woh thora bohat tajurubaat batati hai, khane k nuske wuske batati hai, kuch hum istemaal karte bhi hain bache k liye joh hamein sahi lagta hai woh hum kar leite hain nahi toh joh hamein sahi nahi lagta hai woh phir hum doctor se mashwara kar k matlab private hi se ilaaj karwa leiti hai abhi yeh joh doctor hum bol rahe hain na us ko hum ne dikhaya tha na toh us ne matlab us ne thori dawaai li di udhar hi us ne kaha halka bukhaar hai toh woh bhi matlab bohat poorana doctor hai bachon ka hi ilaaj karta hai udhar duty us ki hai

Interviewer: jee

DM-07: hum bacha udhar hi community ki aurtein usi clinic pe le k jaate hain

Interviewer: hm hm

DM-07: bohat poorana hai bachon k hawale se hum us k paas le k jaate hain

Interviewer: sahi

DM-07: woh hamein bolega na kaagaz pe likh k deiga JPMC le k aao

Interviewer: hmm

DM-07: phir hum le k jaate hain doctoron k paas, warna hum nahi le k jaate

Interviewer: hmm un pe bharosa hai?

DM-07: un pe bharosa hai

Interviewer: jee jee sahi aur jaise ap kaheingi k is bache k sath phir acha tajurba sab kuch sahi raha

DM-07: han sab kuch sahi raha

Interviewer: Allah ka shukar hai joh pehle in ka bacha hua tha…

DM-07: woh bohat diqqat mein tha bohat pareshani thi is mein Allah ka shukar hai itna nahi hai yeh matlab hum us ne TBA ne paida toh karwa diya us ne kaha phir mein ne us ko deakha thora us ko na ek problem hogayi thi naap ki us buddhi hai na toh us ne sakt naap nahi baandhi thi dheeli thi usi wajha se thori si pareshani thi phir mein ne jaise deakha mein ne kaha isay naap ki pareshani huyi mujhe pata tha

woh doctor matlab sahi naap ka ilaaj woh karta hai phir mein foran udhar hi us ko le k gayi us ne patti ki do teen din k baad naap sahi hogayi dard tha woh bhi us ka khatam hogaya dawaai sahi di ab Allah ka shukar hai sahi hai

Interviewer: acha Allah ka shukar hai acha toh aur is ghar mein sirf in k bache huye hain

DM-07: bas is ka hi hua hai

Interviewer: sahi acha yeh bhi jaanna chah rahi hun mein k joh aurat ki raaye hoti hai jis jagha ko tarjeeh deity hain paida karne ki joh jagha hoti hai ap k liye woh eham hota hai joh un ka raaye hota hai

DM-07: hai nay eh in logon ki khud hi meri raaye toh nahi thi na magar inhon ne bola hum udhar kareinge mein ne kaha ap ko ziada acha lagta hai sahi hai phir mein ne kaha chalo sahi hai bache ko toh insaan hai na bacha toh dobaara bhi ajaata hai magar ziada ehmiat rakhti hai maa

Interviewer: hm

DM-07: maa ki zindagi achi hoyegi toh sahi hai

Interviewer: hm hm

DM-07: toh us ne kaha idhar kuch hojaayega aese waise TBA k paas mera bacha bhi chala jaayega mein bhi chali jaongi isi wajha se phir mein ne socha mein ne kaha woh soti hai yeh nuksaan hoyega us ko ziada faida lagta hai toh achi baat hai bacha toh baad mein bhi zindagi rahegi mil jaayega magar maa toh dobaara nahi milti hai toh is liye us ko sahi lagta hai toh sahi hai phir us ko khud hi udhar se pata lag gaya na toh phir yeh woh toh achanak kaise hogaya wahan se joh ultrasound karwa k aaye thy na toh us k karwa k aaye phir dobara gaye ultrasound mein ne kaha doctor bol rahi hai toh chalo ek dafa dobaara chale jaate hain us ne is ka ultrasound hi nahi deakha jaise us doctor ne bola tha waise thek teesre din paida hogaya

Interviewer: hm hm hm

DM-07: in logon ne 8 din de diye aur jao aur din hai toh joh us doctor ne likh k diya us ne likh k bhi diya tha phir bhi inhon ne dekha talak nahi phenk k de diya

Interviewer: Oh!!

DM-07: deakhna toh chaiye na hum ne kaha ap likh dou na toh phir woh samjh jaayeinge doctor hai ap bhi doctor ho toh us ne deakha hi nahi phenk diya direct us ko phenk k de diya

Interviewer: hm hm

DM-07: aur teesre din k hisaab se yeh teesre din paida hogaya

Interviewer: acha

DM-07: us ne kaha haftey k din itwaar ki raat haftey ka din guzrega yeh ajaayega duniya mein toh itwaar ka din in ka pehla din tha usi din pe hogaya toh un ko deakhna chaiye tha na phir is ka dil toot gaya is ne kaha tum dhund rahi thi sahi tha

Interviewer: hmm hmm sahi hai acha toh ap k jaise bache k pedaaish ki joh jagha hoti hai ap ka khayal aaya tha un ka khayal milte julte hain ya mukhtalif hai?

DM-07: mukhtalif hai na in ko sahi nahi laga

Interviewer: kia ap ko hispatal ziada behtar lagta hai

DM-07: behtar lagta hai magar joh abhi is ne guide kiya na is hisaab se is ka dil toota hua hai

Interviewer: hm hm hm

DM-07: us ne kaha ghar mein ziada hai nahi abhi bhi mein kehti hun ghar se ziada hospital faidemand hai magar matlab shikaayat is baat ki hoti hai khayal nahi rakhte hain

Interviewer: hmm sahi

DM-07: yeh baat hoti hai

Interviewer: hm

DM-07: isi wajha se abhi jitni bhi aurtein naam likhwaati hain karti hain kyun ghar pe delivery karti hain isi wajha se woh pareshan hojaati hain toh yeh log khayal nahi rakhte mareez ka

Interviewer: hm hm hm

DM-07: thora bohat mareez k saath pyar mohabbat se pesh aana chaiye ab doctor naam likh raha hai toh us ka yeh toh matlab nahi hai k bas tum log chor dou us ko deakho us ko kia diqqat horahi hai kia problem hai us ka khayal rakhoge toh woh aage ja k tareef karegi k center aur ziada acha hogaya hai tumhare paas mareez bhi ziada aayeinge tumhara bhi naam hoga hamari bhi sehat achi hojaayegi woh nahi karte hain log

Interviewer: hm hm hm

DM-07: isi wajha se is ka dil toot gaya hai magar nahi hum yeh nahi kehte phir dobara kisi hospital mein private hi mein daal deinge nahi toh govt bhi hain us mein daal deinge agar dobara bacha hoga toh hum pehle hi doctoron k paas karte hain

Interviewer: hm hm hm

DM-07: hamare aur sindhi nahi likhwaate hain magar hamara ghar matlab thora alag hai ziada doctor pe bharosa rakhte hain

Interviewer: hm hm hm sahi sahi aur jaisa k yeh k acha khayal rakhna chaiye aur nahi rakhte toh is wajha se bohat saari aurtein kehti hain k ghar pe paida ho

DM-07: ghar pe hi paida ho

Interviewer: us k elawa aur kuch bata sakti hain k aurtein kyun ghar ko tarjeeh deity hain

DM-07: ghar ko is liye tarjeeh deity hain ghar pe yehi hota hai na karti hain koi koi gareeb hoti hain in mein ne ziada woh kharcha kar leinge mein ne kaha kharche ki baat nahi hai apne hisaab ki baat kar rahi hun mein ne kaha kharche ki baat nahi hoti paise ehmiat nahi rakhte hain zindagi ehmiat rakhti hai

Interviewer: hm

DM-07: Allah na kare ghar pe bhi aese udhar bhi na ho us se aura age chale jaaye udhar paison ki zaroorat par jaaye paise baat nahi rakhti zindagi ehmiat rakhti hai toh isi wajha se dusre aese bolte hain joh gareeb hai wahan pe muft elaaj hota hai fees nahi deni parti aurtein aesi sochti hain magar phir apne hisaab se kar leiti hain

Interviewer: hmm sahi

DM-07: abhi is ki delivery jab huyi thi na toh is k abbu ka matlab kishti chal rahi thi paise jumme ko milte thy us waqat toh paise nahi thy na toh us ne kaha sahi Allah maalik hai toh kar di delivery hogayi ghar mein phir hum ne TBA ko dusre din de diye paise

Interviewer: sahi sahi acha thek hai sahi hai aur koi cheez hai joh ap batana chaheingi shayad hum ne poocha nahi isi k hawale se

DM-07: mein yeh chahti hun yeh joh center hai na un ko chaiye ache se pesh aana chaiye

Interviewer: hmm bilkul bilkul

DM-07: deakh matlab ladies aati hai toh doctor ladies k saath mashwara karti hain k mere ko takleef horahi hai bacha idhar lag gaya hai toh yeh un ko poochna chaiye woh bata rahi hai un ko karna chaiye yeh log nahi karti hain isi wajha se mujhe acha nahi lagta un ko aese nahi karna chaiye

Interviewer: hmm jee jee ap sahi keh rahi hain joh ap ne baatein ki woh bohat hamare liye eham hai bohat hamare matlab kaam mein madad milti hai

DM-07: abhi deakhiye teeke teeke kehte hain bachon ko nahi lagaana chaiye woh hamare kaafi log nahi lagaate hain log abhi katre bhi nahi pila rahe hain hamare sindhi magar hum ne is ko karta pilaya tha ziada in cheez pe bharosa rakhte hain abhi parso bhi phadda chal raha tha na woh polio pe nahi pila rahe thy aurtein hum ne toh manah nahi kiya hum ne khud yahan se apna bacha le k udhar team beithi huyi thi katra pila k agayi teeka bhi lagwa k agaye khud hi

Interviewer: hmm

DM-07: us ne abhi yeh keh rahe hain is ko poore teeke nahi lagaaye phir mein in ko keh rahi hun is ko poore 12 teekey lagaayeinge bukhaar aata hai toh is ko tor kar leinge hain!! magar is ko ziadi dawaa is k jism k andar rahegi toh is ko koi beemari control nahi kareigi

Interviewer: hm hm

DM-07: dawaai se faida hota hai toh yeh log kehte hain nahi par mein ne kaha mein poore teekey lagwaaongi

Interviewer: teekey ap kahan se lagwaati hain

DM-07: govt ki aati hai na team

Interviewer: paise leite hain ?

DM-07: nahi nahi free hote hain

Interviewer: free thek thek

DM-07: team aati hai jaise tum log ho waise woh govt ki aati hai na team katrey pilaane wali

Interviewer: jee jee jee

DM-07: us mein naam likha hua hai wohi teekey lagwaati hai govt ki

Interviewer: jee jee jee thek hai thek toh sahi hai toh phir mujhe lag raha hai ap ne bohat acha (16:46-16:47) toh bohat shukriya us ka aur bas inshaallah acha tha woh bas yeh sehat in ki achi rahe aur phir bhi center ka kabhi kabhi deakhein ………………

____________________________X___________________________

IDI DM-08

Interviewer: apki umar kitni hai?

DM-08: 50 tak hogi

Interviewer: taleem kitni hai apki?

DM-08: taleem nahi hai

Interviewer : apki inki saas hain ?

DM-08: jee

Interviewer : ap kuch kaam karti hain?

DM-08: nahi ghar par hi hoti hun

Interviewer : mein ap se sab se pehle ye puchna chahungi ke jo hamla aurat hoti hain usko kin kin sehat ki saholiyaat ki zaroorat hoti hai ?

DM-08: gaari ki saholat ho baaqi bas bemaari mein jin cheezon ki zaroorat ho

Interviewer : or delivery ke waqt aurat ko kin cheezon ki zaroorat hoti hai ?

DM-08: mein khud karti hun delivery ghar mein , gloves chaadar wagera us waqt chaiye hota hai

Interviewer : ap ke nazdeek ghar mein bacha karna theek hai ya hospital mein ?

DM-08: Allah ka shukar hai iskey abhi tak ghar par huwe hain to ghar par hi theek hai

Interviewer : or ghar par kyun theek lagta hai ?

DM-08: ghar par pehle pasand tha abhi to is ne naam bhi likhwaya tha PHC mein jati bhi thi lakin time nahi mila mere paas mobile bhi nahi tha or barish bhi ho rahi thi islye call nahi kar sakein unhain islye ghar par hi hogaya

Interviewer : ap k nazdeek aurat ki raaye ehmiyat rakhti hai delivery ke faisley mein ?

DM-08: jee zaroori hai

Interviewer : ap bata sakti hain aurtein ghar par kyun pasand karti hain karna ?

DM-08: bas asani se ho jata hai shayad islye

Interviewer : ap ke khayal mein koi rukawatein hoti hain hospital jane mein jiski waja se auratein ghar mein paida karna acha samajhti hain ?

DM-08: hospital mein mera dil nahi manta

Interviewer : ap kisi ko janti hain jis ne hospital mein bacha paida kiya ho ?

DM-08: jee mere samney paros mein

Interviewer : unka tajaruba kaisa raha kuch baat huwi apki un se ?

DM-08: nahi hum un ke ghar nahi gaye

Interviewer : kisi khandan wale se ?

DM-08: mera or koi khandan nahi hai idher

Interviewer : acha phir kisi or se kabhi kuch suna ho ?

DM-08: jee nahi mein ne nahi suna

Interviewer : ghar par bachey paida karney mein asani hoti hai koi masla nahi huwa ?

DM-08: nahi koi masla nahi

Interviewer : koi bacha fout ya zaaya to nahi huwa ?

DM-08: nahi nahi

Interviewer : or koi aesi baat jo hum pooch na sakein or ap batana chahein?

DM-08: or to kuch nahi

Interviewer : okay, ap ke waqt ka bohat bohat shukria.

___________________________X__________________________

IDI DM-09

Decision-maker 9 did not consent to audio-recording.

SD questions:

- Woman 9's mother-in-law
- 50 years old
- Not educated- has only read the Quran
- Homemaker

Questionnaire answers:

- Juice, fruit, and vegetables are important for a woman's health during pregnancy.
- Ultrasound and tests are also important
- For delivery, a woman needs hot milk or tea so that it helps with the contractions.
- Doctors are important but they're not at home. At home, all the planning is already done.
- Within hospitals, there are services for everything there. But it's on God where the baby happens and how it happens.
- KGH is a good hospital
- Women these days don't have a lot of strength. And these days there are more illnesses around.
- Now the men in the family understand the importance of hospitals.
- Has heard negative things- 'A woman was in labor at the hospital, but she had a stillbirth and the placenta stayed inside. KGH and JPMC had refused her, so she ended up having a stillbirth.'
- "We try our best for the baby is born at the hospital".
- Not sure why women prefer home births. They can't afford or access proper care, so they prefer to give birth at home.
- Thinking about future generations, the hospital is a better option for them, they offer good services
- 'Doctors don't inform us about the process during the delivery and why they do certain things like give an injection, etc.'
- "We are grateful that there is a PHC made for us underprivileged people".

______________________________X_____________________________

IDI DM-10

Interviewer: ap mujhey bata sakti hain apki umar kitni hai?

DM-10: beta ye to nahi pata shyad 70 75 saal hogi

Interviewer: apki koi taleem hai?

DM-10: nahi kuch bhi nahi

Interviewer: ap ka [woman] se kya taaluq hai?

DM-10: meri beti ki bahu hai

Interviewer: abhi ap kuch karti hain?

DM-10: han ghar main samosey bnati hun

Interviewer: ap ke khayal main hamal ke doran aurton ko sehat ke hawaley se kin cheezon ki zaroorat hoti hai?

DM-10: beta hum ne ye sab to nahi dekha ke sehat ke hawaley se kya zaroori hai kya khaen kya piyen bas jo milta hai kha lete hainrookha sookha sab

Interviewer: bachey ki pideash ke waqt ap ke khayal main kin cheezon ki zaroorat par sakti hai?

DM-10: mujhey nahi maloom

Interviewer: lakin jese ap ne apni bahu ka beti wagera ka dekha hoga to un ke tajarube se ap ne dekha ke kin cheezon ki zaroorat ziada parti hai?

DM-10: ye bhi mujhey nahi maloom ye ammi ke pas rhene chali jati hain mere pas bas ye pheli hi bahu hai baaqi main sab bhool gaen hun beti wagera ka

Interviewer: koi baat nahi lakin jese aksar log kehte hain TBA ko bula lete hain TBA kar deti hai sab to ap ke khayal main TBA kya karti hai ya kya kya cheezen zaroori hoti hain?

DM-10: mujhey jab pehla bacha huwa tha tab mujhey 8 bajey dard shuru huwa tha or mujhey 12 baj gay the us ne zor de de kar soojh soojh kar meri itni bari shakal hogai thi paon hath sab soojh gaya tha phir mene kaha mainn ahi kar rahin hun hato mujhse dur meri behnain brabar main ro rahi thin ke hamari bhainhumey chor kar ja rahi hai mar jaegi ye phir wo to bas Allah ka shukar 12 bajey ja kar mene bachi paida kari to bachi ka sir tha bara to wo phasa huwa tha is waja se der hogai thi mujhey

Interviewer: us ke doran TBA ne kuch dya tha ap ko khane piney ke liye?

DM-10: TBA bhi nahi thi koi nahi tha bas ghar wale the brabar main bhenen wagera khane piney ke lye chawal ka aata , makhan or jari boti wagera

Interviewer: to un se ap ke dard barh jatey the ya kya hota tha?

DM-10: dard nahi dard nahi hota bacha hote huwe kisi kisi ko hota hai mujhey nahi huwa 5 bachon main kisi main bhi aj kal larkyan sabr nahi karti bas dard utha to hospital poanchao ab wo ameer ho ya gareeb

yehi hota hai aj kal to , hamarey pas Allah ka shukar sab kuch tha lakin hum ne hospital nahi dekhey kabhi bhi phele ye sab nahi tha na ab mujhey ak 8 bajey dard utha dusrey 12 bajey ja kar meri bachi paida huwi ab to koi 10 minute bhi nahi rakhta aj kal pet nikla nahi or gaen test karwaney phir kesa hai kya hai larka hai ya larki ye sab hota hai na humey to ye pata bhi nahi tha jahil log the hum

Interviewer: jese ye apki bahu wagera agar kahain ke humey hospital main karna hai to apko aitraaz hoga is baat se?

DM-10: nahi koi aitraaz nahi hai bas Jahan ho sahi ho Allah zindagi de

Interviewer: ap ke koi ache ya bure tajarubaat hainbachey ki pidaesh ke hawaley se ap k apne ya ksi ke dekhey hn?

DM-10: bas hum to ye hi dua kartey hainke normal bacha hojay or bas Allah paak sehat de inhain sab hi yehi chate hainbas ke jaldi farig hojaen

Interviewer: ap ne kabhi kisi se suna ya dekha hai ke kisi ka bura tajruba raha ho ghar main ya hospital main?

DM-10: han huwa hai suna hoga tum ne bhi AKU walon ke wahan ak larki thi uska pehla bacha tha us ka inteqal hogya bacha to paida karwa lya us se bachey ki pidaesh ki waja se jo khoon hota hai usey wo bohat nikal raha tha uski maa ne bola ke isey khoon bohat aaraha hai to larkyon ne usko kapra daba dya ye sab AKU main hi huwa tha usey bas phir wo foat hogai bechari

Interviewer: aise sab sunti hainap to apki soch par kuch asar parta hai?

DM-10: zahir hai hota hai phir ke bas yahan nahi jao AKU nahi jao koi achi hai to koi seekhney wali hoti hainto wo galtyan kar deti hain

Interviewer: ap ki raay main aurten ghar main bacha paida karna kyu acha samjhti hain?

DM-10: mainn ahi janti bas Jahan unki marzi wahan karti hain

Interviewer: ap kisi ko janti hain jinho ne ghar main bachey kiye hon?

DM-10: han hamarey paarey main hain. Raat ko 2 bjey hi kya hai ak larka ak larki

Interviewer: ap ke khayal main kyun kya hoga ghar main kya center door par raha hoga unhain?

DM-10: pata nahi bas Allah ne saholat dedi asani dedi to karlya unhon ne ghar main us ke shohar ne paise bhi jama kar ke rakhey the kama kama kar 30 hazar ke Allah na karey bara pet hai 2 bachey hain lakin ghar par hogay

Interviewer: apko lagta hai ke kisi rukawaton ki waja se ghar main kartey hun? jese faasla ya peso ki waja se?

DM-10: han peso ka msla bhi hota hai gareeb log hain hum to hota hai peso ka masla bhi hota hai islye hum chate hainke ghar par hi phele hojay

Interviewer: or koi asi cheez is hawaley se jo ap batana chaen ya hum puch na saken ho?

DM-10: nahi bas main bol to rahi hun main to sab bhool gai

Interviewer: nahi itna sab to bataya ap ne

DM-10: han wo bas us waqt ka baqi kese huwe kese barey huwe kuch yad nahi mujhey to ab ye na waise ki biwi hai is ke bhi 2 bachey hain

Interviewer: sahi sahi theek hai, apka bohat bohat shukria.

____________________________X___________________________

IDI DM-11

Interviewer: acha apki umar kitni hai?

DM-11: 40 saal hogi

Interviewer: ap ne koi taleem hasil ki hai?

DM-11: nahi

Interviewer: apka Rishta kya hai in se?

DM-11: bahu hai

Interviewer: abhi ap kuch kam karti hain ya ghar pa hoti hain

DM-11: nahi ghar hoti hun

Interviewer: ap ke khayal main hamal ke doran aurton ko kin saholiyaat ki zaroorat hoti hai?

DM-11: doodh, makhan, teil ki maalish wagera

Interviewer: hospital ke hawale se sehat ki konsi saholiyaat ki zaroorat parti hai aurton ko ya delivery ke time?

DM-11: garam garam doodh ziada se ziada peena chaiye

Interviewer: us se kya hota hai?

DM-11: takleef thori ziada hojati hai to delivery main asani hojati hai

Interviewer: hifazat ke tor kin cheezon ki zaroorat hoti hai?

DM-11: daliya wagera

Interviewer: koi dawai wagera jinki zaroorat hoti hai?

DM-11: agar doctor kahain to theek warna nahi

Interviewer: bacha paida karney ki bhetar jaghan apko konsi lagti hai?

DM-11: jab hum hamal se hote the tab TBA sahi se karti thin abhi to hospital theek hai TBA sahi nahi karti ab

Interviewer: ab sahi kyu nahi lagti TBA?

DM-11: pehle jo hain wo TBA wo doodh wagera deti thin dehan se aram se karti thin abhi ki TBA bacha daani wagera ko nuqsan poancha deti haincheer phar deti hain andar se bacha nahi aata bahar to bohat takleef deti hain

Interviewer: aesa ap ne suna hai ke asa hota hai ya huwa hai?

DM-11: han mainne hospital ke bare main bhi yehi suna hai ke wahan bhi bacha nahi hota to aesa hi kartey hain chote operation main

Interviewer: to apko TBA theek lagti hai ya hospital?

DM-11: hospital wahan blood pressure wagera ka bhi ilaaj hota hai TBA to nahi karti na ilaaj

Interviewer: jinho ne apko ye sab kaha hospital ke bare main unka ghar ka tajruba kesa raha?

DM-11: han unka khena hai hospital main agar operation karte hain to ilaaj se theek bhi kartey hain TBA theek nahi karti phele ki dawai jo dawai anati thin wo dawaiyan hath se banati thin to wo bohat achi huwa karti thin us se na koi dard hota tha sir mainn a jism na pet latakta tha delivery ke baad kuch bhi nahi bachey ko bhi ache se saaf karti thin lakin ab ye sab nahi hai abhi ki TBA ye sab nahi karti darti hain wo kuch bhi karney se isliye abhi hospital main theek hai

Interviewer: apko itna tajruba kese hai TBA ka ap ne kisi se suna hai?

DM-11: phele yehi sab hota tha na operation hota tha na hospital wagera sab TBA hi hoti thi islye phele ye sb nahi tha bhi ki bachiyan to ak hi bachey main larkiyan burhi hojati hain hum nahi hote the , aj kal ki TBA bhi sahi nahi hain islye hospital theek hail akin wahan bDM-11i phele operation kam hote the ab ziada tar operation hi hote hain

Interviewer: ap ke khayal main bacha paida karney ki jaghan ka faisla kon leta hai?

DM-11: main apni bahu ko bolti hun inki marzi hospital main karo ya ghar main tumhari marzi hum wohi karenge jo tum kahogi

Interviewer: ap ke khayal main larkiyan ghar main kyu karti hain bacha?

DM-11: mhengai bohat hai isliye ghar main hi hojay asani se phele TBA bhi 100 200 leti thi ab to wo bhi 4 5 hazar leti hain

Interviewer: ap ke khayal main aurten hospital kyu nahi poanch pati kya waja hoti hain?

DM-11: TBA se ziada hospital sahi hai wahin karna chaiye

Interviewer: lakin jo ghar main karti hain wo kis waja se ghar main karti hain?

DM-11: darti hain shayad ke hospital main ziada dard hoga injection wagera se ghar par tel ki maalish wagera hoti hai to unhain wo sahi lagta hai asani se

Interviewer: apki bahu center kese jati thin?

DM-11: akele lakin mujhey bolti thi to hum sath jate the phir , delivery ke waqt bas mera dil bohat dhak dhak karta hai main pareshan hojati hun

Interviewer: ap ke khayal se hum center ya hospital main kuch behtri laa saktein hain?

DM-11: bas sahi ilaaj ho hamara hospital to theek hai lakin ilaaj main faida na ho to kya faida islye bas ilaaj ache se ho

Interviewer: okay shukriya

______________________________X_____________________________

IDI DM-12

Interviewer: urdu mein ap bata sakti hain urdu samjh aati hain?

DM-12: nahi samjh aati (laughing)

Supporter: samjh sakti hain bol bhi sakti hain (laugh)

Interviewer: sahi sahi han kaafi gharon mein gaye toh un ko nahi aati toh hum health worker ko bula k idhar bitha k phir interview karte hain toh agar ap chahti hain k sindhi mein karein toh sindhi mein karein

DM-12: sindhi mein hum gar mar nahi jaanta

Supporter: hum sindhi nahi Baloch hain

Interviewer: Baloch hain jee jee jee sorry sorry toh sahi hai phir hum shuru kar leite hain to hap ki umar kitni hai? Andaaze se

Supporter: abhi meri 30 hai meri bari behan 32 23 ka hai ab ammi ko 50 60 nahi hoga ?

Interviewer: hmm 50 60 thek hai

DM-12: ziada 50 se bhi ziada hoga yeh pata nahi chal raha hai

Supporter: 60 hoga abhi hum 30 k hain

Interviewer: lekin behnain lag rahi hain ap itni ziada umar nahi hogi ap ki toh acha to hap ne taleem kitni haasil ki?

DM-12: kuch bhi nahi

Interviewer: aur yeh ap ki beiti hain

DM-12: jee

Interviewer thek hai aur ap kahin pe kaam karti hain ya ghar par hi rehti hain

DM-12: ghar pe rehti hun

Interviewer: thek acha sab se pehle ap se poochna hai k joh hamla aurat hai un k liye kia sehat ki sahuliyaat zaroori hai

DM-12: 10 bache ghar mein paida kiye

Interviewer: jee jee aurton ki sehat k liye kia zaroori hoti hai us time par jab un ka bacha jaise un k hamal k doraan un ko kia zaroori hoti hai

DM-12: taaqat ki zaroorat hoti hai jab taaqat hoti hai toh bache paida hote hain jab taaqat nahi hoti toh bache kahan se paida honge

Interviewer: or jaise koi sehat k hawale se matlab jaise hispatal mein

DM-12: nahi hum hispatal kahin jagha pe nahi gay eek bachi mein ne paida ki hispatal mein delivery karwaai

Interviewer: hmm lekin ap ko lagta hai k hispatal jana zaroori hota hai

DM-12: jaana toh zaroori hai sehat k liye abhi yeh saiqa ki halaat kharab thi

Interviewer: bilkul

DM-12 is dar se mein bhaag gayi thi jab yeh choti waali paida hua jab paida kare mein ro rahi hun

Interviewer: aaye !!!

DM-12: toh mujhe dar laga kyun k hispatal acha hai koi doctor hote hain koi jagha hota hai machine wagera hota hai hum halaat mein bache paida kar sakte hain

Interviewer: hmm hmm

DM-12: joh meri choti beti hai na hum yahan hispatal mein gaye thy yahan se phas gayi bachi mein ne us ko bola cheelh dein us ne nahi kiya bachi phas gayi jab bachi paida hua toh neel par gayi us ko bichara maazoor hai ab tak 8 saal se maazoor para hai

Interviewer: Allah!!

DM-12: na khana na peena kuch bhi nahi bas dalida bana k us ko khilaate hain

Interviewer: hm hm

DM-12: hispatal isliye ache hai har cheez mehfooz hota hai hispatal hai machine hai operation hojaaye jab bare case ho sahi hai lekin yeh toh pagal k liye pe toh ghar pe sahi hai han na (laugh) yeh heart ka mareez hai us ko bolti hun jao choti wali hispatal mein paida karne keh rahe hain nahi lekin mashallah aadhey ghantey mein paida hua na is ko yeh nahi pata chala k mera bacha kaisa hai keh rahi hai k ami paida hua mein ne bola han paida hua

Interviewer: laugh

DM-12: baad mein pata chala k mera bacha paida hua lekin hispatal bohat sahi hai lekin hamara poorana zamana toh ghar mein bacha paida karte thy hispatal hi nahi tha check up bhi nahi tha

Interviewer hmm

DM-12: Mashallah mein ne 10 bache paida kiye

Interviewer: Mashallah aur ap ka tajaruba us k sath sahi raha

DM-12: sahi raha han ab eke k bacha paida karte hain kehte hain qamar ki dard taangon mein dard ab pata nahi kahan ki dard lekin hum 10 bacha paida kiya isliye toh taaqat ziada hai mere ko us k liye sehat k cheezon mein taaqat ki who sabziyon mein , jaise saalano mein sab cheez mein taaqat tha abhi toh nahi hai

Interviewer: hmm hmm hmm

DM-12: isliye

Interviewer: aur jaise delivery k time pe jab bilkul bacha paida horaha hota hai us waqt kia zaroori hoti hai aurat k liye

DM-12: hamare 8 bache meri maa k haath mein hua tha

Interviewer: hmm hmm

DM-12: or do bache ek nida k paas hua tha sab se chote 15 saal ka hai ya 14 saal ka hai

Interviewer: acha toh kaafi time se woh udhar kaam karti hain

DM-12: udhar kaam karte hain hamare se toh pehle aaye thy hum pehle yahan pe nahi thy na hamare 14 saal k larke is k haath mein hua tha

Interviewer: sahi toh ap ka tajaruba un k saath acha raha

DM-12: jee jee

Interviewer: mera bara beta hai us k bete k sath dosti us k bache itne thy na ek itni si thi ek is se bari thi meri dono beti k paas quran shareef parha hua hai

Supporter: jaise ek hamare ghar ki bahu hai who mere cousin ki hai

DM-12: lekin sahi hai mashallah baat karna beithna uthna tameez sab sahi hai

Interviewer: acha achi baat hai toh delivery k time pe jaise aurat ko kia jaise hispatal mein kia zaroori hoti hai us k paas kia hona chaiye

DM-12: hona chaiye is k saath ho yeh injection wagera dripein yeh lagta hai sahulat is ki ziada hota hai lekin sab cheez maujood hai wahan pe yeh ghar mein paida hua ghar mein teeke lagaate hain lekin is mein toh taaqat hai tab bache paida karte hain ab isey taaqat hai in logon ko ek bache paida karte taaqat hi nahi hai

Interviewer: hmm hmm

DM-12: han na .. yeh mard mein se ek bacha paida kiya aese kehte haaye meri qamar , meri taang ek bacha paida kiya

Interviewer: jee jee

DM-12: hum log pehle ek jaanein thy 10 10 bache sambhala han ab aese karte hain nahi cheezon mein taaqat nahi hai yeh makhan yeh desi ghee sab khaate thy yeh log khaate nahi hain ab yeh joh choti mur bana k khaate thy ghee k desi ghee mein khaate thy

Interviewer: jee jee sahi sahi

DM-12: pehle hum log ki bachiyan pait mein thy toh hum logon ne taaqat ki goliyaan wagera bhi nahi khaaye hain

Interviewer: hm hm hm

DM-12: mashallah bacha paida kiya sehatmand tha

Interviewer: toh ap jaise pehle apne saare bache ghar pe paida kiye Dr. Aunty k sath kiya ap abhi kyun kehti hain k hispatal behtar hain

DM-12: hispatal behtar hai par halaat aese hote hain na bimaari ziada horaha hai bache sahi nahi hai koi therey hota hai kabhi paaon se aata hai kabhi ek side se aate hain yeh toh peet se aaya hai peeche se yeh

Interviewer: acha

DM-12: han .. yeh nahi hai poora hissa han jab hispatal walon ne kaha hai yeh aese ulte hain lekin aese ulte nahi thy ek paaon se yeh peeche se aaya tha

Interviewer: oh ok aur ghar pe hi

DM-12: ghar pe huyi mashallah

Interviewer: or koi masla toh nahi

DM-12: nahi kuch bhi masla nahi hua

Interviewer: un ka scissor nahi hua tha normal tha

DM-12: han normal hua lekin mashallah hua sahi

Interviewer: acha acha mashallah

DM-12: lekin 10 bache mein ne paida kiya 6 bache is ne paida kiye ghar pea ur 5 meri bari beti ne paida kiya ghar pe ek dusra hai choti wali sab se us ka scissor se huye thy

Interviewer: us k scissor k huye thy

DM-12: scissor se huye thy is ki hadiyaan jam hai na isliye hua

Interviewer: acha

DM-12: haddi ki wajha se

Interviewer: sahi toh phir jaise ap ne bohat saare logon se baat ki aur bohat saare logon se suna hai ap ko behtar lag raha hai k hispatal

DM-12: mein keh rahi hun aaj kal k zamaane mein hisptal sahi hai

Interviewer: hispatal sahi hai

DM-12: hispatal sahi hai deakho bache is tarhan horahe hain kisi ko jhatka par raha hai kisi ko dora par raha hai kabhi kaisa horaha hai

Interviewer: hmmm

DM-12: yeh hispatal wale control kar sakte hain ghar wale toh nahi kar sakte han isliye aaj kal k zamaane mein hispatal bohat sahi hai

Interviewer: sahi sahi acha toh ab mujhe poochna tha k ap k khud k koi ya ap kisi ko jaante ho jin k ache aur bure tajarube hon woh hispatal mein karne se

DM-12: ghar se bahar nahi nikalti hun pehli baat toh yeh hai (laughing) bas din raat apne bachon k sath

Interviewer: acha sahi sahi thek hai

DM-12: deakho ache nahi hai kisi k ghar pe jao toh kaheinge yeh kyun aaya

Interviewer: hm hm

DM-12: is wajha se hai na mein bahar nahi nikalti khuda na khuwaasta kisi haadsa hoye kisi ki fout hoye kisi ka bacha hoye ek din mein chale jaata hai us k baad mein nahi jaati lekin aapas mein baatein banaate hain mein kisi k ghar pe jaati nahi pehli baat yeh hai k duri kia kar raha hai kia nahi kar raha hai bache kaise paida kar rahe hain kia bana rahe hain kia nahi mein bahar nahi nikalti

Interviewer: chalein ap k khaandaan mein jitney logon ne bache paida kiye toh un k saare jaise hispatal ya ghar mein

DM-12: meri choti nandein bari nandein us ki bhi operation se scissor hua tha

Interviewer: acha

DM-12: aur us se choti hisptal civil hispatal mein hua tha

Interviewer: kaisa gaya phir tajaruba

DM-12: han yeh hispatal mein hua tha na scissor hua tha aur us se chote us k bache k ghar pe paida hoy eek toh nida k haath mein hua tha sab se bare toh gaaon mein hua tha us se chote woh toh Balochistan mein tha us se chote us k bache nahi hai us k bache daani k anddey (eggs) chote hain

Interviewer: acha

Supporter: bache k dawaai nahi hote?

DM-12: nahi nahi keh rahe hain is k anddey (eggs) chote hain elaaj bhi kar rahe hain lekin kyun sahi nahi hai abhi bhi us ne AKU k andar LNH mein us ne test wagera kiya us ka miyaan ka kiya us ka kiya yeh reportein saare bataaein is k anddey (eggs) chote hain

Interviewer: acha

DM-12: keh rahe hain anddey (eggs) goli wagera se bare hojaate hain kyun k is k husband yahan pe nahi hai 2 saal k baad aatey hain bahar se

Interviewer: hmm hmm

DM-12: abhi 3 maheeney mein aaye balke isi pehli mein wapis chale jaayeinge

Interviewer: acha acha

DM-12: yeh bichaare itna rau rahe hain lekin bache nahi ho rahe

Interviewer: hmm mujhe lagta hai agar koi us ka hal hota toh hispatal k log ap ko bata sakte

DM-12: sahi han yeh hospital liaqat mein ja rahe hain check up kar k dono miyaan biwi dono ja rahe hain yeh keh rahe hain ultrasound wahan pe bhi yeh aaya aur dusri jagha pe kala kot mein bhi gaya wahan pe yeh bhi aaya k anddey chote hain baby k anddey chote hain isliye

Interviewer: hmm lekin us ka elaaj nahi pata

DM-12: yeh keh rahe hain pata nahi konsi taarikh mein bare bare test ajaate hain us k baad phir sochte hain k kia karna hai

Interviewer: hmm

DM-12: isliye

Interviewer: hmm sahi acha toh hispatal wale sahi hon

DM-12: mein keh rahi hun hispatal waale sahi hain agar koi cheez maujood ho wahan pe

Interviewer: koi aese bure tajarube huye hain jis ki wajha se ap kehte hain k hispatal sahi hai

DM-12: han meri nanni thi bari wali

Interviewer: un ko kia hua

DM-12: yeh joh nahi hai kehte hain hispatal ache nahi hai yeh toh roz kabhi deakho JPMC mein hai kabhi deakho kahan pe hai jaise mareez ho dusre mareez ho saare ko le k jaate hain

Interviewer: dusron ko bhi le k jaate hain

DM-12: han le k jaate hain

Interviewer: acha

DM-12: woh chota hai na foran kehti hai hispatal mein le k jaaon ghar pe koi nahi hai mein yeh keh rahi hun hispatal bohat sahi hai bacha paida karna kisi bimaari ki wajha se kyun k test wagera sab hoga na phir pata chalega k bimaari ka hal kia hai

Interviewer: biluk bilkul

DM-12: han lekin yeh meri pagal keh rahi hai hispatal acha nahi hai us ka heart hua tha na isliye

Interviewer: jee acha ap bata sakti hain joh aurat hispatal ko tarjeeh deity hai un ka joh raaye hota hai bacha kahan paida karna hai woh zaroori hoti hai ap k liye un ka joh raaye hota hai jaise ap ki beti kahein mujhe ghar pe karna hai un ki raaye ap k liye eham hoti hai

DM-12: han lekin dusra beti ko nahi is k liye yeh hispatal mein sahi hai

Supporter: dusri ko kia kehti hai

DM-12: woh mashallah sahi hai

Interviewer: han woh hispatal mein kare

DM-12: han hispatal mein karegi

Supporter: woh ghar mein paida kare un k liye achi baat hai lekin is k liye achi nahi hai

DM-12: han heart ki wajha se na 13.32

Interviewer: heart ki wajha se

DM-12: lekin ek maheena joh hota hai na bache joh pait mein hota hai hai 9 maheeney tak

Interviewer: hmm hmm

DM-12: 13:40 mera bhi jeena haram

Interviewer: hmmm

DM-12: is liye kehti hun bachi band kar k hojaaye mein ne bhi bola band karo bas mujhse nahi hota bardaash isliye

Interviewer: dusri beti hispatal mein hi karti hain

DM-12: choti wali sab se choti

Interviewer: choti wali

DM-12: sab se choti wali

Interviewer: acha aur dusre kahan pe karte hain

DM-12: yeh gaaon hai sab se bari gaaon hai

Supporter: hum teen behnain shadi shuda hain

Interviewer: sahi sahi sahi sahi thek hai aurtein kyun phir ghar ko tarjeeh deity hain bache paida karne k liye

DM-12: shouk hota hai kisi ka toh shouk hota hai koi isi tarhan darte hain hispatal mein na jaana han na joh zaroori hai wahan pe jana chaiye na

Interviewer: sahi sahi sahi

DM-12: joh Allah taalah ghar mein paida kareinge woh bhi sahi hai

Interviewer: is k elawa log kuch kehte hain k joh faasle ki wajha se hispatal nahi jaate hain paison ki wajha se ap ko kia lagta hai

DM-12: paison ki toh hum log fikar nahi karte jaan hai jahan hai

Interviewer: acha

DM-12: sahi hai paison ka ek toh jaan hi chala jaata hai bheek maange lekin sehat k liye maangna parta hai na

Interviewer: sahi sahi acha ap ko lagta hai aur koi rukaawatein jis ki wajha se aurtein kehti hain hum ghar pe hi kareinge

DM-12: hai ek 15:27 – 15:34 andaaza kia hai sirf mein rahon meri beti ka waaris

Interviewer: acha aur koi cheez hai joh ap batana chahein shayad hum ne poocha nahi ho ap se

DM-12: nahi nahi

Interviewer: thek hai ok bohat shukriya

______________________________X____________________________

IDI DM-13

Interviewer: apki umar kitni hai?

DM-13: 31 saal

Interviewer: apki taleem kitni hai?

DM-13: 7 class

Interviewer: apki mulazimat kya hai abhi?

DM-13: cement ki mazdoori karta hun

Interviewer: Inn se apka kya rishta hai?

DM-13: mai shohar hun unka

Interviewer: apki nazar mein ak aurat ke hamal ke doran sehat ki kin saholiyaat ki zaroorat hoti hai?

DM-13: fruit wagera doodh wagera ki

Interviewer: un ke ilawa hospital center ya khoon wagera jesi kin zarooriyaat ki zaroorat hoti hai apki nazar mai

DM-13: hamara to normal huwa hai bilkul kisi cheez ka masla nahi huwa hum ehtiyat kartey thy na islye

Interviewer: Alhumdulillah sir lakin mein aam tor par pooch raha hun ke apki nazar mein hamal ke doran kya cheez ehtiyat rakhti hai?

DM-13: jab ho jata hai tab thore masley hote hain to le kar jate hain

Interviewer: acha delivery ke doran kin sahooliyaat ki zaroorat hoti hai?

DM-13: bas ye hi dua hoti hai ke Allah donu ki sehat rakhey bas

Interviewer: or hospital ke hawale se kin cheezon ki zaroorat hoti hai?

DM-13: abhi tak to gaye nahi bas dawai wagera hi le kara ate hen 3 bachon mai bs wahan jate hain to wo kehte hain ke theek hai bilkul dawai dedtey the bas

Interviewer: 3 bachon mai center gaen thi ya sirf is teesrey wale mein?

DM-13: bas isi bachey mein

Interviewer: koi asi cheez jis mai hum center mai tabdeeli la saktey hain?

DM-13: wahan ke doctors ziada tar khete hen theek hai bacha, to hum qareeb hi kisi private mai chale jatey hen wahan aana jana to ye masla ho jata hai wahan se ilaaj karwa bhi lo to dubara private jana hi parta hai

Interviewer: or koi faasley ka masla hota hai?

DM-13: dekhen unki gaari ka hota hai jese kbhi aai ya 12 baj gay aate aate to ghar par 2 or bachey hote hain wo akele hojate hain

Interviewer: or koi asa masla jo hum theek kar saktey ho hospital ya center mein?

DM-13: bas jee yehi tha jo keh dya

Interviewer: apki raaye mein aurat ka faisla eham hai delivery ke hawale se?

DM-13: inko jab acha lagta hai sahi lagta hai tab ye kheti hain. Ghar par hi mene dupher mai call ki thi inhen phir jab mai ghar aya to meri walida aaen to ghar par sehat mand aulda hogai MAShaAllah ye keh rahi thin ke raat mai hi mujhey halkey dard ho rahe the bas mai chalti rahi chalti rahi kisi ko nahi bataya kyu ke mujhey pata tha jo mamla hoga wo ab subah hi hoga

Interviewer: to apki raaye mein unka faisla eham hai?

DM-13: jee bilkul wo hamare sath ak zindagi guzar rahi hen to ak tarfa faisla kese le saktey hain

Interviewer: ap ke khayal mai ghar ko tarjeeh kyu deti hain aurten delivery ke lye

DM-13: pata nahi nas unko jab lagta hai hospital to hospital warna ghar par

Interviewer: jese aksar aurten kheti hen hamara aane jane mai time or pese lagtey hain ya ilaaj meion paise kharch hote hain ya dar lgta hai to apko kya lagta hai kya wujoohat hoti hen ghar par bacha karne ki?

DM-13: abhi ye dekhen na sarkari hospitals mai bas zara si takleef dekhenge nahi jald bazi karenge chota ya bara operation karney ki to aurten operation se darti hain

Interviewer: ghar par paida karney ke koi nuqsanaat apki nazar mein?

DM-13: kuch bhi nahi Alhumdulillah mere 2 bachey ghar par huwe hen bilkul kheryat se

Interviewer: to phir bhi ap hospital ko tarjeeh dete hain asa kyu?

DM-13: dekhen wo kheti hai mai bilkul theek hun mujh se ghar par hojaega bacha to mai ab kya kahun mein to kehta hun call karta hun ke chalo hospital lakin bacha us ne karna hai wo Jahan bole mai lar to nahi sakta na

Interviewer: theek hai or faisley kon karta hai ghar mai bachey ki pedaaish ke hawaley se?

DM-13: ziada tar mai hi karta hun faisley lakin is se zaroor puch leta hun ke mein ye kar raha hun

Interviewer: or kuch asa jo maine poocha na ho or ap batana chahte ho?

DM-13: nahi jee bas

Interviewer: okay jee bohat shukria.

______________________________X_____________________________

IDI DM-14

Interviewer: Aap mujhey apni umar bata sakti hain?

DM-14: taqreeban 70 80

Interviewer: ap ne taleem hasil ki hai?

DM-14: Quran Shareef parha hai

Interviewer: or inki kon hain aap?

DM-14: ammi

Interviewer: ap kuch karti hain ya ghar mai hi hoti hain?

DM-14: ghar par aalu bechti hun

Interviewer: ap ki raay mai hamal ke doran aurton ko kin saholiyaat ki zarorat hoti hai?

DM-14: khane peeney ki cheezen gizaaein dawai wagera agar pese hote hain to le kar dete hain koi masla hota hai to hospital bhi jate hain

Interviewer: or hospital ke hawaley se koi saholiyaat ki zaroorat?

DM-14: islye to jate hain naam likhwatey hain ke doctors ko kamzori wagera mhesoos ho to hospital mai jaon naam likhwa lun dawai wagera jo hai wo free mai miley gareeb log hain isliye jatey to hain hospital naam likhwatey hain jab iska ultrasound karwaya to us mai pata chala ke bacha to sookha para hai mai bhi wahin mujood thi mujhey bhi dikhaya tha unhon ne lakin na koi drip di unhon ne na bataya ke isey zaroorat hai phir jan mai madap wali TBA ke pass gai to wahan mujhey pata chala ke bachey mai paani khatam hogaya hai

Interviewer: or ap ke khayal mai pedaaish ke waqt kin saholiyaat ki zaroorat hoti hai?

DM-14: itna to mujhey nahi pata hum ne 5 bachey paida kye hain ghar mai hi TBA ko bulatey hain wo hamari achi se pedaaish karti hain bachey ki wo paani wali theli or garam garam cheezen deti hain kuch mai mehnat karti hun or wo bhi sath mehnat karti hai to mera bacha asani se aajata hai

Interviewer: or wo apko batatay hain ke mai garam cheez de rahi hun ya is se kya hoga?

DM-14: han batata hai doodh mai gur daal kar deti hai jin se dard barhta hai

Interviewer: ap ke khayal mai bacha paida karni ki behtreen jagha kya hai?

DM-14: ghar

Interviewer: kis waja se?

DM-14: hum jo gaon wale hain na humey ghar mai sukoon ziada milta hai meri behan ne bhi ghar mai kye hain Allah ka shukar hai wo bhi sehatmand hai or bachey bhi

Interviewer: sukoon ghar mai kese milta hai jo cheez center ya hospital main nahi mil pata?

DM-14: bas ghar mai hi sukoon se ho jata hai TBA aati hai wo jese boley ke ye karwana hai ase karwana hai ya kuch bhi

Interviewer: ap ne delivery ke doran ap ne koi ache ya bure tajrubaat suney hain kisi apney se ya muhallaey mai?

DM-14: jee sune to hain TV mai bhi ay din dekhtey hain lakin wo to Allah ki marzi hoti hai har cheez mai us mai ap or mai kya kar saktey hain

Interviewer: ap ke ghar mai kisi ne hospital mai bacha paida kya hai?

DM-14: iski dewraani ne

Interviewer: ap ne un se pucha ke unka kesa tajruba raha ya bataya ho unho ne?

DM-14: nahi ase sawal mai puchti hi nahi kisi se meri bari beti ne 7 bachey ghar mai kye hain abhi 8th bacha dusrey shohar se huwa uski halat kharab thi to wo us ne hospital mai kya warna 7 bachey TBA se bilkul sehatmand huwe

Interviewer: ap ke khayal mai delivery ke jaghan ka faisla aurat ka karna ahem hai jese wo agar ghar mai karna chay ya bahar karna chay?

DM-14: to wo hum us par chor dete hain

Interviewer: apni raay batata hain ap?

DM-14: meri raay to yehi hai ke ghar par ho to acha hai

Interviewer: nahu jese apki beti kahe mujhey hospital mai karwana hai to ap kya kahengi?

DM-14: to hospital mai jay bhaley

Interviewer: ap ki raay mai auraten ghar ko kyu tarjeeh deti hain hospital jane mai koi rukawaten hoti hain?

DM-14: wo to uski marzi jab insan ke sir par aati hai to paise ko nahi dekhtey paisa kahin se bhi aajata hai insan ki zindagi se upper kuch nahi

Interviewer: jese ap ne kaha sukoon ghar mai milta hai to asi or kya wujoohat lagti hain apko ghar par bacha karney ki?

DM-14: hum gaon wale hain na humey ghar par hi bhetar lagta hai sher wale boltey hain ke humey hospital le jao humey injection wagera lagega to humey zor bhi nahi dena parega or acha khud ba khud ho jaega lakin hum khete hain apne zor se bachey paida karen to sahi hai

Interviewer: is ke ilawa or koi rukawaten lagti hain apko?

DM-14: mene phele jo naam likhwaya tha bachi ka apko bataya na mene bas ke gari ke liye minatten karna parti thi

Interviewer: bohat si auraten kheti hain ke kaafi masael ki waja se hum center nahi ja sakey to apko kya lagta hai kon se masael hote hain?

DM-14: dusron ka humey nahi pata wo kya boltey hain hamari beti jis ghar mai hai wahan iska susar dewar hai mai maa hun baap hai to jab bhi asa koi masla is ke sath hota hai iska dewar phone karta hai wahan ke jaldi ao hamari bhabhi ko le kar jao peso ka koi masla nahi bas hamari bhabhi ko dekho

Interviewer: acha ap center jati thi to center mai koi asi bat jo apko lagta ho ke hum tabdeeli la saktey hain us cheez mai?

DM-14: ak ye ke gaari ka masla or dusra jo mene apko bataya ke unhon ne humey bataya nahi ke buccha sukhar raha hai kamzor hai to uska ilaaj shuru kartey zindagi to Allah dene wale hain lakin wo koshish to karen

Interviewer: ap keh rahi hain ke ap hospital kabhi gai nahi lakin apki soch kya hai hospital ke hawaley se

DM-14: dar to mujhey bs Allah se hota hai baaqi hum to beti ko kehte hain ke wo ghar mai karey lakin agar wo hospital jana to uski marzi hai us mai bhi

Interviewer: or kuch aesa jo hum ne poocha na ho lekin ap batana chahti hun ya kuch aesa jo hum center mai tabdeeli la sakein

DM-14: nahi bas wohi jo mene bataya

Interviewer: okay shukriya

_____________________________X___________________________

IDI DM-15

Interviewer: apki umar kitni hai?

DM-15: 32

Interviewer: ap ne kitni taleem hasil ki hai?

DM-15: Quran Sharif

Interviewer: ap donu ka kya Rishta hai?

DM-15: ammi hun iski

Interviewer: ap kuch kaam karti hain?

DM-15: nahi ghar mai hoti hun

Interviewer: ap ke khayal mai hamal ke doran aurat ko kin sehat ki saholiyaat ki zaroorat hoti hai?

DM-15: hum log calc pee lete hain aur machli pakatay hain

Interviewer: aur koi dawai wagera?

DM-15: nahi hum lagatey hi nahi hain drip wagera. Pheli bar tou hum logon ko itna bhaag dor nahi karna parta apna khud dard hota hai aur 8-10 minute baad bacha hojata hai. Mere 5 bachey asi hi huwe hain ghar pe.

Interviewer: tou ap TBA ko bulati thin?

DM-15: jee

Interviewer: usi hawaley se delivery ke din kin cheezon ki zaroorat par sakti hai?

DM-15: pata nahi hamarey ghar mai huwe hain sab bachey

Interviewer: ap ke khayal mai aurat ko kis jagha bacha karna chaiye

DM-15: ghar pe, humaray saray bachay ghar pe hue hain.

Interviewer: kya faida nazar aata hai apko ghar par?

DM-15: Alhumdulilah hmare 5 bachey hue hain aur paanchon sahi thay ghar par hi huwe hain 5 10 minute dard hota hai or bachey ghar mai hi hojate hain. Kaheen jana nahi parta hum ko

Interviewer: apko ghar mai kya cheez sahi lagti hai?

DM-15: apne ghar mai chal phir saktey hain

Interviewer: Aap ne koi ache bure pideash ke doran ke tajrubaat sunay hain kisi apne ya kisi muhalley mai se?

DM-15: nahi

Interviewer: apko kya lagta pedaaish ke waqt jagha ka faisla aurat ka karna ahem hai?

DM-15: han ahem hai inki marzi hai ye janen inka miya jane. Mei iss ko kehti hoon 'tumhe kamzori hoti hai tou jaya karo'

Interviewer: baki apko kya lagta hai kin wujoohat ki bina par auraten ghar ko tarjeeh deti hain?

DM-15: nahi majboori mai to sab jaghan jana parta hail akin hum log ghumney gay wahan se aa nahi sakey bas

Interviewer: ap ke khayal mai or kya rukawaten ho sakti hain center ya hospital na jane mai?

DM-15: aksar aurat ko dard nahi aata 9 mahine 10 mahine khatam hone ke bad bhi to hospital mai doctors karwa dete hain warna phele to dard aajata tha to asani se ghar mai hojata tha

Interviewer: or ap kis cheez ko tarjeeh deti hain k delivery ke waqt kya zaroori hota hai

DM-15: paid wagera dawai drip wagera lakin ghar par karney se hmarey 5 bachon mai Allah ka shukar ak injection bhi nahi laga

Interviewer: or koi cheez jis m ai hum hospital ya center mai tabdeeli la saktey ho ap ke khayal mai?

DM-15: nahi

Interviewer: ap center par aai thin sakina ke sath?

DM-15: nahi

Interviewer: or kuch aesa jo ap batana chahein or hum pooch na saken ho isi hawale se?

DM-15: nahi

Inyerviewer : or koi tajruba bura ap ne suna hai?

DM-15: bohat se bachey foat bhi ho jate hain na bas wo to Allah ki marzi

Interviewer: apki beti ka jo bacha zaya huwa apko kya lagta hai kya waja thi?

DM-15: wo to har koi ye sochta hai ke us bachey ko kya huwa achanak chalti phirti thi or bacha mar gaya soch to aati hai na ke aesa kya huwa achnk lakin hum kya kar saktey the unhon ne kaha ke beti ko bacha lo

Interviewer: is ke bad apki soch mai koi asar aya center ya hospital ke hawaley se?

DM-15: bas hum kya kar saktey the

Interviewer: or is ke bad bhi apko lagta hai ke center mai karwana chaiye bacha?

DM-15: han AKU mai hi file bnwaenge file to banwana paregi

Interviewer: okay jee shukriya

____________________________X___________________________
